# Engineered synbiotic microspheres enable system-level reprogramming of the gut-immune-vascular axis in chronic inflammatory vascular disease

**DOI:** 10.1126/sciadv.aeg1718

**Published:** 2026-11-04

**Authors:** Lin Shen, Mengqian Zhai, Zhangyu Yang, Junchao Yu, Ziyu Pang, Zijian Fang, Xingru Ding, Jiahui Xu, Yingtao Hu, Shiji Fang, Gaofeng Shu, Yang Yang, Lingchun Lv, Susu Zhang, Jian Xu, Jianhua Wu, Weiqian Chen, Chenying Lu, Minjiang Chen, Jiansong Ji

**Affiliations:** ^1^ Zhejiang Key Laboratory of Imaging and Interventional Medicine, The Fifth Affiliated Hospital of Wenzhou Medical University, Lishui 323000, China.; ^2^ School of Laboratory Medicine and Bioengineering, Hangzhou Medical College, Hangzhou 310053, China.; ^3^ Clinical College of The Affiliated Central Hospital, School of Medcine, Lishui University, Lishui, 323000, China.; ^4^ Department of radiology, Lishui Central Hospital, The Fifth Affiliated Hospital of Wenzhou Medical University, Lishui 323000, China.; ^5^ Department of Cardiology, Lishui Central Hospital, The Fifth Affiliated Hospital of Wenzhou Medical University, Lishui 323000, China.; ^6^ Department of Interventional and Vascular Surgery, Lishui Central Hospital, The Fifth Affiliated Hospital of Wenzhou Medical University, Lishui 323000, China.

## Abstract

Chronic inflammatory vascular diseases are increasingly recognized as systemic disorders sustained by maladaptive cross-organ immunometabolic coupling, yet current therapeutic strategies remain largely lesion-centered and fail to address upstream drivers of disease persistence. Here, we identify the gut-immune-vascular axis as a system-level pathogenic unit that integrates gut microbial dysbiosis, intestinal barrier dysfunction, metabolic imbalance, and vascular immune activation into a self-reinforcing pathological circuit. Through integrated clinical profiling and cross-species validation, we demonstrate that disruption of this axis is characterized by coordinated probiotic depletion, expansion of proinflammatory taxa, translocation of microbial endotoxins, accumulation of microbiota-derived metabolites, and macrophage polarization toward a proinflammatory phenotype along the gut-vascular continuum. To therapeutically target this axis, we engineer enzyme-responsive synbiotic microspheres that codeliver probiotics, a microbial metabolic pathway inhibitor, and a prebiotic within a protective polymeric matrix, enabling site-specific intestinal activation and coordinated microbiota-metabolic modulation. This engineered system restores intestinal barrier integrity, reprograms microbial ecology and metabolite output, suppresses immunometabolic dysfunction, and robustly attenuates pathological vascular remodeling across cellular, murine, and large-animal models, with in-stent restenosis serving as a representative disease context. Collectively, this work establishes axis-oriented immunometabolic reprogramming as a generalizable therapeutic paradigm and highlights engineered synbiotic platforms as scalable tools for system-level intervention in chronic inflammatory diseases.

## INTRODUCTION

Chronic inflammatory vascular diseases have traditionally been conceptualized as localized disorders driven by lesion-specific injury and maladaptive vascular repair (*1*). However, accumulating evidence suggests that vascular pathology is sustained by systemic processes extending beyond the vessel wall, involving coordinated interactions among metabolic, immune, and barrier tissues (*2*). In particular, perturbations in the gut microbiota and its metabolic output have emerged as powerful modulators of host immunity and vascular homeostasis, yet these effects are typically studied in isolation and rarely integrated into a unified disease framework (*3*). As a result, current therapeutic strategies remain largely lesion-centered and fail to address upstream, cross-organ drivers that reinforce chronic vascular inflammation and remodeling.

Cardiovascular disease (CVD) remains the leading cause of global mortality, with coronary heart disease (CHD), driven primarily by acute myocardial infarction, accounting for ∼17.9 million deaths annually, nearly 30% of all deaths globally (*4*). Atherosclerosis underlies most CVDs; the rupture of unstable plaques leading to acute coronary narrowing/occlusion represents the principal mechanism of fatal CHD events (*5*). Percutaneous coronary intervention (PCI) is the cornerstone therapy for rapid revascularization and myocardial salvage, with more than 1.4 million procedures performed in China alone in 2023, reflecting a sustained annual increase (*6*). Stent implantation has emerged as the dominant PCI modality due to its superior ability to prevent elastic recoil and maintain luminal patency; however, its long-term benefit is critically undermined by in-stent restenosis (ISR) (*7*, *8*). ISR develops in more than 10% of patients and is associated with a markedly increased risk of recurrent myocardial infarction and mortality, particularly in the absence of effective intervention (*9*). ISR has long been regarded as a localized vascular complication; however, its high recurrence rate and limited therapeutic durability suggest the involvement of upstream systemic mechanisms that extend beyond the stented lesion itself.

Despite extensive investigation, the mechanisms underlying ISR remain incompletely understood. ISR involves multiple pathological processes including endothelial dysfunction, inflammatory activation, atherosclerotic plaque destabilization, and thrombosis. Rather than arising from a single initiating event, ISR represents a highly dynamic and progressive pathological continuum in which the upstream drivers and mutual coupling of these processes have yet to be elucidated. Growing evidence suggests that gut microbiota and their metabolites may simultaneously participate in and amplify these pathological alterations at multiple levels (*10*, *11*). Microbial dysbiosis can weaken intestinal barrier integrity, promote endotoxin translocation, disrupt lipid and bile acid metabolism, and exacerbate systemic inflammation, thereby exerting sustained effects on vascular homeostasis (*12*). Specifically, microbiota-derived lipopolysaccharide (LPS) induces intestinal permeability (“gut leakage”), aggravating endothelial injury and inflammatory responses; dysregulated microbial metabolism elevates circulating low-density lipoprotein (LDL) levels, accelerating plaque formation; and trimethylamine *N*-oxide (TMAO) promotes foam cell accumulation and platelet activation, increasing the risk of plaque rupture and thrombosis (*13*). Collectively, these cross-organ and multipathway interactions are conceptualized as the “gut-plaque axis,” which may constitute a mechanistic basis driving ISR initiation and progression.

Within this framework, ISR is no longer viewed as a localized vascular response but a systemic pathological consequence of disrupted gut microbial homeostasis. Microbiome profiling studies indicate that patients with ISR exhibit pronounced alterations in gut microbial composition. Stent-associated atherosclerotic plaques contain considerable amounts of gut-derived microbes and metabolites (*14*–*17*). Animal studies further demonstrate that gut microbial status strongly influences ISR susceptibility, with fecal microbiota transplantation bidirectionally modulating ISR progression across models (*17*, *18*). As a naturally dominant probiotic residing in the healthy intestine, *Lactobacillus reuteri* (*Lac.r*) suppresses pathogenic bacterial growth, enhances short-chain fatty acid (SCFA) production, and modulates immune polarization, thus exhibiting anti-ISR potential in vitro (*19*–*21*). However, oral administration of *Lac.r* alone exerts limited inhibition of TMAO biosynthesis in vivo, rendering it insufficient to suppress sustained activation of the gut-plaque axis at its metabolic source. In contrast, 3,3-dimethyl-1-butanol (DMB), a structural analog of choline, preferentially inhibits microbial choline trimethylamine-lyase (CutC/CutD)–mediated trimethylamine (TMA) production while exerting minimal effects on the distinct carnitine oxygenase (CntA/B) pathway or bacterial viability (*22*, *23*). Integrating probiotic-mediated restoration of gut microbial homeostasis with selective metabolic inhibition of TMA production therefore provides a rational strategy for comprehensive reprogramming of the gut-plaque axis and durable prevention of ISR.

This study developed enzyme-responsive synbiotic microspheres integrating microbiota modulation, metabolic inhibition, and intestinal targeting within a single platform. Sodium alginate–β-glucan–cross-linked microspheres coencapsulating *Lac.r* the TMAO inhibitor DMB, and inulin (Alg-βG-*Lac.r*-DMB-Inu MS) enable site-specific activation in the gut while protecting probiotics and suppressing pathogenic metabolites at their source. This system achieves coordinated reprogramming of the gut-plaque axis, resulting in durable restoration of intestinal homeostasis, attenuation of TMAO-driven immunometabolic dysfunction, and effective ISR suppression across cellular, murine, and large-animal models (Fig. 1). This work establishes a system-level ISR intervention strategy centered on the gut microbiota–immunometabolic axis and highlights engineered probiotic platforms as a generalizable paradigm for cross-organ precision therapy in complex vascular diseases.

**Fig. 1.**
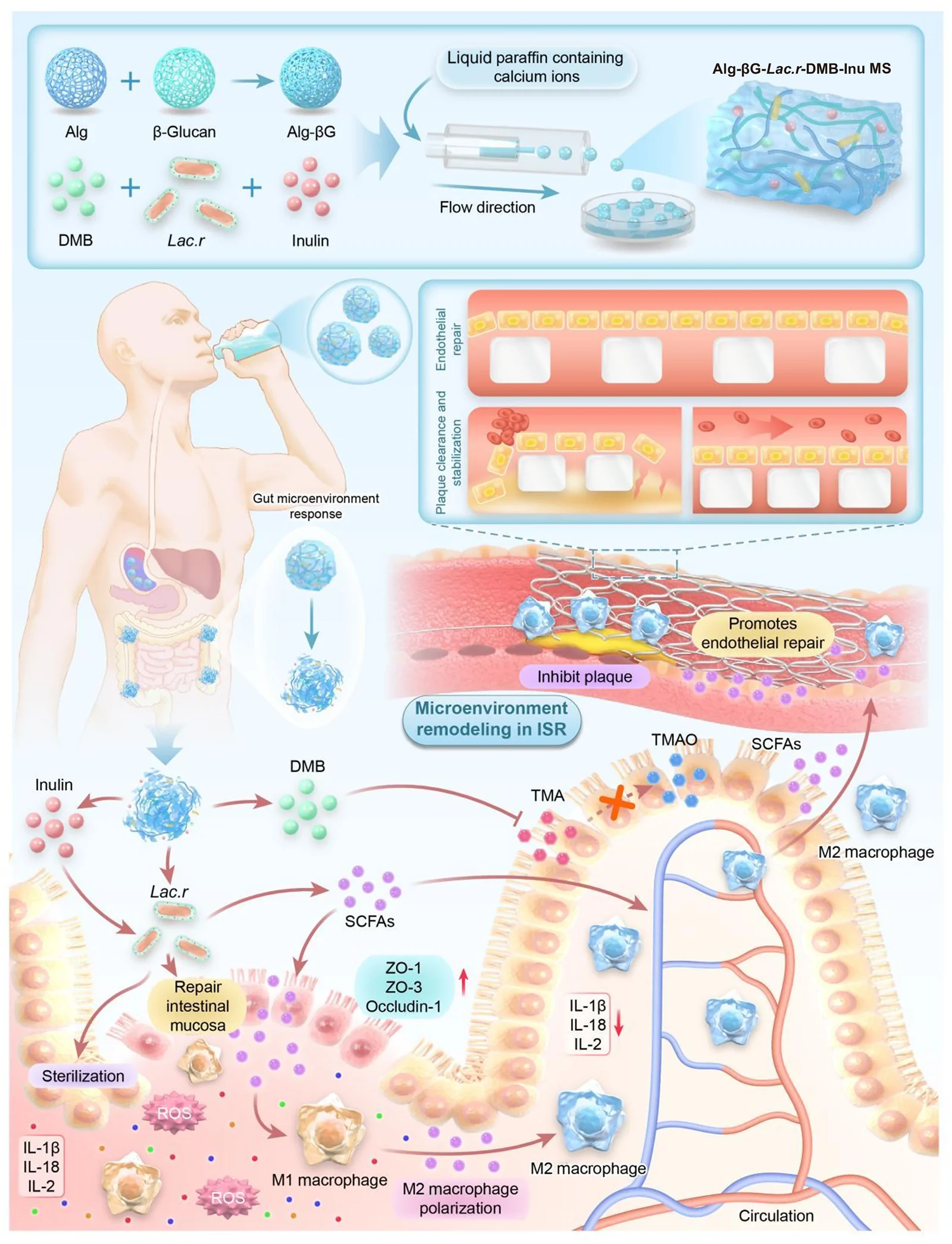
Engineered synbiotic microspheres remodel the gut-plaque axis to prevent ISR. Schematic overview illustrating the design, gastrointestinal activation, and systemic therapeutic mechanism of enzyme-responsive synbiotic microspheres (Alg-βG@*Lac.r-*DMB-Inu MS) for the prevention of ISR. Alginate (Alg) and β-glucan (βG) are coassembled via microfluidic ionotropic gelation in a calcium-containing oil phase to generate structurally stable microspheres encapsulating *Lac.r*, the microbial TMA pathway inhibitor DMB, and the prebiotic inulin. Following oral administration, the microspheres transit the gastrointestinal tract and undergo enzyme-responsive disassembly within the intestinal microenvironment, enabling site-specific release and activation of the encapsulated components. At the gut level, released *Lac.r* and inulin synergistically enhance SCFA production, reinforce intestinal barrier integrity through up-regulation of tight junction proteins (ZO-1, ZO-3, and occludin-1), suppress oxidative stress and proinflammatory cytokines [interleukin-1β (IL-1β), IL-18, and IL-2], and promote macrophage polarization toward an anti-inflammatory M2 phenotype. In parallel, DMB suppresses microbial TMA generation, thereby reducing systemic trimethylamine *N*-oxide (TMAO) levels. Collectively, coordinated modulation of microbial metabolism, immune responses, and barrier function restores immunometabolic homeostasis along the gut-plaque axis, leading to reduced plaque inflammation, enhanced endothelial repair, attenuation of neointimal hyperplasia, and stabilization of the stented vascular microenvironment, ultimately preventing ISR. Created in Adobe Illustrator 2022.

## RESULTS AND DISCUSSION

### Microbial dysbiosis–driven activation of the gut-plaque axis accelerates ISR progression

The intestinal microbiota and gut-plaque inflammatory axis of patients with ISR and healthy controls (Ctrl) were compared to determine whether gut microbial dysbiosis contributes to the pathological progression of ISR. Coronary CTA confirmed pronounced in-stent luminal narrowing in patients with ISR (Fig. 2A). Fecal 16*S* ribosomal RNA (rRNA) sequencing (*n* = 20 per group) revealed a substantial reduction in α-diversity (Wilcoxon test, *P* < 0.05) and a robust separation in β-diversity (PERMANOVA, *P* < 0.01), indicating considerable remodeling of microbial richness and community structure (Fig. 2, B to D). Multidimensional ecological assessments—including pangenome expansion, rarefaction modeling, community dissimilarity, community typing, and dysbiosis indices—consistently demonstrated a globally unstable and imbalanced microbial ecosystem in ISR (figs. S1 to S6).

**Fig. 2.**
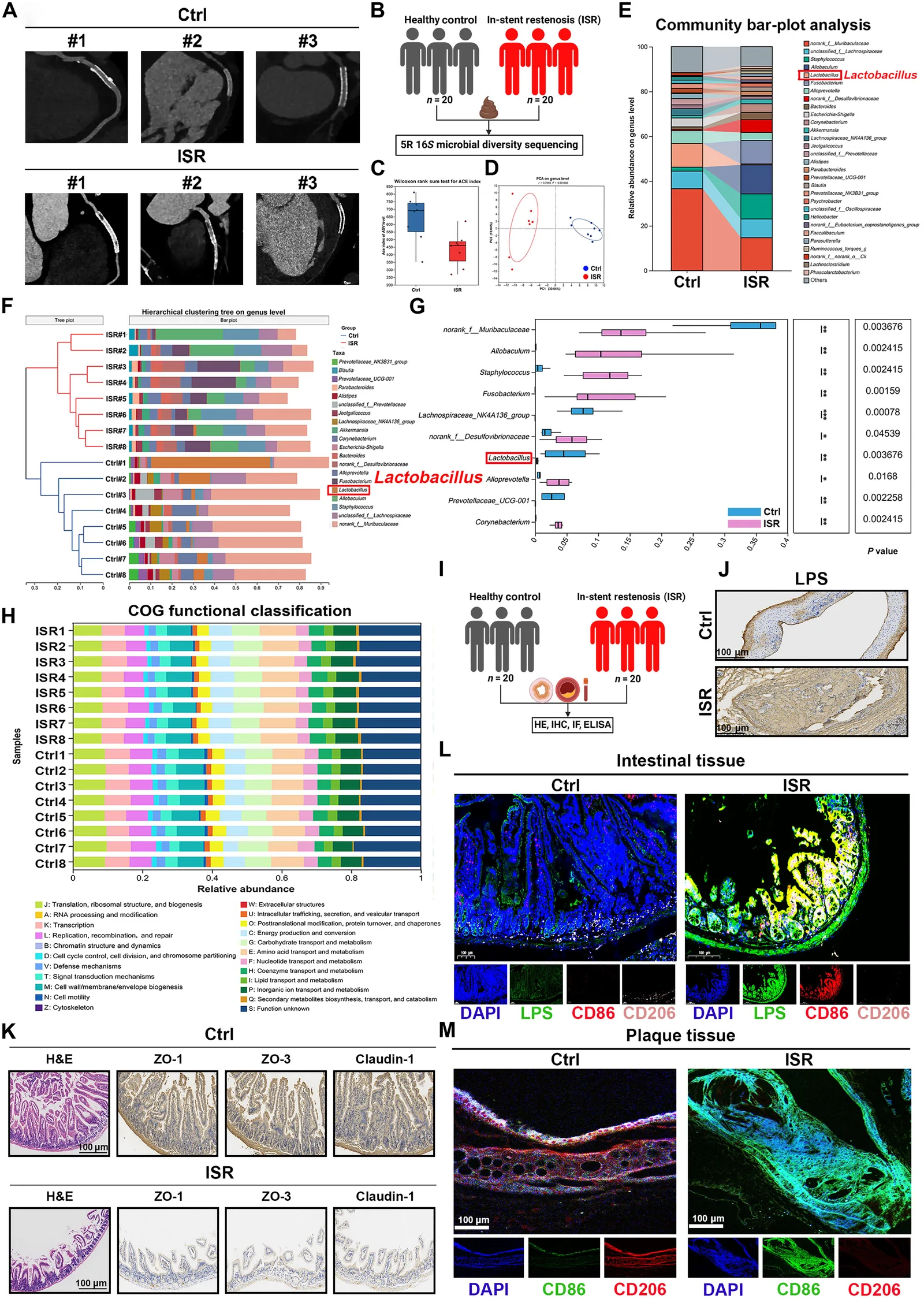
Gut microbiota dysbiosis and intestinal-plaque immune disturbance in ISR. (**A**) Representative coronary computed tomography angiographs of healthy controls (Ctrl) and patients with ISR showing in-stent lumen morphology. (**B**) Schematic illustration of fecal sample collection from Ctrl (*n* = 20) and patients with ISR (*n* = 20) for 5R 16*S* rRNA microbial diversity sequencing. Created in BioRender. Shen, L. (2026) https://BioRender.com/zrsx2if. (**C** and **D**) α-Diversity (Wilcoxon rank sum test) and β-diversity (principal coordinate analysis) analyses for evaluating microbial richness and community clustering. (**E** and **F**) Community bar plot and hierarchical clustering analyses at the genus level for microbial composition profiling. (**G**) Linear discriminant analysis effect size (LEfSe) for identifying significantly enriched bacterial taxa between groups (*P* < 0.05). (**H**) Cluster of Orthologous Groups (COG) functional classification of metagenomic annotations for evaluating microbial functional diversity. (**I**) Schematic illustration of hematoxylin and eosin (H&E), immunohistochemical (IHC), and immunofluorescence (IF) assays on pathological tissue samples from Ctrl and patients with ISR. Created in BioRender. Shen, L. (2026) https://BioRender.com/kox4smf. (**J**) IHC staining of plaque tissues for lipopolysaccharide (LPS) detection. (**K**) H&E staining and IHC analysis of tight junction proteins (ZO-1, ZO-3, and Claudin-1) in intestinal tissues. (**L**) IF staining of intestinal tissues showing 4′,6-diamidino-2-phenylindole (DAPI; blue), LPS (green), CD86 (red, M1 marker), and CD206 (pink, M2 marker). (**M**) IF staining of stented plaque tissues showing CD86 (red, M1 marker) and CD206 (pink, M2 marker). Data are presented as means ± SEM across three biologically independent samples. Significance: *P < 0.05; **P < 0.01; ***P < 0.001.

At the compositional level, patients with ISR exhibited a characteristic shift from probiotic depletion to proinflammatory expansion (Fig. 2, E and F). *Lac.r* and *Lactobacillus*-lineage taxa (e.g., *Lactobacillus* and *Limosilactobacillus*) were markedly reduced (FDR < 0.05), reflecting a loss of key commensals essential for barrier maintenance and anti-inflammatory homeostasis. In contrast, opportunistic and inflammation-associated genera—including *Prevotella*, *Desulfovibrio*, *Ruminococcus gnavus*, *Streptococcus*, *Staphylococcus*, and *Escherichia-Shigella*—were significantly enriched in ISR (FDR < 0.05). Linear discriminant analysis effect size (LEfSe) and differential abundance analyses confirmed these ISR-enriched inflammatory taxa (Fig. 2G). Supplementary microbial network analyses revealed reduced microbial connectivity, weakened network topology, and impaired ecological resilience, reinforcing the proinflammatory vulnerability of the ISR microbiome (figs. S7 to S10). Cluster of Orthologous Groups (COG) functional annotation demonstrated extensive metabolic reprogramming, particularly in amino acid metabolism, membrane transport, and stress-response pathways, highlighting the establishment of a proinflammatory metabolic niche (Fig. 2H and fig. S11).

ISR plaques showed substantial LPS deposition at the host tissue level (Fig. 2, I and J). Intestinal tissues exhibited marked epithelial barrier disruption, including significant down-regulation of ZO-1, ZO-3, and Claudin-1 (all *P* < 0.05) and prominent villus injury (Fig. 2K). Immunofluorescence analyses revealed robust mucosal LPS accumulation accompanied by enhanced infiltration of M1-polarized proinflammatory (CD86^+^) and reduced M2-polarized reparative macrophages (CD206^+^), indicative of a shift toward M1-skewed macrophage polarization within the intestinal mucosa (Fig. 2L). Notably, a highly concordant M1-dominant macrophage polarization phenotype was observed within ISR plaques, suggesting synchronized activation of inflammatory signaling along the gut-plaque axis (Fig. 2M). Enzyme-linked immunosorbent assays (ELISAs) further demonstrated elevated levels of TMA(O), interleukin-1β (IL-1β), IL-18, and tumor necrosis factor–α (TNF-α) across intestinal tissues, plasma, and plaques (*P* < 0.05), supporting translocation of gut-derived endotoxins and their systemic and local immunomodulatory effects (fig. S12).

ISR has traditionally been attributed to lesion-centered processes, including delayed endothelial repair, smooth muscle cell proliferation, and localized inflammatory amplification; however, such mechanisms are increasingly recognized as modulated by upstream systemic determinants rather than operating as an isolated vascular loop (*24*–*26*). Building on the clinical and tissue-level observations discussed, the integrated profiling supports a system-level reinterpretation of ISR pathogenesis: Gut microbial dysbiosis, epithelial barrier impairment, endotoxin leakage, and plaque immune polarization emerge as tightly interconnected components of a coordinated gut-immune-vascular continuum. Previous studies have detected microbial DNA or microbiota-derived metabolites within atherosclerotic lesions and demonstrated that experimental manipulation of the gut microbiome can influence arterial inflammation and neointimal remodeling in injury models (*26*–*28*). However, whether such gut-derived signals actively contribute to ISR progression in human disease has remained unresolved. Through demonstrating concordant alterations spanning intestinal microbial ecology, barrier integrity, circulating inflammatory mediators, and macrophage polarization within restenotic plaques, these data provide clinical evidence that aberrant activation of the gut-plaque axis functions as an upstream pathological driver of ISR, rather than a secondary or incidental byproduct of vascular injury.

### Design and fabrication of multifunctional Alg-βG-*Lac.r*-DMB-Inu MS for restoring gut-plaque axis homeostasis

A microfluidic ionotropic–gelation strategy was used to construct multifunctional Alg-βG-*Lac.r*-DMB-Inu MS capable of coencapsulating probiotics, metabolic inhibitors, and prebiotics. A premixed Alg/β-glucan matrix containing DMB, *Lac.r*, and inulin was injected into a calcium-rich liquid paraffin phase through a microfluidic nozzle, enabling instantaneous cross-linking and the formation of monodisperse microspheres (fig. S13). Optical imaging of Alg MS, Alg-DMB MS, Alg-*Lac.r* MS, Alg-βG-Inu MS, and the composite Alg-βG-*Lac.r*-DMB-Inu MS revealed uniformly sized spherical microspheres with well-defined structural boundaries, demonstrating the robustness and cross-formulation compatibility of the microfluidic fabrication process (Fig. 3A and fig. S14A). Consistent with these observations, statistical analysis of more than 100 individual microspheres revealed a narrow size distribution centered at ∼47 μm, providing quantitative evidence for the excellent monodispersity and batch-to-batch consistency of the engineered microspheres (fig. S14B). The incorporation of β-glucan generated a more compact and textured shell morphology, suggesting the formation of a denser polymer network with improved structural stability. These morphological changes were further confirmed by scanning electron microscopy (SEM; Fig. 3B). DMB- or *Lac.r*-loaded Alg particles displayed smooth surfaces; the full composite microspheres exhibited a roughened, porous outer layer consistent with β-glucan–reinforced network formation. Elemental mapping demonstrated homogeneous distribution of C, N, Na, S, P, Ca, and O throughout the microsphere interior, indicating uniform embedding of bacterial, small molecule, and polysaccharide components within the Alg-βG matrix (Fig. 3C).

**Fig. 3.**
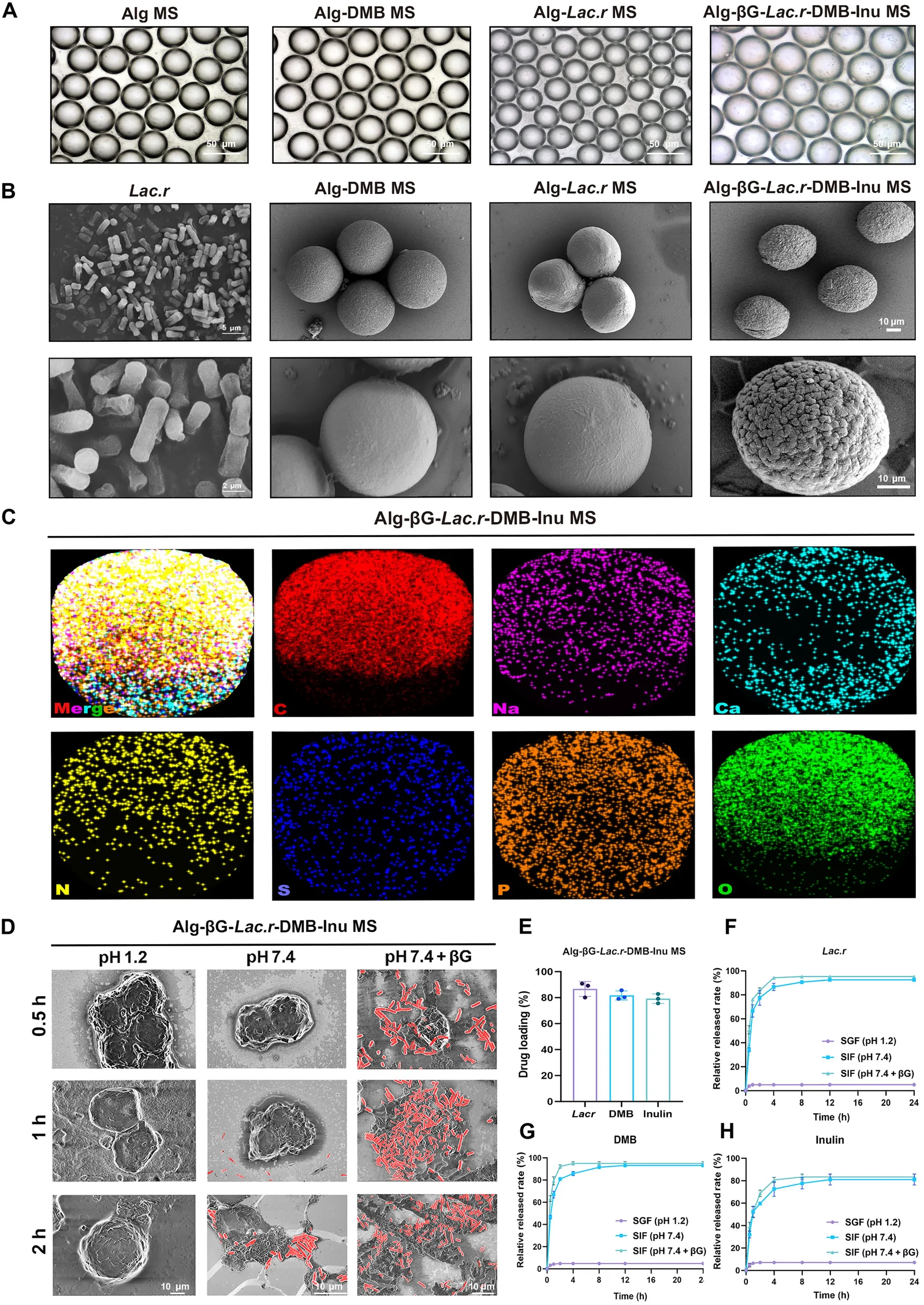
Characterization of Alg-βG-*Lac.r*-DMB-Inu microspheres. (**A**) Optical images of different microspheres: Alg, Alg-DMB, Alg-*Lac.r*, and Alg-βG-*Lac.r*-DMB-Inu. Scale bars, 100 μm. (**B**) Scanning electron micrographs of the microspheres and *Lac.r*. Scale bars, 5 μm. (**C**) Elemental mapping of the Alg-βG-*Lac.r*-DMB-Inu microspheres, showing the distribution of carbon (C, red), nitrogen (N, yellow), sodium (Na, purple), sulfur (S, blue), phosphorus (P, orange), calcium (Ca, cyan), and oxygen (O, green) within the microspheres. (**D**) Scanning electron micrographs of Alg-βG-*Lac.r*-DMB-Inu MS incubated at different pH values (1.2, 7.4, and 7.4 + βG) for 0.5, 1, and 2 hours. (**E**) Drug loading efficiency of the Alg-βG-*Lac.r*-DMB-Inu MS for *Lac.r*, DMB, and inulin, measured at pH 7.4. (**F** to **H**) Release profiles of *Lac.r* (F), DMB (G), and inulin (H) from the Alg-βG-*Lac.r*-DMB-Inu MS in simulated intestinal fluid (SIF) at pH 7.4, 1.2, and 7.4 + βG. Data are presented as means ± SEM across three biologically independent samples. h, hours.

Microspheres were incubated under acidic and intestinal conditions (pH 1.2, 7.4, and 7.4 supplemented with β-glucanase) to assess gastrointestinal responsiveness. The microspheres remained structurally intact at pH 1.2. Gradual shell loosening and controlled disintegration occurred in the β-glucanase–containing medium, accompanied by the release of encapsulated *Lac.r* (Fig. 3D). Consistent with this structural robustness, the composite microspheres exhibited high loading efficiencies for *Lac.r*, DMB, and inulin at physiological pH, indicating efficient multicomponent incorporation within the Alg-βG network (Fig. 3E). The release profiles of *Lac.r*, DMB, and inulin showed minimal liberation under acidic conditions and sustained release at pH 7.4, with further acceleration in β-glucanase–enriched medium (Fig. 3, F to H).

To validate these physicochemical properties under biological challenge, free *Lac.r* and Alg-βG-*Lac.r-*DMB-Inu MS were exposed to simulated gastric fluid (SGF), simulated intestinal fluid (SIF), and multistage SGF-BA-SIF gastrointestinal transit. Free *Lac.r* exhibited rapid viability loss under acidic conditions; encapsulated *Lac.r* remained substantially recoverable throughout the same exposure periods (fig. S15). Under bile acids, oxidative stress, and the full continuous SGF-BA-SIF sequence, free *Lac.r* exhibited minimal survival; encapsulated bacteria retained marked colony-forming ability, indicating that the microsphere matrix provides robust biological protection during compounded gastrointestinal stress (fig. S16).

To further determine whether encapsulation preserves the metabolic functionality of *Lac.r* following gastrointestinal exposure, SCFA production by released bacteria was quantified under identical culture conditions. Following simulated gastrointestinal treatment, encapsulated *Lac.r* largely retained the production of acetic acid (AA), propionic acid (PA), and butyric acid (BA), whereas free bacteria exhibited a marked reduction in SCFA production (fig. S17, A to C). Consistent with these in vitro findings, treatment with the engineered synbiotic microspheres significantly increased circulating SCFA concentrations in vivo (fig. S17D), demonstrating that the encapsulation strategy preserves the metabolic functionality of *Lac.r* while enhancing its functional performance after gastrointestinal transit.

Beyond gastrointestinal protection, we further evaluated the long-term storage stability of Alg-βG-*Lac.r*-DMB-Inu MS to assess the translational robustness of the engineered synbiotic platform. The microspheres retained structural integrity during refrigerated storage (4°C) for up to 6 months, whereas gradual structural deterioration was observed at room temperature (24°C) (fig. S18A). Live/dead quantification demonstrated sustained bacterial viability throughout refrigerated storage but a progressive decline after prolonged storage at 24°C (fig. S18, B and C). Consistently, live/dead fluorescence imaging confirmed predominantly viable bacteria under refrigerated conditions, whereas room-temperature storage resulted in a time-dependent increase in bacterial death (fig. S18, D and E). Together, these findings demonstrate that the encapsulation strategy enables long-term preservation of probiotic viability under refrigerated conditions, further supporting the stability and translational potential of the engineered synbiotic microsphere platform.

A time-resolved imaging strategy was established to evaluate intestinal retention in vivo in which animals received a single oral gavage followed by ex vivo IVIS analysis of intestinal tissues collected at 2, 4, 24, 48, and 96 hours (Fig. 4A). IVIS imaging revealed that free *Lac.r* exhibited weak and rapidly diminishing fluorescence signals after administration, indicating minimal gastrointestinal persistence. In contrast, Alg-βG-*Lac.r*-DMB-Inu MS generated strong, spatially extensive fluorescence signals throughout the gastrointestinal tract from 2 to 48 hours, which remained detectable at 96 hours albeit with partial signal attenuation (Fig. 4B). Collectively, these findings demonstrate that microsphere encapsulation enhances and prolongs probiotic retention and luminal distribution within the gut in vivo.

**Fig. 4.**
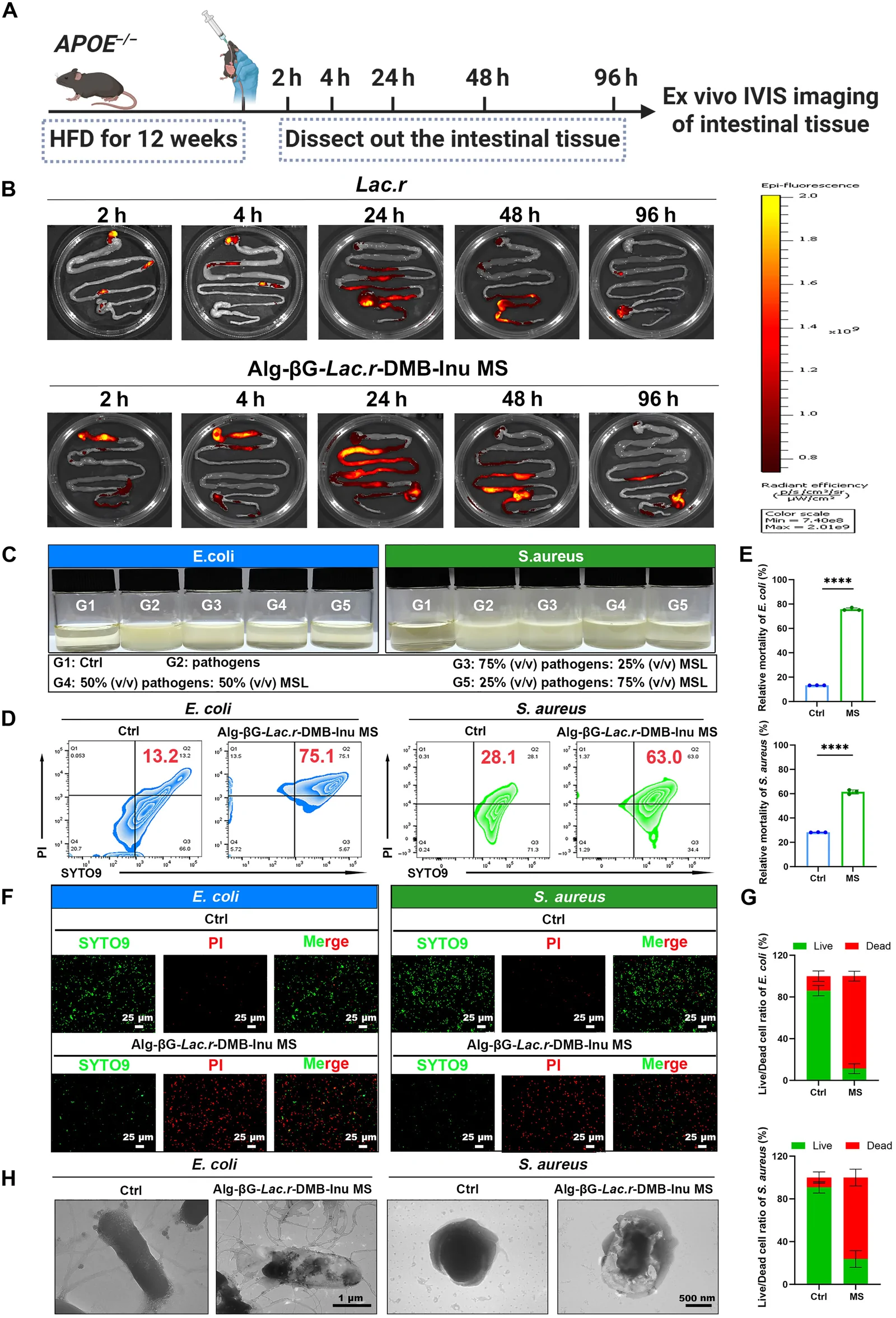
Gut retention and antibacterial effects of Alg-βG-*Lac.r*-DMB-Inu microspheres. (**A**) Mice fed a high-fat diet (HFD) for 12 weeks received oral administration. Gastrointestinal tissues were collected at defined time points (2 to 96 hours) for ex vivo IVIS imaging. Created in Biorender. Shen, L. (2026) https://BioRender.com/rhn2wyg. (**B**) IVIS imaging for ex vivo monitoring of gut retention of *Lac.r* and Alg-βG-*Lac.r*-DMB-Inu microspheres at 2, 4, 24, 48, and 96 hours. h, hours. (**C**) Representative visual assessments of *E. coli* and *S. aureus* cultured under different medium compositions (LB, LB-MSL, and mixed LB-pathogens/LB-MSL systems). G1, Ctrl; G2, pathogens; G3, 75% (v/v) pathogens + 25% (v/v) MSL; G4, 50% (v/v) pathogens + 50% (v/v) MSL; and G5, 25% (v/v) pathogens + 75% (v/v) MSL. (**D**) Flow cytometry analysis of *E. coli* and *S. aureus* viability after treatment; SYTO9^+^ cells indicate live bacteria and PI^+^ cells indicate dead bacteria. (**E**) Flow cytometric quantification of live (green) and dead (red) bacterial populations under different treatments. (**F**) Confocal fluorescence images of *E. coli* and *S. aureus* stained with SYTO9 (green, live) and PI (red, dead) after treatment with Alg-βG-*Lac.r*-DMB-Inu microspheres. (**G**) Quantitative analysis of bacterial viability (live/dead assay). (**H**) Representative transmission electron micrograph of *E. coli* and *S. aureus* treated with Alg-βG-*Lac.r*-DMB-Inu MS. Data are presented as means ± SEM across three biologically independent samples. Significance: ****P < 0.0001.

To assess antibacterial activity, the effects of microsphere leachates (MSLs; defined as the soluble fraction released from microspheres after incubation in culture medium) were evaluated on *Escherichia coli* and *Staphylococcus aureus* (fig. S19). The biocompatibility of the microsphere formulation was assessed in parallel using representative mammalian cell types. Fluorescence-based live/dead staining revealed no detectable cytotoxicity in RAW264.7 macrophages, MOVAS smooth muscle cells, or human umbilical cord endothelial cells (HUVECs) following 24-hour incubation with Alg-βG-*Lac.r-*DMB-Inu MS; cell viability was comparable to that of the control group (fig. S20). This indicates that the antibacterial activity of the MSLs is achieved without inducing nonspecific cytotoxicity toward host cells, supporting a selective and biologically compatible antimicrobial profile. Consistent with this observation, visible bacterial growth was progressively suppressed in mixed LB/LB-MSL cultures with increasing proportions of MSLs (Fig. 4C). To further identify the source of this antibacterial activity, agar diffusion assays were performed using Reuterin, Reuterin supplemented with inulin, Alg-βG MS, and Alg-βG-*Lac.r* MS. Whereas Alg-βG MS alone exhibited negligible antibacterial activity, both Reuterin and Alg-βG-*Lac.r* MS generated pronounced inhibition zones (fig. S21), indicating that the antibacterial activity of the MSLs is predominantly attributable to *Lac.r*-derived antimicrobial metabolites rather than the carrier matrix itself.

Flow cytometry revealed a substantial increase in PI^+^ dead bacteria following MSL treatment and quantitative analysis confirmed significantly elevated bacterial mortality compared with controls (Fig. 4, D and E). Confocal imaging corroborated these findings: MSL exposure shifted both *E. coli* and *S. aureus* from predominantly SYTO9^+^ live cells to a higher proportion of PI^+^ dead cells (Fig. 4F). Consistent reductions in live bacterial populations were also observed by flow cytometric quantification (Fig. 4G). Transmission electron microscopy (TEM) revealed extensive ultrastructural damage in pathogenic bacteria following MSL treatment, including cell wall disruption, membrane collapse, and severe morphological disintegration, consistent with the potent antibacterial activity of the engineered synbiotic platform (Fig. 4H). To further define the intestinal distribution of the released probiotics beyond the spatial resolution of IVIS imaging, fluorescence in situ hybridization (FISH) was performed across different intestinal segments. *Lac.r* was detected throughout the gastrointestinal tract, with progressively stronger fluorescence signals from the duodenum to the colon, indicating preferential retention within the distal intestine (fig. S22, A and C). Longitudinal fecal monitoring further demonstrated that encapsulation markedly prolonged probiotic persistence compared with free *Lac.r*. During the administration period, fecal bacterial abundance remained consistently higher in mice receiving Alg-βG-*Lac.r*-DMB-Inu MS, and, after treatment withdrawal, encapsulated *Lac.r* exhibited substantially prolonged persistence, whereas free bacteria declined rapidly toward baseline levels (fig. S22B). Together, these findings demonstrate that the engineered synbiotic microspheres not only protect *Lac.r* during gastrointestinal transit but also promote its sustained intestinal retention and persistence in vivo.

From an engineering and translational perspective, the key limitation of current probiotic interventions does not lie in strain selection, but in insufficient viable loading, unstable intestinal retention, and ineffective coupling with host immunometabolic networks (*29*, *30*). Conventional oral formulations rely on passive survival and stochastic release, resulting in unpredictable efficacy in dynamic gastrointestinal environments, particularly for systemically driven diseases such as ISR (*23*, *31*). This enzyme-responsive composite probiotic microsphere integrating structural protection, intestine-specific activation, and metabolic coregulation within a single platform was engineered to address these challenges. The Ca^2+^–cross-linked alginate–β-glucan shell remains stable under gastric conditions but undergoes selective disassembly in response to intestinal β-d-glucosidase, enabling site-specific release (*32*, *33*). Concurrent delivery of inulin and the metabolic inhibitor DMB further promotes probiotic colonization while suppressing TMAO-associated pathways, establishing a closed functional loop encompassing delivery, activation, colonization, and downstream immunometabolic modulation (*22*, *23*, *34*). This strategy shifts probiotic therapy from empirical supplementation toward a controllable, mechanistically tractable engineering intervention for remodeling the gut-plaque axis and suppressing ISR progression.

### Remodeling of the gut-plaque axis by Alg-βG-*Lac.r*-DMB-Inu MS restores microbial-immune homeostasis and suppresses ISR

The activity of the Alg-βG-*Lac.r*-DMB-Inu MS across the major ISR-relevant cell types (macrophage-derived foam cells, smooth muscle–derived foam cells, and inflamed endothelial cells) was evaluated to comprehensively characterize their cellular therapeutic profile. In macrophage-derived foam cells, the composite microspheres produced the most pronounced attenuation of intracellular reactive oxygen species (ROS) and a marked reduction in lipid droplet content, reflecting an improved cholesterol-processing phenotype (Fig. 5, A to D). In smooth muscle–derived foam cells, Alg-βG-*Lac.r-*DMB-Inu MS similarly reduced oxidative stress and stabilized cytoskeletal architecture, evidenced by decreased F-actin stress fiber formation indicating effective suppression of inflammation-driven migratory activation (Fig. 5, E and F; and figs. S23 and S24). Beyond foam cell modulation, the microspheres exerted marked protective effects on endothelial cells. In LPS-challenged HUVECs, Alg-βG-*Lac.r*-DMB-Inu MS significantly reduced intracellular ROS accumulation and lipid deposition while promoting endothelial wound closure, indicating improved endothelial function and repair capacity (Fig. 5, G and H; and fig. S25, A to D). To distinguish endothelial migration from proliferation, HUVECs were pretreated with mitomycin C, and 5 μg ml^−1^ was identified as the optimal concentration for proliferation inhibition without overt cytotoxicity (fig. S25, E and I). Under these conditions, the composite microspheres continued to significantly accelerate wound closure, confirming that the enhanced repair was primarily attributable to increased endothelial migration rather than cell proliferation (fig. S25, F, G, J, and K). In parallel, EdU incorporation assays demonstrated that Alg-βG-*Lac.r-*DMB-Inu MS significantly suppressed MOVAS proliferation (fig. S25, H and L), further supporting their ability to simultaneously promote physiological endothelial repair while limiting pathological smooth muscle cell expansion.

**Fig. 5.**
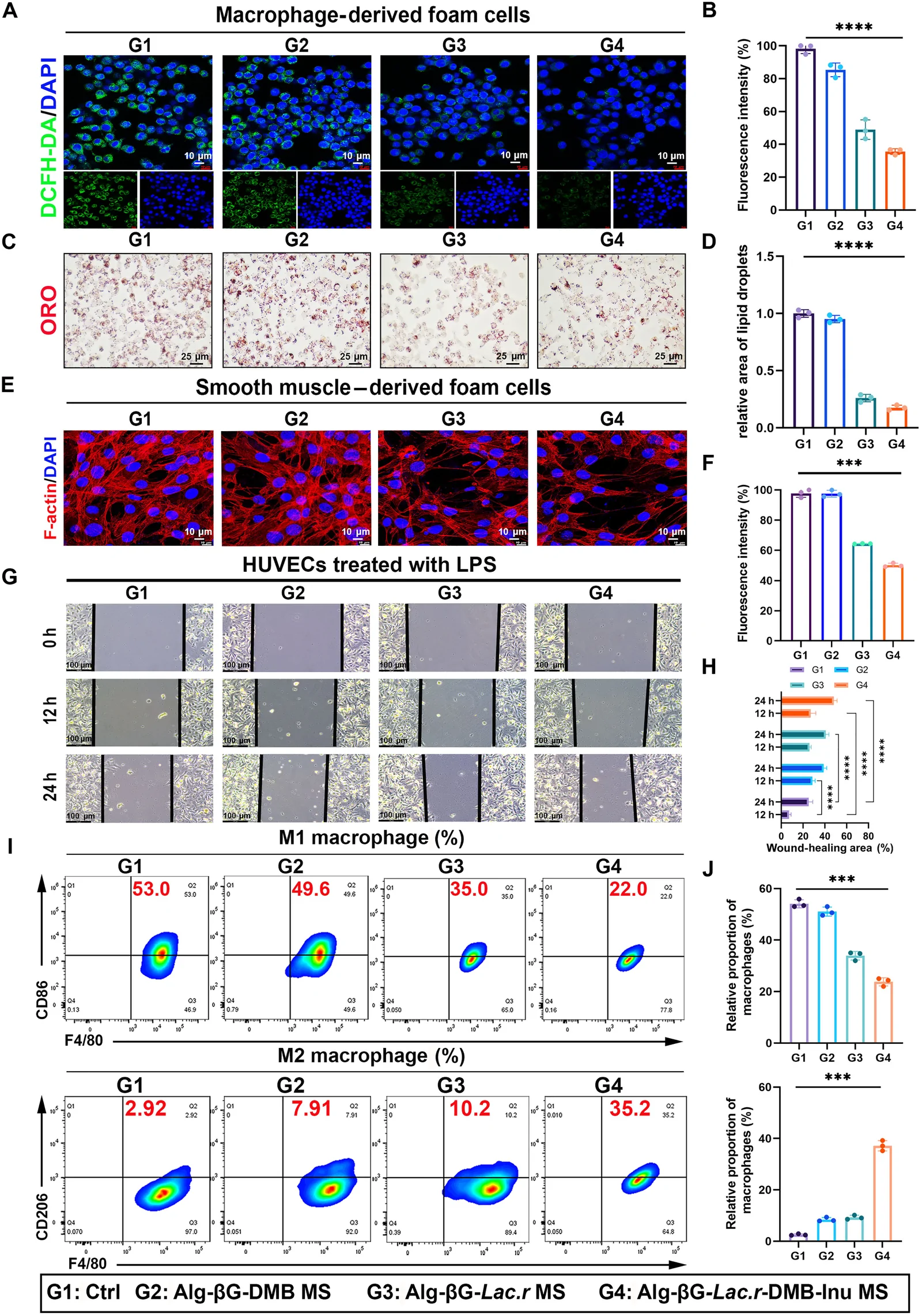
In vitro suppression of ISR by Alg-βG-*Lac.r*-DMB-Inu MS. (**A**) Representative confocal images of macrophage-derived foam cells under different treatments (G1, Ctrl; G2, Alg-βG-DMB; G3, Alg-βG-*Lac.r*; G4, Alg-βG-*Lac.r*-DMB-Inu), showing 2′,7′-dichlorodihydrofluorescein diacetate (DCFH-DA) fluorescence (green, reactive oxygen species, ROS) and DAPI (blue, nuclei). (**B**) Quantification of ROS fluorescence intensity in (A). (**C**) ORO staining of macrophage-derived foam cells following different treatments. (**D**) Quantification of the relative area of lipid droplets in (C). (**E**) Representative confocal fluorescence images of smooth muscle–derived foam cells, with F-actin staining (red) and DAPI (blue, nuclei). (**F**) Quantification of fluorescence intensity in (E). (**G**) Representative wound healing images of LPS-induced HUVECs after treatment with different groups at 0, 12, and 24 hours. (**H**) Quantification of wound healing area in (G). (**I**) Flow cytometry analysis of M1 (CD86) and M2 (CD206) macrophage polarization markers in four treatment groups (G1, Ctrl; G2, Alg-βG-DMB; G3, Alg-βG-*Lac.r*; and G4, Alg-βG-*Lac.r*-DMB-Inu). (**J**) Statistical histograms of the relative proportion of M1 and M2 macrophages in each group. Data are presented as means ± SEM across three biologically independent samples. Significance: ***P < 0.001; ****P < 0.0001.

To mechanistically consolidate these cellular phenotypes, multilevel analyses were next conducted in foam cells derived from distinct cellular origins. Transcriptional profiling in RAW264.7-derived foam cells revealed coordinated regulation of genes associated with inflammatory signaling, macrophage polarization, and cholesterol efflux following microsphere treatment (fig. S26). Analogous transcriptional modulation was observed in MOVAS-derived foam cells, indicating that these regulatory effects are conserved across macrophage- and smooth muscle–derived foam cell populations (fig. S27). Western blot analyses confirmed concordant regulation of key cholesterol efflux transporters (ABCA1 and ABCG1) in both foam cell types, together with aligned modulation of macrophage polarization markers (CD206 and CD86) at the protein level (fig. S28). These findings establish a coherent immunometabolic regulatory framework operating across multiple cellular lineages and molecular layers, providing mechanistic support for the observed endothelial protection, immune reprogramming, and in vivo therapeutic efficacy.

The ability of Alg-βG-*Lac.r*-DMB-Inu MS to correct gut-plaque axis disturbances in high-fat diet (HFD)–fed ApoE^−/−^ mice was next evaluated (Fig. 6A). Across the six treatment groups (G1, Ctrl; G2, HFD; G3, HFD + FMT; G4, HFD + Alg-βG-DMB MS; G5, HFD + Alg-βG-*Lac.r* MS; and G6, HFD + Alg-βG-*Lac.r*-DMB-Inu MS), the outcomes exhibited clear, internally coherent treatment-dependent trends. This pattern was reflected both in the phenotypic appearance of the mice at the study endpoint and in their longitudinal body weight trajectories (Fig. 6, B and C).

**Fig. 6.**
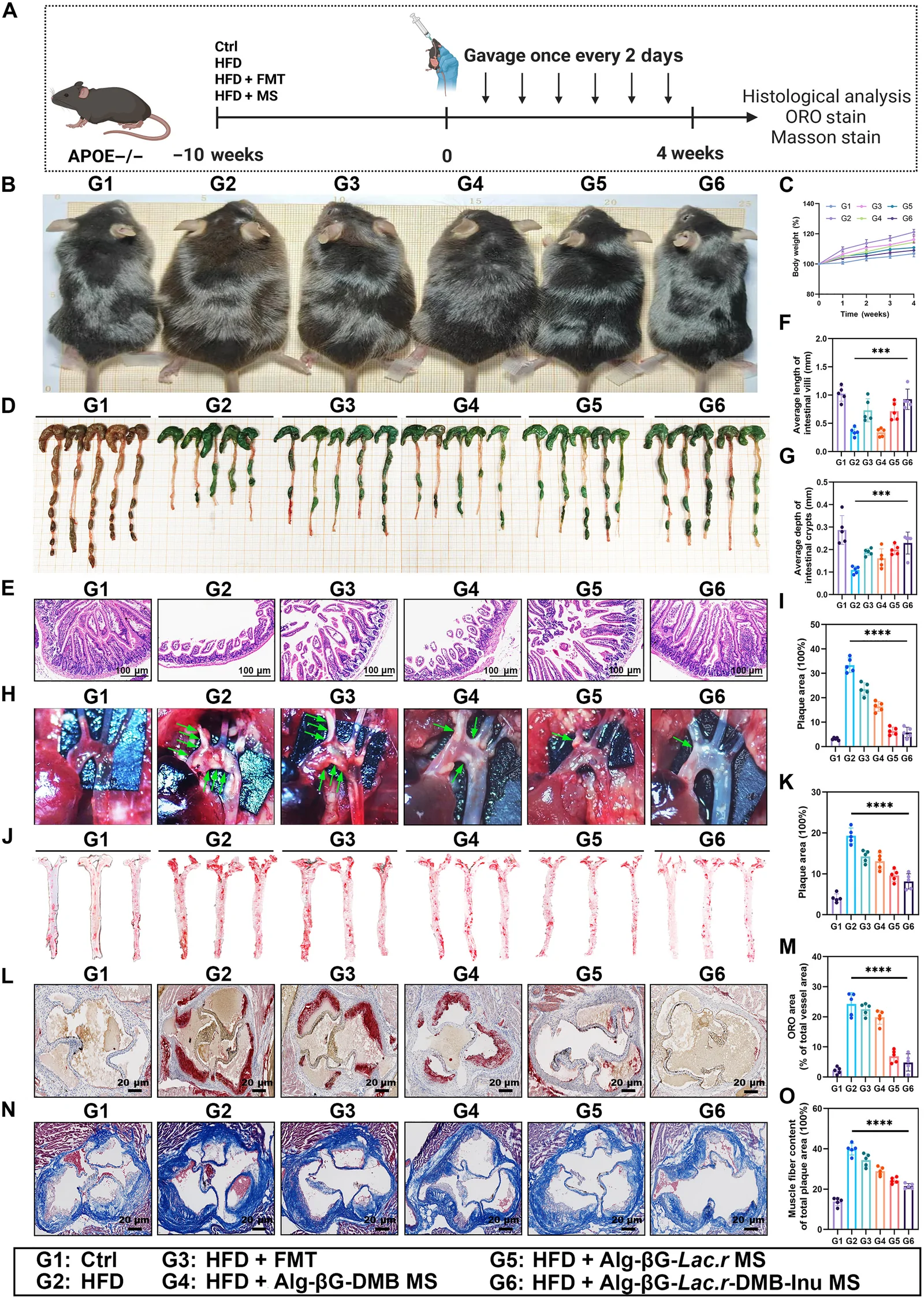
In vivo modulation of the gut-plaque axis by Alg-βG-*Lac.r*-DMB-Inu MS. (**A**) Experimental timeline of treatment administration to *ApoE*^−/−^ mice, including Ctrl, HFD, HFD + FMT, and HFD + MS groups, with gavage every other day for 4 weeks. Created in BioRender. Shen, L. (2026) https://BioRender.com/3k8bxnt. (**B**) Representative images of *ApoE*^−/−^ mice after 4 weeks of treatment: G1, Ctrl; G2, HFD; G3, HFD + FMT; G4, HFD + Alg-βG-DMB MS; G5, HFD + Alg-βG-*Lac.r* MS; and G6, HFD + Alg-βG-*Lac.r*-DMB-Inu MS. (**C**) Body weight changes of mice in different treatment groups over the 4-week period. (**D**) Representative images of full-length intestinal samples from different treatment groups. (**E**) Representative H&E staining images of intestinal tissues from different treatment groups. (**F**) Statistical histograms of intestinal villus length from different groups in (E). (**G**) Statistical histogram of average intestinal crypt depth from different groups in (E). (**H**) Stereomicroscopic images of aortic plaques in different treatment groups; plaque regions are indicated by green arrows. (**I**) Statistical histogram of aortic plaque area in (H). (**J**) Representative Oil Red O staining of ex vivo aortic plaques. (**K**) Statistical histogram of Oil Red O staining plaque area in ex vivo aortic plaques from (J). (**L**) Representative Oil Red O staining images of aortic root plaques. (**M**) Statistical histogram of Oil Red O staining plaque area in the aortic root from (L). (**N**) Representative Masson’s trichrome staining of aortic root plaques. (**O**) Statistical histogram of muscle fiber content in aortic root plaques from (N). Data are presented as means ± SEM across three biologically independent samples. Significance: ***P < 0.001; ****P < 0.0001.

HFD markedly shortened colon length at the intestinal level, whereas all three microsphere formulations partially restored intestinal morphology. This trend was evident from gross intestinal images and the corresponding quantitative bar chart showing a graded recovery of colon length with the most pronounced improvement in the composite microsphere group (Fig. 6D and fig. S29A). Hematoxylin and eosin (H&E) staining corroborated these findings, demonstrating progressive alleviation of HFD-induced villous damage and crypt atrophy in the DMB and *Lac.r* MS–treated groups and the most pronounced architectural restoration in the composite formulation within the small intestine (Fig. 6, E to G). Consistent histomorphological improvements were also observed in the colon, as shown in the corresponding H&E-stained sections, indicating that microsphere-mediated protection extends beyond the small intestine and promotes coordinated structural preservation across the intestinal tract (fig. S29B). To further characterize intestinal barrier restoration, we examined the expression of multiple tight-junction proteins in both the colon and small intestine. Immunofluorescence staining demonstrated that Alg-βG-*Lac.r-*DMB-Inu MS markedly restored ZO-1 expression in colonic tissues compared with HFD controls (fig. S30, A and B). Consistently, immunohistochemical analyses revealed significantly increased expression of ZO-1, Occludin, and Claudin-1 in both the colon and small intestine following microsphere treatment (fig. S30, C to J), indicating broad restoration of epithelial tight-junction architecture throughout the intestinal tract. These findings further support the ability of the engineered synbiotic microspheres to preserve intestinal barrier integrity, complementing the histological evidence of intestinal protection observed in the ISR model.

At the vascular level, Oil Red O staining of histological aortic sections revealed pronounced plaque accumulation in HFD mice. Both FMT and single-component microspheres moderately reduced lesion burden. Alg-βG-*Lac.r-*DMB-Inu MS markedly decreased plaque area, approaching the Ctrl phenotype (Fig. 6, H to M). Metabolic profiling by filipin staining showed a parallel pattern: HFD elevated intraplaque free-cholesterol levels and all microsphere formulations attenuated cholesterol loading, with the composite microspheres producing the most pronounced reduction (fig. S31). Masson’s trichrome staining revealed a stepwise reduction in plaque fibrosis across treatments, with the composite microspheres producing the greatest decline in collagen deposition (Fig. 6, N and O).

In parallel with the attenuation of plaque burden, lipid deposition, and fibrotic remodeling, it was next investigated whether Alg-βG-*Lac.r*-DMB-Inu MS elicited coordinated modulation of systemic adaptive immunity (fig. S32). Flow cytometric analysis of splenic T cell subsets revealed that HFD induced a pronounced expansion of CD3^+^ T cells, accompanied by enrichment of proinflammatory CD4^+^ and CD8^+^ effector populations and a concomitant depletion of CD4^+^ CD25^+^ Foxp3^+^ regulatory T (T_reg_) cells. Although FMT and single-component microspheres partially mitigated these immune disturbances, Alg-βG-*Lac.r*-DMB-Inu MS most effectively restored T cell homeostasis, normalizing effector T cell proportions and robustly expanding the T_reg_ cell compartment. This immune rebalancing reflected a shift toward an immunoregulatory state rather than global immunosuppression, closely mirroring the extent of vascular and plaque-level improvements.

A central question in the field is whether gut microbiota modulation alone is sufficient to achieve durable vascular benefit (*13*, *35*). Fecal microbiota transplantation has been instrumental in establishing causal microbiome-phenotype relationships; however, its ecological complexity, donor dependency, and limited controllability constrain translational applicability (*36*–*38*). Similarly, mono-probiotic supplementation often suffers from inadequate gastrointestinal persistence and fails to suppress pathogenic metabolic pathways, and metabolic inhibition alone does not restore ecological stability or barrier function (*39*). These comparative in vivo analyses address this unresolved issue. The graded efficacy observed across FMT, single-component microspheres and the composite formulation indicates that ISR is maintained by a multilevel pathological network rather than a single dysregulated node. Durable suppression therefore requires coordinated axis-level intervention simultaneously targeting microbial ecology, metabolic output, and host barrier integrity.

In orthotopic ISR models established in rats and rabbits, Alg-βG-*Lac.r*-DMB-Inu MS exhibited robust, cross-system therapeutic efficacy reproducible across species and imaging modalities (Fig. 7A and fig. S33). In rats, longitudinal magnetic resonance imaging (MRI) demonstrated progressive lesion enlargement in controls; the composite microsphere group showed significant regression beginning on day 7 and accelerating through days 14 and 28, achieving an overall reduction of ∼60 to 70% (versus Ctrl, *P* < 0.01) (Fig. 7B). The steeper regression slope compared with single-component microspheres underscores the synergistic nature of the composite formulation in suppressing neointimal hyperplasia. Histological analyses were consistent with the imaging data, demonstrating significant reductions in plaque burden and intima-to-medium ratio (50 to 70%; *P* < 0.001) and diminished inflammatory infiltration. The composite microspheres exhibited superior anti-ISR efficacy compared with other treatment groups (Fig. 7C). Intestinal assessments revealed restored villus architecture, improved epithelial continuity, and reinforced tight junction integrity, indicating effective disruption of the pathological cascade linking epithelial barrier breakdown with endotoxin leakage and vascular inflammation.

**Fig. 7.**
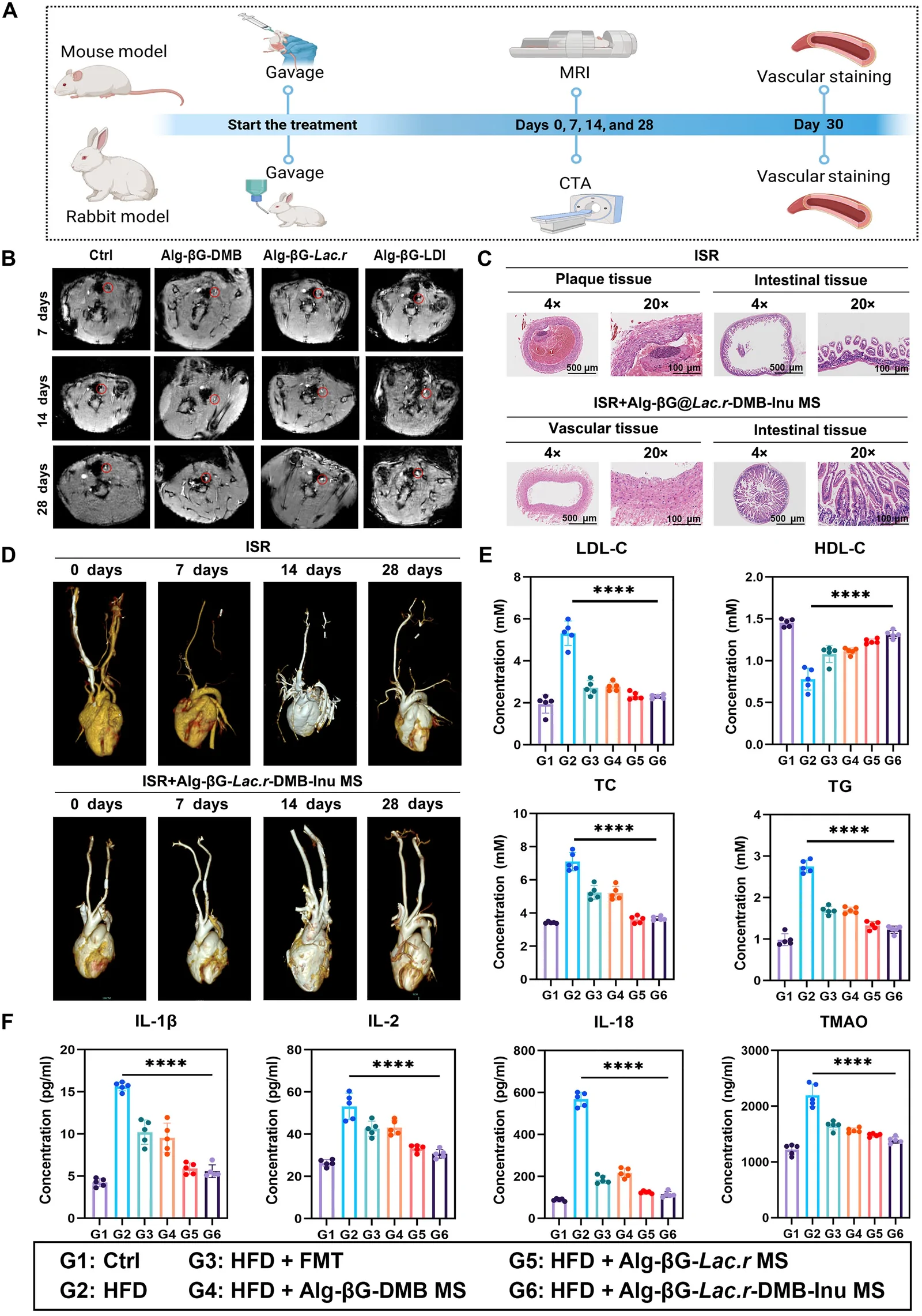
Alg-βG-*Lac.r*-DMB-Inu MS mitigate ISR in vivo by reprogramming the gut-plaque axis. (**A**) Schematic illustration of the in vivo ISR model timeline, including treatment regimen, magnetic resonance imaging (MRI)/CTA imaging, and histological evaluation. Created in BioRender. Shen, L. (2026) https://BioRender.com/tubq5bx. (**B**) Representative MRIs of ISR lesions in different treatment groups (Ctrl, Alg-βG-DMB, Alg-βG-*Lac.r*, and Alg-βG-*Lac.r*-DMB-Inu) at 7, 14, and 28 days. (**C**) Representative H&E-stained sections (4× and 20×) of plaque, vascular, and intestinal tissues from ISR models with or without Alg-βG-*Lac.r*-DMB-Inu MS treatment. (**D**) Three-dimensional CTA reconstructions of arterial structures in ISR and Alg-βG-*Lac.r*-DMB-Inu MS-treated rabbits at 0, 7, 14, and 28 days. (**E**) Serum lipid profiles (LDL-C, HDL-C, TC, and TG) measured by ELISA in different treatment groups (G1, Ctrl; G2, HFD; G3, HFD + FMT; G4, HFD + Alg-βG-DMB; G5, HFD + Alg-βG-*Lac.r*; and G6, HFD + Alg-βG-*Lac.r*-DMB-Inu). (**F**) Serum levels of inflammatory cytokines (IL-1β, IL-2, and IL-18) and metabolite TMAO measured by ELISA in different treatment groups. Data are presented as means ± SEM across three biologically independent samples. Significance: ****P < 0.0001.

In the rabbit ISR model, an anatomical scale more analogous to human disease, CTA-based three-dimensional vascular reconstruction revealed the most pronounced luminal restoration in the composite group (∼40 to 60% improvement; *P* < 0.001), signifying stenosis reversal and structural vascular remodeling (Fig. 7D). Therapeutic phenotypes captured by rat MRI and rabbit CTA exhibited strong cross-species concordance, with both imaging modalities consistently revealing substantial lesion regression and effective lumen restoration, reinforcing the translational robustness of this therapeutic strategy. In line with these imaging outcomes, metabolic profiling showed that the composite microsphere group achieved the most pronounced reductions in LDL-C, total cholesterol (TC), and triglycerides (TGs), together with a concomitant elevation in HDL-C (Fig. 7E). A persistent challenge in restenosis research is the limited translatability of single-model efficacy (*40*, *41*). The consistent therapeutic signatures observed across rat and rabbit ISR models, in addition to the convergent imaging and histological readouts, argue against a model-specific mechanism. Instead, they support a scenario in which upstream resetting of a shared gut-immune-vascular axis yields reproducible downstream vascular outcomes. This cross-species consistency strengthens the translational relevance of axis-oriented intervention and suggests that targeting systemic drivers may overcome the durability limitations of lesion-focused approaches (*42*, *43*).

Concurrently, systemic inflammatory and gut-derived markers—including IL-1β, IL-2, IL-18, and TMAO—showed the greatest declines (∼40 to 65%; all *P* < 0.0001), indicating restoration of immunometabolic homeostasis (Fig. 7F). Conversely, circulating anti-inflammatory cytokines, including IL-10 and transforming growth factor–β (TGF-β), were significantly restored following treatment with Alg-βG-*Lac.r*-DMB-Inu MS (fig. S34), further supporting the establishment of a systemic anti-inflammatory immune milieu. Microbiota-dependent TMAO has emerged as a key metabolic link between gut microbial activity and cardiovascular risk (*44*–*46*). Notably, DMB acts primarily as a structural analog of choline and preferentially inhibits microbial choline trimethylamine-lyase (CutC), thereby suppressing choline-dependent TMA production without directly targeting the distinct carnitine oxygenase (CntA/B) pathway (*23*, *47*). Previous biochemical studies further demonstrated that DMB preferentially interferes with the CutC/CutD enzymatic system while exhibiting minimal effects on microbial viability, supporting its utility as a selective microbial metabolic inhibitor rather than a broad-spectrum antimicrobial agent (*23*). This mechanistic specificity is consistent with the substantial reduction in circulating TMAO observed in the present study and supports the rationale for combining targeted CutC inhibition with ecological modulation through probiotic supplementation.

Therapeutic remodeling of the gut-plaque axis is likely driven not only by suppression of detrimental microbial metabolites but also by restoration of beneficial microbial metabolism. Among SCFAs, acetate primarily maintains intestinal barrier integrity and systemic metabolic homeostasis, whereas propionate and butyrate exert more potent immunoregulatory effects through activation of G protein–coupled receptors (GPR41, GPR43, and GPR109A) and inhibition of histone deacetylases (HDACs) (*48*, *49*). These signaling pathways promote regulatory T cell differentiation, facilitate macrophage polarization toward a reparative phenotype, and suppress inflammasome activation, collectively limiting vascular inflammation and pathological remodeling (*49*). Consistent with these mechanisms, increased circulating SCFA levels in the present study were accompanied by restoration of IL-10 and TGF-β, suppression of proinflammatory cytokines, and attenuation of neointimal hyperplasia. Although the relative contribution of individual SCFAs was not specifically dissected, our findings suggest that coordinated restoration of the SCFA signaling network, rather than elevation of a single metabolite, constitutes a key component of gut-plaque axis reprogramming.

Seminal studies demonstrated that nonlethal inhibition of microbial TMA can attenuate atherosclerosis, positioning TMAO as a modifiable upstream driver rather than a passive biomarker (*50*–*52*). However, in the context of ISR, it remains unclear whether suppressing this pathway alone is sufficient and how this should be integrated with ecological modulation (*25*, *53*). These data suggest that metabolic inhibition must be implemented within an intestinally targeted, ecologically supportive framework. Coordinated suppression of TMAO, immune polarization, and neointimal remodeling was achieved by coupling TMA source inhibition with probiotic-supported ecosystem restoration, extending the relevance of the TMAO axis from atherosclerosis to restenotic disease.

Consistent with these histological improvements, immunofluorescence staining revealed markedly enhanced endothelial coverage, as indicated by increased CD31 expression, accompanied by reduced α-SMA–positive smooth muscle remodeling in both HFD- and ISR-associated vascular lesions (fig. S35). These findings provide direct in vivo evidence that the engineered synbiotic microspheres selectively promote endothelial regeneration while limiting pathological vascular remodeling, further supporting the restoration of vascular homeostasis.

### Alg-βG-*Lac.r*-DMB-Inu MS modulate gut-plaque axis homeostasis to enhance SCFA metabolism and ameliorate the immune microenvironment in ISR

Comprehensive 16*S* rRNA gene sequencing analyses were performed in ISR animal models to determine whether the therapeutic efficacy of Alg-βG-*Lac.r*-DMB-Inu MS is accompanied by coordinated remodeling of the gut microbiome along the gut-plaque axis. At the community level, ISR induction resulted in a pronounced loss of microbial richness and ecological integrity. α-Diversity analyses based on the ACE and Sobs indices revealed a significant reduction in observed taxa in ISR animals. Treatment with Alg-βG-*Lac.r*-DMB-Inu MS restored microbial richness toward the Ctrl state, outperforming both DMB-only and *Lac.r*-only interventions (Fig. 8A and fig. S36A). Rarefaction analyses confirmed adequate sequencing depth and stabilization of diversity estimates across all groups, supporting the robustness of downstream comparisons (fig. S36B).

**Fig. 8.**
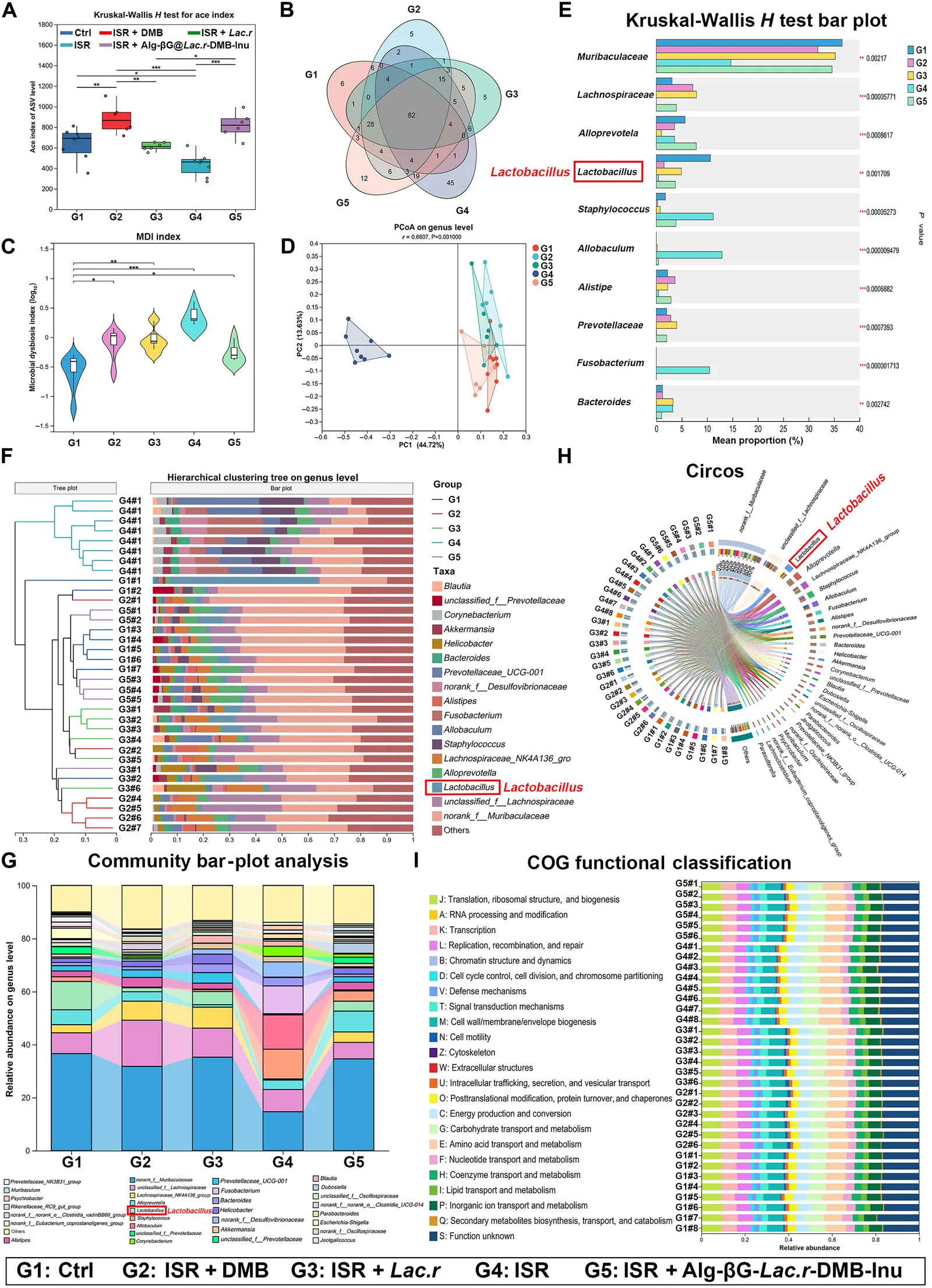
Microbiome remodeling via gut-plaque axis modulation by Alg-βG-*Lac.r*-DMB-Inu MS in ISR animal models. (**A**) α-diversity analysis based on the ACE index. (**B**) Venn diagram of operational taxonomic units across groups. (**C**) Microbial dysbiosis index (MDI) analysis of community stability across groups. (**D**) Principal coordinate analysis of gut microbial composition at the genus level. (**E**) Kruskal-Wallis *H* test for differential taxa. (**F**) Hierarchical clustering analysis at the genus level. (**G**) Circos plot for microbial community interactions. (**H**) Community bar plot analysis of genus-level compositions. (**I**) COG functional classification of microbial metabolic and functional pathways. G1, Ctrl; G2, ISR + DMB; G3, ISR + *Lac.r*; G4, ISR; and G5, ISR + Alg-βG-*Lac.r*-DMB-Inu. Significance: *P < 0.05; **P < 0.01; ***P < 0.001.

Consistent with diversity restoration, pangenome and rank abundance analyses revealed that Alg-βG-*Lac.r*-DMB-Inu MS reexpanded both dominant and low-abundance taxa, resulting in a more even and resilient microbial community structure compared with ISR controls (fig. S37). Operational taxonomic unit–based Venn analysis demonstrated substantial recovery of shared and unique genera in the composite microsphere group, indicative of reconstruction of a more complete microbial repertoire (Fig. 8B). Microbial dysbiosis index (MDI) analysis showed that, while ISR was associated with a marked elevation in ecological imbalance, Alg-βG-*Lac.r*-DMB-Inu MS normalized MDI values to near-baseline levels, reflecting restoration of community stability (Fig. 8C). This was supported by intra- and intergroup dissimilarity analyses, which revealed reduced community dispersion and enhanced compositional coherence following composite microsphere treatment (fig. S38).

Multivariate ordination analyses at the genus level revealed a global restructuring of microbial communities rather than isolated taxonomic shifts. Principal coordinate analysis, principal components analysis, and nonmetric multidimensional scaling showed that the Alg-βG-*Lac.r*-DMB-Inu MS group clustered distinctly away from ISR controls and toward Ctrl animals (Fig. 8D and fig. S39). Community typing analysis further indicated a treatment-driven transition from ISR-associated dysbiotic community types toward health-associated microbial configurations, supported by Calinski-Harabasz index optimization (fig. S40). At the taxonomic level, differential abundance analysis using the Kruskal-Wallis *H* test and LEfSe identified *Lactobacillus* as one of the most significantly enriched genera following Alg-βG-*Lac.r*-DMB-Inu MS administration, accompanied by coordinated suppression of ISR-associated proinflammatory and dysbiosis-linked taxa, including *Staphylococcus*, *Alistipes*, *Fusobacterium*, and *norank f Muribaculaceae* (Fig. 8E and figs. S41 to S44). Hierarchical clustering analysis confirmed that the overall microbial composition of the composite group closely resembled that of healthy controls. ISR and single-component treatments remained partially segregated (Fig. 8F).

At the community composition level, genus-level bar plot analysis revealed that ISR induced pronounced microbial dysbiosis, characterized by the expansion of inflammation-associated taxa and a concomitant depletion of beneficial commensals (Fig. 8G). Treatment with Alg-βG-*Lac.r-*DMB-Inu MS restored microbial community structure, enriching *Lactobacillus*-associated genera and rebalancing the relative abundance of multiple core taxa toward a control-like configuration. Network-level analyses demonstrated that Alg-βG-*Lac.r-*DMB-Inu MS reinforced microbial ecological stability. Circos plots and co-occurrence network topology revealed the reestablishment of dense and balanced microbial interaction networks centered on *Lactobacillus-*associated nodes, accompanied by increased proportions of connector and module-hub taxa, indicative of enhanced community resilience (Fig. 8H). Abundance-occupancy and persistence analyses showed a coordinated shift from transient to persistent microbial populations, supporting long-term ecosystem stabilization (fig. S45).

Functionally, COG-based predictions indicated that ISR reprogrammed microbial functional profiles toward stress response, membrane transport, and pathogenic metabolism (figs. S46 and S47). Alg-βG-*Lac.r-*DMB-Inu MS selectively enriched functional pathways related to carbohydrate transport and metabolism, energy production, and biosynthetic processes closely linked to SCFA generation (Fig. 8I and figs. S46 and S47). This coordinated structural, network, and functional reorganization provides a mechanistic basis for the enhanced SCFA production observed downstream and establishes a critical metabolic-immunological link between gut microbiota remodeling and immune microenvironment normalization along the gut-plaque axis.

Building on this microbiome restructuring and functional reprogramming, it was next investigated whether Alg-βG-*Lac.r*-DMB-Inu MS translate these shifts into coordinated tissue- and cell-level remodeling along the intestinal-plaque axis in situ. FISH targeting *Lac.r* revealed enhanced probiotic localization within the intestinal tract following microsphere treatment, accompanied by detectable *Lac.r*-associated signals in secondary immune tissues and atherosclerotic plaques (Fig. 9A). Quantitative flow cytometry–based FISH analysis confirmed significantly increased *Lac.r*-derived fluorescence across intestinal, splenic, and plaque compartments, indicating effective colonization and axis-wide microbial engagement (Fig. 9, B and C).

**Fig. 9.**
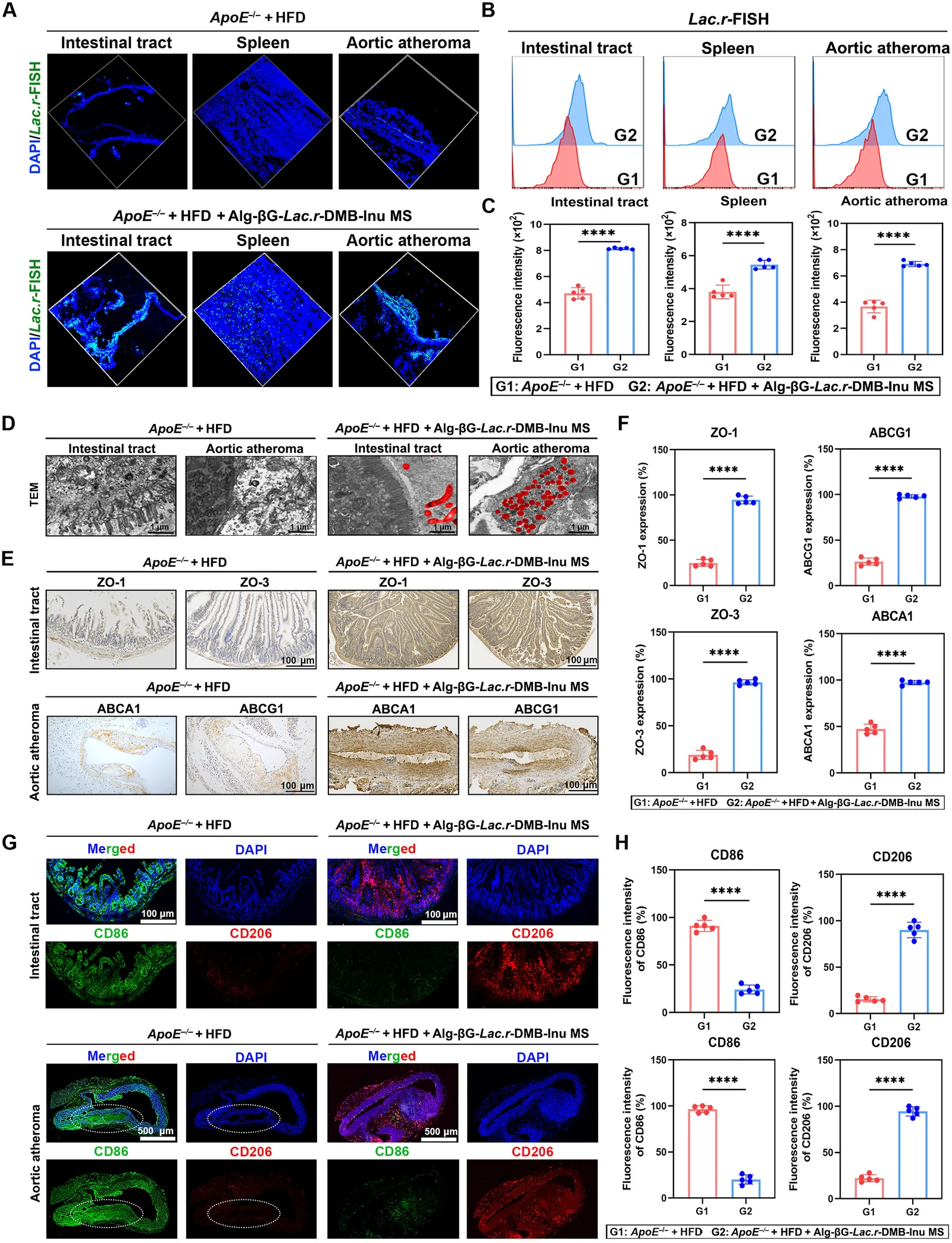
In vivo system-level modulation of the intestinal-plaque axis by Alg-βG-*Lac.r*-DMB-Inu MS. (**A**) *Lac.r*–fluorescence in situ hybridization (FISH) tissue localization assay for detecting *Lac.r* distribution within the intestinal tract, spleen, and aortic atheroma tissues; DAPI used for nuclear staining. (**B**) *Lac.r*-FISH flow cytometry analysis assessing *Lac.r*-associated fluorescence across tissues. (**C**) Quantitative analysis of FISH fluorescence intensity corresponding to the tissue localization in (A). (**D**) Representative transmission electron micrographs of intestinal tissues and atherosclerotic plaque sections. (**E**) IHC staining of intestinal tissues for tight junction proteins (ZO-1 and ZO-3) and of aortic atheroma sections for cholesterol efflux-related proteins (ABCA1 and ABCG1) across different treatment groups. (**F**) Quantitative analysis of IHC staining for ZO-1, ZO-3, ABCA1, and ABCG1 expression in (E). (**G**) Dual-label IF staining of intestinal and aortic atheroma tissues, with CD206 (red), CD86 (green), and DAPI (blue) for nuclear counterstaining. The white dashed region marks the atherosclerotic plaque. (**H**) Statistical histogram of CD206 and CD86 fluorescence signals from the dual-label IF staining in (G). Data are presented as means ± SEM across three biologically independent samples. Significance: ****P < 0.0001.

At the ultrastructural level, TEM revealed increased tissue-associated probiotic presence within both the intestinal epithelium and atherosclerotic plaques following microsphere treatment, indicating enhanced probiotic infiltration along the gut-plaque axis (Fig. 9D). Consistent with these structural improvements, immunohistochemical analyses showed pronounced up-regulation of tight junction proteins ZO-1 and ZO-3 in intestinal tissues, indicating reinforced barrier integrity (Fig. 9, E and F, and fig. S48). Atherosclerotic plaques from microsphere-treated animals displayed elevated expression of cholesterol efflux transporters ABCA1 and ABCG1, reflecting enhanced lipid-handling capacity within the plaque microenvironment (Fig. 9, E and F). Immune profiling revealed coordinated polarization shifts across compartments. Dual immunofluorescence staining demonstrated a significant reduction in CD86^+^ proinflammatory macrophages with a concomitant increase in CD206^+^ reparative macrophages in both intestinal and plaque tissues following microsphere administration (Fig. 9, G and H, and fig. S49). Notably, these immune alterations were directionally consistent across the gut-plaque axis, supporting the concept of synchronized immune remodeling rather than localized effects.

Untargeted metabolomics profiling in ISR and microsphere-treated animals was performed to determine whether the microbiome restructuring induced by Alg-βG-*Lac.r*-DMB-Inu MS translates to coordinated metabolic reprogramming in vivo. Global metabolite annotation and chemical classification analyses revealed a pronounced shift in metabolic composition following microsphere administration. Volcano plot–based differential analysis highlighted a broad up-regulation of SCFA-associated metabolites (Fig. 10, A to C, and fig. S50). Pathway enrichment further identified SCFA metabolism as one of the most substantially altered metabolic modules. Targeted quantification confirmed that SCFAs—particularly AA, PA, and BA—were markedly elevated in the microsphere-treated condition (Fig. 10D). In vitro fermentation assays demonstrated that *Lac.r*-derived SCFA production was significantly amplified in the presence of inulin, supporting prebiotic-assisted metabolic synergy within the composite microsphere design.

**Fig. 10.**
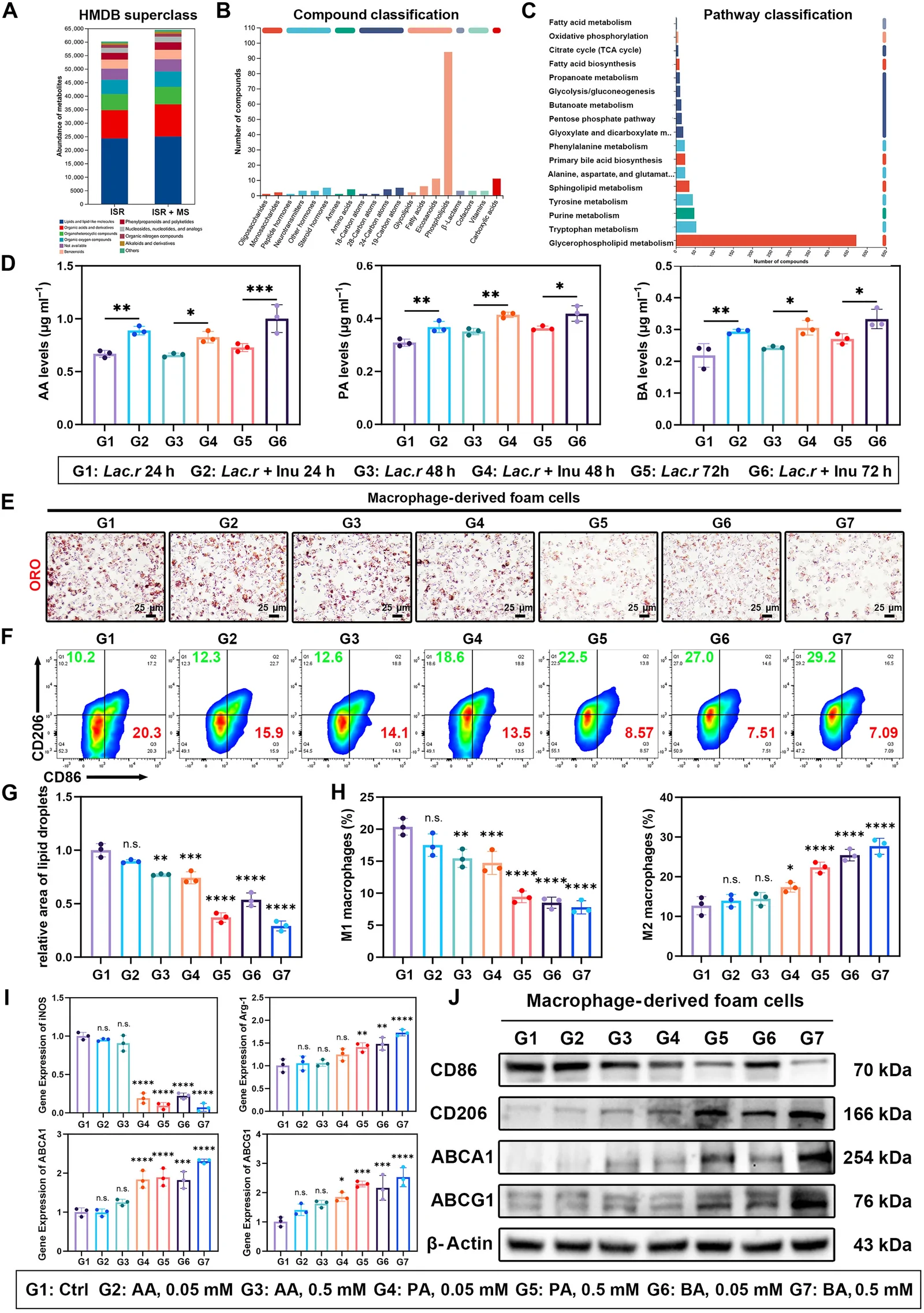
Alg-βG-*Lac.r*-DMB-Inu MS modulate gut-plaque axis homeostasis via SCFA metabolic pathways to reshape the immune microenvironment in ISR. (**A** to **C**) Untargeted metabolomics profiling of ISR and ISR + MS samples. Metabolites were annotated using the HMDB database (A), categorized by chemical class (B), and mapped to KEGG pathways (C). (**D**) Quantification of SCFAs (AA, PA, and BA) produced by *Lac.r* with or without inulin across different incubation periods. G1: *Lac.r*, 24 hours; G2: *Lac.r* + inulin, 24 hours; G3: *Lac.r*, 48 hours; G4: *Lac.r* + inulin, 48 hours; G5: *Lac.r*, 72 hours; G6: *Lac.r* + inulin, 72 hours. h, hours. (**E**) Oil Red O staining-based assessment of lipid accumulation in macrophage-derived foam cells exposed to SCFAs at indicated concentrations. (**F**) Flow cytometric analysis of macrophage polarization using CD86^+^ (M1 marker) and CD206^+^ (M2 marker) following SCFA stimulation. (**G** and **H**) Statistical histogram of lipid droplet area (G) and proportions of M1/M2 macrophages (H) corresponding to the flow cytometry panels. (**I**) RT-qPCR of macrophage polarization markers (iNOS and Arg-1) and cholesterol efflux transporters (ABCA1 and ABCG1) in foam cells treated with different SCFAs. (**J**) Western blot of CD86, CD206, ABCA1, and ABCG1 expression in macrophage-derived foam cells treated with SCFAs; β-actin was the internal control. G1: Ctrl; G2: AA, 0.05 mM; G3: AA, 0.5 mM; G4: PA, 0.05 mM; G5: PA, 0.5 mM; G6: BA, 0.05 mM; and G7: BA, 0.5 mM. Data are presented as means ± SEM across three biologically independent samples. Significance: *P < 0.05; **P < 0.01; ***P < 0.001; ****P < 0.0001. n.s., not significant.

Macrophage-derived foam cells were exposed to physiologically relevant concentrations of AA, PA, and BA (0.05 and 0.5 mM) to assess the functional relevance of SCFA enrichment. Lipid staining revealed dose-dependent attenuation of intracellular lipid accumulation following SCFA treatment (Fig. 10, E and G). Flow cytometric profiling showed a concentration-dependent reduction in CD86^+^ proinflammatory macrophages accompanied by an increase in CD206^+^ reparative macrophages (Fig. 10, F and H). At the molecular level, reverse transcription quantitative polymerase chain reaction (RT-qPCR) analyses revealed that SCFA exposure suppressed iNOS expression while up-regulating *ARG-1* and the cholesterol efflux genes *ABCA1* and *ABCG1* (Fig. 10I). These transcriptional changes were corroborated by Western blot analyses, which revealed concordant, concentration-dependent regulation of macrophage polarization markers and lipid transporters (Fig. 10J and fig. S51).

SCFA treatment significantly reduced intracellular ROS levels in macrophage-derived foam cells in a dose-responsive manner, with BA exerting the most pronounced antioxidant effect (fig. S52). Given that aberrant smooth muscle cell activation represents a central pathological driver of ISR, the effects of SCFAs on smooth muscle–derived foam cells were further investigated. F-actin cytoskeletal staining revealed that increasing concentrations of AA, PA, and BA progressively attenuated stress fiber organization and cytoskeletal activation, indicating suppression of inflammation-associated smooth muscle hyperactivity (fig. S53). SCFA treatment accelerated endothelial wound closure in LPS-challenged HUVECs, with concentration-dependent enhancement observed at 12 and 24 hours, particularly in BA-treated groups (fig. S54).

SCFAs are increasingly recognized as central immunometabolic regulators linking gut microbiota activity to host inflammatory and lipid-handling programs (*54*, *55*). Among them, acetate, propionate, and butyrate exert overlapping as well as distinct biological functions (*55*, *56*). Acetate serves as a major systemic metabolite influencing lipid synthesis and energy balance; propionate suppresses inflammatory signaling and hepatic cholesterol biosynthesis; and butyrate acts as a potent immunomodulator through G protein–coupled receptor signaling and HDAC inhibition, shaping macrophage polarization and cholesterol efflux pathways (*56*–*58*). Prior studies have associated SCFAs, particularly butyrate, with attenuation of atherosclerosis via receptor-dependent and epigenetic mechanisms (*54*, *59*). The present study advances this framework by establishing a functional continuum from microbiota restructuring to SCFA enrichment and, critically, to direct regulation of foam cell behavior within atherosclerotic lesions. This work demonstrates that SCFAs reduce intracellular lipid accumulation, alleviate oxidative stress, and suppress proinflammatory macrophage bias, restoring a cholesterol-processing phenotype favorable to plaque stabilization. These effects position SCFAs as active effectors executing gut-plaque axis reprogramming rather than passive metabolic byproducts. By linking microbial metabolism to vascular immune remodeling, these findings provide mechanistic support for SCFA-centered strategies as a feasible, systemically actionable approach for atherosclerosis and ISR interventions.

### Systemic biosafety assessment of Alg-βG-*Lac.r-*DMB-Inu MS

To evaluate the in vivo biosafety of Alg-βG-*Lac.r-*DMB-Inu MS, comprehensive histopathological and hematological analyses were performed across all treatment groups. H&E staining revealed well-preserved tissue architecture in major organs (including the heart, liver, spleen, lungs, and kidneys) in mice from the Ctrl, FMT, Alg-βG-DMB, Alg-βG-*Lac.r*, and Alg-βG-*Lac.r*-DMB-Inu MS groups. No inflammatory infiltration, parenchymal necrosis, fibrosis, or structural disruption were observed, indicating the absence of detectable organ toxicity following long-term microsphere administration (fig. S55).

Serum biochemical analyses showed that liver function markers (alkaline phosphatase, alanine aminotransferase, and aspartate aminotransferase) remained within physiological ranges across all groups; no significant intergroup differences were observed. Renal function–related parameters (including blood urea nitrogen, creatinine, and uric acid) were similarly stable, suggesting no impairment of renal clearance or metabolic burden induced by microsphere treatment (fig. S56). Routine hematological indices (including red blood cell count, hemoglobin concentration, and white blood cell count) showed no abnormal alterations, indicating preserved hematopoietic homeostasis and the absence of overt systemic inflammatory responses. Collectively, these findings demonstrate that Alg-βG-*Lac.r*-DMB-Inu MS exhibit favorable biocompatibility and systemic safety under long-term in vivo administration, supporting their translational potential as an oral platform for gut-plaque axis modulation and ISR prevention.

In this study, we reconceptualize ISR as a manifestation of systemic, axis-level dysfunction rather than a purely lesion-centered vascular complication. By integrating clinical observations with cross-species mechanistic analyses, we demonstrate that pathological vascular remodeling is sustained by a maladaptive gut-immune-vascular axis linking microbial dysbiosis, intestinal barrier disruption, immunometabolic imbalance, and plaque inflammation into a self-reinforcing circuit. This framework challenges the prevailing paradigm that restenosis arises predominantly from local vascular injury and incomplete endothelial repair, and instead positions ISR within a broader class of chronic inflammatory vascular diseases driven by cross-organ coupling. This axis-oriented perspective provides a unifying explanation for the limited durability of existing lesion-targeted interventions and reveals upstream, system-level vulnerabilities that can be therapeutically exploited.

## MATERIALS AND METHODS

### Materials, reagents, and animals

*Lac.r* was obtained from the Shanghai Preservation Biotechnology Center. De Man, Rogosa, and Sharpe agar and broth were purchased from Solarbio (Beijing, China); DMB and inulin were obtained from Shanghai Yuanye Bio-Technology. The Calcein-AM/PI Live/Dead Cell Viability Kit (Beyotime Biotechnology, catalog no. C2015S, Shanghai, China) was purchased from Beyotime Biotechnology. Detailed commercial reagents are shown in table S1. Detailed commercial antibodies are shown in table S2.

Male *ApoE*^−/−^ mice (15 to 20 g), Sprague-Dawley rats (250 to 300 g), and male New Zealand white rabbits (2.5 to 3.0 kg) were purchased from Hangzhou Hansi Biotechnology. All animals were housed under specific pathogen–free conditions with controlled temperature (25° ± 2°C) and humidity (50 ± 5%) and a 12-hour light/dark cycle.

### Microsphere fabrication and characterization

Alg-βG was synthesized by cross-linking 1.5 wt % sodium alginate with 1 wt % ethylenediamine-aminated β-glucan at 4°C and pH 7.5 for 4 hours, followed by dialysis (3500 Da MWCO) and lyophilization. Microspheres were fabricated via microfluidic emulsification using a dispersed phase {*Lac.r* [1 × 10^9^ colony-forming units (CFU)/ml], 1% DMB, 2% inulin, and 1% Alg-βG} and a continuous phase (94.8% liquid paraffin, 5% Span 80, and 0.2% calcium iodide) at flow rates of 4 to 6 and 300 to 400 μl/min, respectively. Particles were washed with isopropanol/*n*-hexane (95:5, v/v). For release studies, microspheres were incubated in phosphate-buffered saline (PBS; pH 1.2 or 7.4, ±10 U of β-glucanase) at 37°C; aliquots collected at designated intervals were analyzed for *Lac.r* (plate counting), DMB (gas chromatography), and inulin. Particle size and morphology were characterized by dynamic light scattering, SEM, and TEM (2% uranyl acetate negative staining). For storage stability, encapsulated *Lac.r* was maintained at 4° or 24°C for up to 6 months, and viability was assessed by SYTO9/propidium iodide staining.

### Bacterial metabolic activity

*Lac.r* metabolic activity was assessed in a carbon-free basal medium with inulin as the sole carbon source (1 × 10^9^ CFU/ml). Acetate, propionate, and butyrate in culture supernatants were quantified by ELISA at 24, 48, and 72 hours.

### Cell culture and functional assays

HUVECs (Service-bio Technology, catalog no. STCC12103, Wuhan, China) and MOVAS and RAW264.7 cells (purchased from the American Type Culture Collection) were cultured in RPMI 1640 or Dulbecco’s modified Eagle’s medium supplemented with 10% fetal bovine serum and 1% penicillin-streptomycin at 37°C under 5% CO_2_. Foam cells were induced with oxLDL (100 μg/ml) for 48 hours. Cytocompatibility, ROS generation, F-actin organization, proliferation (EdU, 10 μM), and macrophage polarization (F4/80, CD11b, CD86, and CD206) were evaluated by standard live/dead, 2′,7′-dichlorodihydrofluorescein diacetate, phalloidin, EdU, and flow cytometric assays, respectively. Endothelial migration was assessed by scratch wound healing assay. Antibacterial activity against *E. coli* and *S. aureus* was determined after 24 hours of exposure to MSLs (1:1, v/v).

### Western blot

Cell lysates were prepared in radioimmunoprecipitation assay buffer supplemented with phenylmethylsulfonyl fluoride and phosphatase inhibitors, and protein concentrations were determined by BCA assay. Equal amounts of protein (50 to 100 μg) were separated by SDS–polyacrylamide gel electrophoresis and transferred to polyvinylidene difluoride membranes. After blocking with 5% skim milk, membranes were incubated with primary antibodies (1:1000) overnight at 4°C and species-specific secondary antibodies (1:10,000) at room temperature. Protein bands were visualized by enhanced chemiluminescence and semiquantified using ImageJ.

### Enzyme-linked immunosorbent assay

Plasma, serum, and tissue homogenate levels of IL-1β, TNF-α, IL-18, SCFAs (acetate, propionate, and butyrate), and TMAO were determined using commercial ELISA kits (Wuhan EIAab Science and Wuhan Jilid Biotechnology) according to the manufacturer’s protocols. Sample concentrations were interpolated from standard curves and expressed as picograms per milliliter for plasma/serum or normalized to total protein content for tissue homogenates.

### Fluorescence in situ hybridization

*Lac.r* localization was detected using a species-specific probe (5′-GCTGCCTCCCGTAGGAGT-3′) on frozen tissue sections and microbial specimens. Following fixation, permeabilization, and overnight hybridization, nuclei were counterstained with 4′,6-diamidino-2-phenylindole (DAPI) and imaged by fluorescence microscopy.

### Real-time qPCR

Total RNA was extracted using an RNA-Quick Purification Kit (YEASEN) and treated with deoxyribonuclease I (Thermo Fisher Scientific). First-strand cDNA was synthesized using the RevertAid First Strand cDNA Synthesis Kit (Thermo Fisher Scientific). qPCR was performed on a CFX96 system (Bio-Rad) using SYBR Green Master Mix (Vazyme) with glyceraldehyde-3-phosphate dehydrogenase as the internal reference. Relative gene expression was calculated by the 2^−ΔΔCT^ method. Primer sequences are provided in table S3.

### Animal models and treatments

Male C57BL/6J mice were fasted for 6 hours before oral gavage of Cy5.5-labeled free *Lac.r* or Alg-βG-*Lac.r*-DMB-Inu microspheres (1 × 10^9^ CFU in 200 μl of PBS). Gastrointestinal distribution was monitored by ex vivo fluorescence imaging (IVIS Lumina Series III, Revvity) at 2, 4, 24, 48, and 96 hours postadministration, and viable bacteria were quantified by plating tissue homogenates on MRS agar. For gastrointestinal persistence assays, fecal pellets were collected every 2 days, homogenized, and plated on MRS agar supplemented with cycloheximide (100 μg/ml) under anaerobic conditions. For FMT, fecal donors (C57BL/6J mice maintained on HFD for 4 weeks) were selected, and recipient *ApoE^−^/^−^* mice were pretreated with a broad-spectrum antibiotic cocktail (ampicillin, neomycin, and vancomycin) for 5 days before receiving 200 μl of fecal slurry (1 × 10^9^ CFU equivalent) by gavage every other day for 4 weeks. ISR was established in rats and rabbits by carotid balloon injury followed by bare-metal stent implantation (8 mm by 2.5 mm, BD Biosciences) under HFD feeding.

### In vivo imaging and histology

MRI was performed on a 3.0-T Ingenia scanner (Philips) with a 5-cm eight-channel surface coil using T2W-TSE sequences (TR/TE = 2500/45 ms) with respiratory gating. CTA was conducted on a SOMATOM Force scanner (Siemens; 80 kVp, 50 mAs) after intravenous administration of iopromide (1.1 ml/kg); images were reconstructed by volume rendering and maximum intensity projection. ROIs were manually delineated by two blinded investigators, and lumen stenosis or plaque volume was quantified using ImageJ. After treatment, mice were transcardially perfused with saline; aortas were harvested, fixed in 4% PFA, sectioned (5 μm) or longitudinally opened, and stained with Oil Red O or Masson’s trichrome for morphometric analysis. Immunofluorescence staining for CD86, CD31, α-SMA, and CD206 was performed on aortic root cryosections and imaged by CLSM (TCS SP8, Leica).

### Safety evaluation

Healthy female mice (*n* = 5 per group) received the respective treatments for 4 weeks. Blood was collected for complete blood count (WBC, RBC, and HGB) and serum biochemical analysis of liver (ALP, AST, and ALT) and renal (BUN, CR, and UA) function. Major organs were harvested for H&E staining.

### Gut microbiota analysis

Fecal samples underwent 16*S* rRNA gene sequencing to characterize microbial composition, ecological structure, and functional potential. Community diversity, stability, and interaction networks were analyzed to assess microbiota restructuring following treatment.

### Statistical analysis

In vitro and ex vivo experiments were performed with three independent biological replicates (*n* = 3); in vivo studies used five animals per group (*n* = 5). All quantitative data are presented as means ± SEM. Statistical analyses were conducted using GraphPad Prism (version 9.5.1; GraphPad software) and SPSS Statistics (version 26.0; IBM Corp., Armonk, NY, USA). Appropriate nonparametric tests or one-way analysis of variance (ANOVA) with post hoc corrections were applied as indicated. Statistical significance was defined as *P* < 0.05.

### Ethics statement

All human- and animal-related procedures were conducted in accordance with institutional guidelines and national regulations. Animal studies were conducted under protocols approved by the Animal Ethics Committee of Lishui University (approval no. DWYX2026005), following the guidelines for the care and use of laboratory animals of Wenzhou Medical University. Human subject research was approved by the Institutional Review Board of The Fifth Affiliated Hospital of Wenzhou Medical University [approval no. 2025(I)-142-01]. Written informed consent was obtained from all participants. All animal experiments were approved by the Animal Care and Use Committee of Lishui Central Hospital and performed in compliance with established ethical standards. The study included 40 participants. Participants were recruited from The Fifth Affiliated Hospital of Wenzhou Medical University and self-identified as Asian (100%).

## References

[1] A. B. Herman, J. Candia, D. M. Wilson III, S. Donega, M. Picard, L. Ferrucci, Molecular damage associated with ageing drives inflammation in cardiovascular disease. Nat. Rev. Cardiol. 23, 486–499 (2026). 10.1038/s41569-026-01253-3 PMC12951825 41571994

[2] M. Llena-Meler, A. Canfran-Duque, J. Madrigal-Matute, N. Rotllan, Lipid metabolic alterations in cancer: Common pathophysiology with cardiovascular disease. Semin. Cancer Biol. 119, 83–105 (2026). 10.1016/j.semcancer.2026.01.003 41520912

[3] D. Tousoulis, T. Guzik, T. Padro, D. J. Duncker, G. De Luca, E. Eringa, M. Vavlukis, A. S. Antonopoulos, T. Katsimichas, E. Cenko, A. Djordjevic-Dikic, I. Fleming, O. Manfrini, D. Trifunovic, C. Antoniades, F. Crea, Mechanisms, therapeutic implications, and methodological challenges of gut microbiota and cardiovascular diseases: A position paper by the ESC Working Group on Coronary Pathophysiology and Microcirculation. Cardiovasc. Res. 118, 3171–3182 (2022). 10.1093/cvr/cvac057 PMC11023489 35420126

[4] E. Barbato, M. McEntegart, T. Gori, The year in cardiovascular medicine 2022: The top 10 papers in interventional cardiology. Eur. Heart J. 44, 259–261 (2023). 10.1093/eurheartj/ehac778 36587942

[5] C. Baaten, M. Nagy, W. Bergmeier, H. M. H. Spronk, P. E. J. van der Meijden, Platelet biology and function: Plaque erosion vs. rupture. Eur. Heart J. 45, 18–31 (2024). 10.1093/eurheartj/ehad720 PMC10757869 37940193

[6] I. Nedeljkovic, Assessment of depression and anxiety in patients before and after percutaneous coronary intervention: A step forward in cardiac rehabilitation? Eur. J. Prev. Cardiol. 25, 1015–1016 (2018). 10.1177/2047487318774094 29707962

[7] M. Takeuchi, T. Dohi, T. Fukase, R. Nishio, N. Takahashi, H. Endo, S. Doi, Y. Kato, I. Okai, H. Iwata, S. Okazaki, K. Isoda, K. Miyauchi, T. Minamino, Comparison of clinical outcomes between percutaneous coronary intervention for the de novo lesion versus in-stent restenosis lesion. Eur. Heart J. 42, ehab724.1118 (2021). 10.1007/s12928-021-00792-5 34224098

[8] M. Barradas, G. Pires-Morais, B. Melica, M. Goncalves, C. Espada Guerreiro, L. Santos, A. Rodrigues, P. Braga, R. Fontes-Carvalho, Intracoronary imaging guided percutaneous coronary intervention of stent restenosis: Does it make a difference? Eur. Heart J. 44, ehad655.1468 (2023).

[9] I. D. Moussa, D. Mohananey, J. Saucedo, G. W. Stone, R. W. Yeh, K. F. Kennedy, R. Waksman, P. Teirstein, J. W. Moses, C. Simonton, Trends and outcomes of restenosis after coronary stent implantation in the United States. J. Am. Coll. Cardiol. 76, 1521–1531 (2020). 10.1016/j.jacc.2020.08.002 32972528

[10] H. Tilg, T. E. Adolph, A gut bacterium tackles atherosclerosis. Nat. Metab. 4, 1221–1222 (2022). 10.1038/s42255-022-00648-z 36253616

[11] J. L. Leslie, B. H. Annex, The microbiome and endothelial function. Circ. Res. 123, 1015–1016 (2018). 10.1161/CIRCRESAHA.118.313813 PMC6317883 30355167

[12] A. R. Fatkhullina, I. O. Peshkova, A. Dzutsev, T. Aghayev, J. A. McCulloch, V. Thovarai, J. H. Badger, R. Vats, P. Sundd, H. Y. Tang, A. V. Kossenkov, S. L. Hazen, G. Trinchieri, S. I. Grivennikov, E. K. Koltsova, An interleukin-23-interleukin-22 axis regulates intestinal microbial homeostasis to protect from diet-induced atherosclerosis. Immunity 49, 943–957.e9 (2018). 10.1016/j.immuni.2018.09.011 PMC6257980 30389414

[13] A. L. Komaroff, The microbiome and risk for atherosclerosis. JAMA 319, 2381–2382 (2018). 10.1001/jama.2018.5240 29800043

[14] C. Fang, K. Zuo, Y. Fu, J. Li, H. Wang, L. Xu, X. Yang, Dysbiosis of gut microbiota and metabolite phenylacetylglutamine in coronary artery disease patients with stent stenosis. Front. Cardiovasc. Med. 9, 832092 (2022). 10.3389/fcvm.2022.832092 PMC8990098 35402559

[15] J. E. Ellis, R. Heuser, D. S. Missan, D. Martinez, A. Heningburg, M. Shabilla, R. Schwartz, S. Fry, Evidence for polymicrobial communities in explanted vascular filters and atheroma debris. Mol. Cell. Probes 33, 65–77 (2017). 10.1016/j.mcp.2017.04.001 28411089

[16] C. A. Cason, T. M. Kuntz, E. B. Chen, K. Wun, M. J. Nooromid, L. Xiong, N. R. Gottel, K. G. Harris, T. C. Morton, M. J. Avram, E. B. Chang, J. A. Gilbert, K. J. Ho, Microbiota composition modulates inflammation and neointimal hyperplasia after arterial angioplasty. J. Vasc. Surg. 71, 1378–1389.e3 (2020). 10.1016/j.jvs.2019.06.208 PMC7096256 32035769

[17] Y. Fu, Y. Yang, C. Fang, X. Liu, Y. Dong, L. Xu, M. Chen, K. Zuo, L. Wang, Prognostic value of plasma phenylalanine and gut microbiota-derived metabolite phenylacetylglutamine in coronary in-stent restenosis. Front. Cardiovasc. Med. 9, 944155 (2022). 10.3389/fcvm.2022.944155 PMC9468445 36110409

[18] E. E. Ziganshina, D. M. Sharifullina, A. P. Lozhkin, R. N. Khayrullin, I. M. Ignatyev, A. M. Ziganshin, Bacterial communities associated with atherosclerotic plaques from Russian individuals with atherosclerosis. PLOS ONE 11, e0164836 (2016). 10.1371/journal.pone.0164836 PMC5063344 27736997

[19] Q. Wang, Y. He, X. Li, T. Zhang, M. Liang, G. Wang, J. Zhao, H. Zhang, W. Chen, *Lactobacillus reuteri* CCFM8631 alleviates hypercholesterolaemia caused by the Paigen atherogenic diet by regulating the gut microbiota. Nutrients 14, 1272 (2022). 10.3390/nu14061272 PMC8953203 35334930

[20] J. Abuqwider, M. Altamimi, G. Mauriello, *Limosilactobacillus reuteri* in health and disease. Microorganisms 10, 522 (2022). 10.3390/microorganisms10030522 PMC8953724 35336098

[21] J. Jing, J. Guo, R. Dai, C. Zhu, Z. Zhang, Targeting gut microbiota and immune crosstalk: Potential mechanisms of natural products in the treatment of atherosclerosis. Front. Pharmacol. 14, 1252907 (2023). 10.3389/fphar.2023.1252907 PMC10504665 37719851

[22] M. Orman, S. Bodea, M. A. Funk, A. M. Campo, M. Bollenbach, C. L. Drennan, E. P. Balskus, Structure-guided identification of a small molecule that inhibits anaerobic choline metabolism by human gut bacteria. J. Am. Chem. Soc. 141, 33–37 (2019). 10.1021/jacs.8b04883 PMC6475491 30557011

[23] Z. Wang, A. B. Roberts, J. A. Buffa, B. S. Levison, W. Zhu, E. Org, X. Gu, Y. Huang, M. Zamanian-Daryoush, M. K. Culley, A. J. DiDonato, X. Fu, J. E. Hazen, D. Krajcik, J. A. DiDonato, A. J. Lusis, S. L. Hazen, Non-lethal inhibition of gut microbial trimethylamine production for the treatment of atherosclerosis. Cell 163, 1585–1595 (2015). 10.1016/j.cell.2015.11.055 PMC4871610 26687352

[24] Q. Zhu, Z. Chen, D. Wang, X. Jiao, Y. Luan, M. Wang, R. Luo, Y. Wang, G. Fu, Y. Wang, W. Zhang, Microenvironment-responsive coating for vascular stents to regulate coagulation-inflammation interaction and promote vascular recovery. Bioact. Mater. 48, 443–457 (2025). 10.1016/j.bioactmat.2025.02.031 PMC11909720 40093305

[25] I. T. Bajeu, A. G. Niculescu, A. Scafa-Udriște, E. Andronescu, Intrastent restenosis: A comprehensive review. Int. J. Mol. Sci. 25, 1715 (2024). 10.3390/ijms25031715 PMC10855438 38338993

[26] W. Chen, S. Zhang, J. Wu, T. Ye, S. Wang, P. Wang, D. Xing, Butyrate-producing bacteria and the gut-heart axis in atherosclerosis. Clin. Chim. Acta 507, 236–241 (2020). 10.1016/j.cca.2020.04.037 32376324

[27] H. Shi, M. Wu, X. Wu, Z. Liu, S. Jiang, G. Li, Y. Yang, Y. Fu, Q. Wang, G. Zhang, L. Cheng, Multi-omics integration reveals functional signatures of gut microbiome in atherosclerosis. Gut Microbes 17, 2542384 (2025). 10.1080/19490976.2025.2542384 PMC12341049 40785047

[28] A. Mastrangelo, I. Robles-Vera, D. Mañanes, M. Galán, M. Femenía-Muiña, A. Redondo-Urzainqui, R. Barrero-Rodríguez, E. Papaioannou, J. Amores-Iniesta, A. Devesa, M. Lobo-González, A. Carreras, K. R. Beck, S. Ivarsson, A. Gummesson, G. Georgiopoulos, M. Rodrigo-Tapias, S. Martínez-Cano, I. Fernández-López, V. Nuñez, A. Ferrarini, N. Inohara, K. Stamatelopoulos, A. Benguría, D. Cibrian, F. Sánchez-Madrid, V. Alonso-Herranz, A. Dopazo, C. Barbas, J. Vázquez, J. A. López, A. González-Martín, G. Nuñez, K. Stellos, G. Bergström, F. Bäckhed, V. Fuster, B. Ibañez, D. Sancho, Imidazole propionate is a driver and therapeutic target in atherosclerosis. Nature 645, 254–261 (2025). 10.1038/s41586-025-09263-w PMC12408353 40670786

[29] D. Yao, C. S. Ranadheera, C. Shen, W. Wei, L.-Z. Cheong, Milk fat globule membrane: Composition, production and its potential as encapsulant for bioactives and probiotics. Crit. Rev. Food Sci. Nutr. 64, 12336–12351 (2024). 10.1080/10408398.2023.2249992 37632418

[30] B. Chen, W. Pu, J. Zhang, Functional microgel enables effective delivery and colonization of probiotics for treating colitis. ACS Cent. Sci. 9, 1260–1262 (2023). 10.1021/acscentsci.3c00773 PMC10375571 37521798

[31] Y. Zhang, J. Hu, X. Song, J. Dai, Z. Tang, G. Huang, W. Jiao, Y. Wu, C. Wang, L. Du, Y. Jin, The effects of *Lactobacillus reuteri* microcapsules on radiation-induced brain injury by regulating the gut microenvironment. Food Funct. 14, 10041–10051 (2023). 10.1039/d3fo03008c 37843434

[32] Y. Zheng, J. Dou, C. Sun, H. Liu, S. Zhang, W. Wu, Application of modified β-glucan/montmorillonite- based sodium alginate microsphere adsorbents for removing deoxynivalenol and zearalenone from corn steep liquor. Food Control 158, 110204 (2024).

[33] G. Wang, F. Yan, Y. Wang, Y. Liu, J. Cui, Z. Yu, L. Feng, T. D. James, C. Wang, Y. Kong, Visual sensing of β-glucosidase from intestinal fungus in the generation of cytotoxic icarisid II. Front. Chem. 10, 919624 (2022). 10.3389/fchem.2022.919624 PMC9184716 35692694

[34] P. Peng, T. Feng, X. Yang, C. Nie, L. Yu, R. Ding, Q. Zhou, X. Jiang, P. Li, Gastrointestinal microenvironment responsive nanoencapsulation of probiotics and drugs for synergistic therapy of intestinal diseases. ACS Nano 17, 14718–14730 (2023). 10.1021/acsnano.3c02646 37490035

[35] D. Patoulias, G. Kassimis, N. Fragakis, Sodium-glucose co-transporter-2 inhibitors, gut microbiota, and atherosclerotic cardiovascular disease: Another mountain to conquer? Pharmacol. Res. 187, 106619 (2023). 10.1016/j.phrs.2022.106619 36535570

[36] E. S. Kim, B. H. Yoon, S.-M. Lee, M. Choi, E. H. Kim, B. W. Lee, S.-Y. Kim, C.-G. Pack, Y. H. Sung, I.-J. Baek, C. H. Jung, T.-B. Kim, J.-Y. Jeong, C. H. Ha, Fecal microbiota transplantation ameliorates atherosclerosis in mice with C1q/TNF-related protein 9 genetic deficiency. Exp. Mol. Med. 54, 103–114 (2022). 10.1038/s12276-022-00728-w PMC8894390 35115674

[37] G. S. Getz, C. A. Reardon, Diet, microbes, and murine atherosclerosis. Arterioscler. Thromb. Vasc. Biol. 38, 2269–2271 (2018). 10.1161/ATVBAHA.118.311513 PMC6207197 30354225

[38] W. K. Wu, E. A. Ivanova, A. N. Orekhov, Gut microbiome: A possible common therapeutic target for treatment of atherosclerosis and cancer. Semin. Cancer Biol. 70, 85–97 (2021). 10.1016/j.semcancer.2020.06.017 32610150

[39] M. J. Saadh, B. Bazghandi, H. Jamialahmdi, F. Rahimzadeh-Bajgiran, F. Forouzanfar, S. A. Esmaeili, E. Saburi, Therapeutic potential of lipid-lowering probiotics on the atherosclerosis development. Eur. J. Pharmacol. 971, 176527 (2024). 10.1016/j.ejphar.2024.176527 38554932

[40] Y. Zhao, Y. Liu, J. Shan, X. Xu, C. Zhang, Z. Liu, X. Li, Z. Zhong, Y. Gao, K. Ren, D. Jiao, J. Ren, P. Wu, Y. Jiang, X. Han, Anti-inflammatory coupled anti-angiogenic airway stent effectively suppresses tracheal in-stents restenosis. J. Nanobiotechnol. 23, 59 (2025). 10.1186/s12951-024-03087-y PMC11776288 39881307

[41] C. Nicolais, V. Lakhter, H. U. H. Virk, P. Sardar, C. Bavishi, B. O’Murchu, S. Chatterjee, Therapeutic options for in-stent restenosis. Curr. Cardiol. Rep. 20, 7 (2018). 10.1007/s11886-018-0952-4 29435779

[42] E. Dinakis, J. A. O’Donnell, F. Z. Marques, The gut-immune axis during hypertension and cardiovascular diseases. Acta Physiol. 240, e14193 (2024). 10.1111/apha.14193 38899764

[43] S. Carloni, M. Rescigno, Unveiling the gut-brain axis: Structural and functional analogies between the gut and the choroid plexus vascular and immune barriers. Semin. Immunopathol. 44, 869–882 (2022). 10.1007/s00281-022-00955-3 PMC9301898 35861857

[44] Z. Chen, Q. Zhu, H. Xu, Y. Hu, Y. Wang, Z. Chen, Y. Wang, X. Huang, G. Fu, B. Ma, W. Zhang, Nano-functionalized probiotic treats atherosclerosis via inhibiting intestinal microbiota-TMA-TMAO axis. Nat. Commun. 16, 11294 (2025). 10.1038/s41467-025-66448-7 PMC12722705 41390683

[45] Q. Su, S. Kaluzny, Proatherogenic gut-microbiome metabolite trimethylamine elicits a gut-liver circuit to regulate TMAO metabolism and atherosclerosis via mediation of CREBH. Eur. Heart J. 42, ehab724.3347 (2021).

[46] S. H. Zeisel, M. Warrier, Trimethylamine *N*-oxide, the microbiome, and heart and kidney disease. Annu. Rev. Nutr. 37, 157–181 (2017). 10.1146/annurev-nutr-071816-064732 28715991

[47] W. H. Tang, Z. Wang, B. S. Levison, R. A. Koeth, E. B. Britt, X. Fu, Y. Wu, S. L. Hazen, Intestinal microbial metabolism of phosphatidylcholine and cardiovascular risk. N. Engl. J. Med. 368, 1575–1584 (2013). 10.1056/NEJMoa1109400 PMC3701945 23614584

[48] C. H. Kim, Short-, medium-, versus long-chain fatty acids: Mechanisms of immunomodulation and disease pathogenesis. Cell. Mol. Immunol. 23, 594–606 (2026). 10.1038/s41423-026-01412-z PMC13222355 42056219

[49] M. D. Docampo, M. B. da Silva, A. Lazrak, K. B. Nichols, S. R. Lieberman, A. E. Slingerland, G. K. Armijo, Y. Shono, C. Nguyen, S. Monette, E. Dwomoh, N. Lee, C. D. Geary, S. M. Perobelli, M. Smith, K. A. Markey, S. A. Vardhana, A. I. Kousa, E. Zamir, I. Greenfield, J. C. Sun, J. R. Cross, J. U. Peled, R. R. Jenq, C. K. Stein-Thoeringer, M. R. M. van den Brink, Alloreactive T cells deficient of the short-chain fatty acid receptor GPR109A induce less graft-versus-host disease. Blood 139, 2392–2405 (2022). 10.1182/blood.2021010719 PMC9012131 34653248

[50] T. Luo, Z. Guo, D. Liu, Z. Guo, Q. Wu, Q. Li, R. Lin, P. Chen, C. Ou, M. Chen, Deficiency of PSRC1 accelerates atherosclerosis by increasing TMAO production via manipulating gut microbiota and flavin monooxygenase 3. Gut Microbes 14, 2077602 (2022). 10.1080/19490976.2022.2077602 PMC9135421 35613310

[51] S. R. Ma, Q. Tong, Y. Lin, L.-B. Pan, J. Fu, R. Peng, X.-F. Zhang, Z.-X. Zhao, Y. Li, J.-B. Yu, L. Cong, P. Han, Z.-W. Zhang, H. Yu, Y. Wang, J.-D. Jiang, Berberine treats atherosclerosis via a vitamine-like effect down-regulating Choline-TMA-TMAO production pathway in gut microbiota. Signal Transduct. Target. Ther. 7, 207 (2022). 10.1038/s41392-022-01027-6 PMC9259588 35794102

[52] X. Zhu, L. Zhao, Y. Wang, X. Hu, Y. Zhu, X. Yang, Dietary titanium dioxide particles (E171) promote diet-induced atherosclerosis through reprogramming gut microbiota-mediated choline metabolism in APOE^−/−^ mice. J. Hazard. Mater. 436, 129179 (2022). 10.1016/j.jhazmat.2022.129179 35739712

[53] E. Shlofmitz, N. Khalid, R. Waksman, In-stent restenosis: A second chance to get it right. J. Am. Coll. Cardiol. 76, 1389–1390 (2020). 10.1016/j.jacc.2020.06.081 32912453

[54] T. Hu, Q. Wu, Q. Yao, K. Jiang, J. Yu, Q. Tang, Short-chain fatty acid metabolism and multiple effects on cardiovascular diseases. Ageing Res. Rev. 81, 101706 (2022). 10.1016/j.arr.2022.101706 35932976

[55] Y. Wang, W. Dou, X. Qian, H. Chen, Y. Zhang, L. Yang, Y. Wu, X. Xu, Advancements in the study of short-chain fatty acids and their therapeutic effects on atherosclerosis. Life Sci. 369, 123528 (2025). 10.1016/j.lfs.2025.123528 40049368

[56] T. Wada, T. Senokuchi, Y. Shi, T. Furusho, Y. Morita, M. Sarie, S. Hanatani, K. Fukuda, N. Ishii, T. Matsumura, Y. Fujiwara, Y. Komohara, E. Araki, N. Kubota, Orally administrated acetate inhibits atherosclerosis progression through AMPK activation via GPR43 in plaque macrophages. Atherosclerosis 401, 119088 (2025). 10.1016/j.atherosclerosis.2024.119088 39705906

[57] E. R. Hutchison, J. Mallikarjun, J. H. Byun, L. N. Lucas, K. Kasahara, M. Tallon, Q. Zhang, D. Amador-Noguez, Y. Liu, V. A. Leone, B. W. Parks, F. E. Rey, GPR41 deficiency alters the gut microbiota-bile acid axis, reduces ileal expression of *Npc1l1*, and attenuates hypercholesterolemia in male mice. Gut Microbes 17, 2598957 (2025). 10.1080/19490976.2025.2598957 PMC12710913 41395766

[58] X. Chen, X. Tang, Y. Li, J. He, Imidazole propionate: Cause and cure in atherosclerosis? Cell Metab. 37, 1929–1931 (2025). 10.1016/j.cmet.2025.08.006 41061667

[59] X. Yuan, L. Wang, O. M. Bhat, H. Lohner, P. L. Li, Differential effects of short chain fatty acids on endothelial Nlrp3 inflammasome activation and neointima formation: Antioxidant action of butyrate. Redox Biol. 16, 21–31 (2018). 10.1016/j.redox.2018.02.007 PMC5842312 29475132

