# Supplementary material for: Engineered synbiotic microspheres enable system-level reprogramming of the gut-immune-vascular axis in chronic inflammatory vascular disease
Source: Sci Adv. 2026 Nov 4;12(45):eaeg1718. doi: 10.1126/sciadv.aeg1718 (PMC13733895; doi:10.1126/sciadv.aeg1718)
Supplement: Supplementary file 1 [file sciadv.aeg1718_sm.pdf]

Science Advances
AAAS

Supplementary Materials for

Engineered synbiotic microspheres enable system-level reprogramming of the gut-immune-vascular axis in chronic inflammatory vascular disease

Lin Shen *et al.*

Corresponding author: Jiansong Ji, jjstcty@wmu.edu.cn; Minjiang Chen, minjiangchenv@163.com; Chenying Lu, luchenying@zju.edu.cn; Weiqian Chen, ls2119088@126.com

Sci. Adv. **12**, eaeg1718 (2026)
DOI: 10.1126/sciadv.aeg1718

This PDF file includes:

Figs. S1 to S56
Tables S1 to S3

Overview

This Supplementary Information provides additional experimental procedures, methodological details, supplementary figures, and supporting data that complement the findings presented in the main manuscript. The supplementary materials include additional validation experiments, quantitative analyses, and mechanistic evidence supporting the fabrication, characterization, biological performance, intestinal targeting, microbiota modulation, and therapeutic efficacy of the engineered synbiotic microsphere platform.

Contents

- Supplementary Figures (Figs. S1–S56 and Tables S1-S3)
- Supplementary References

Supplementary Figures

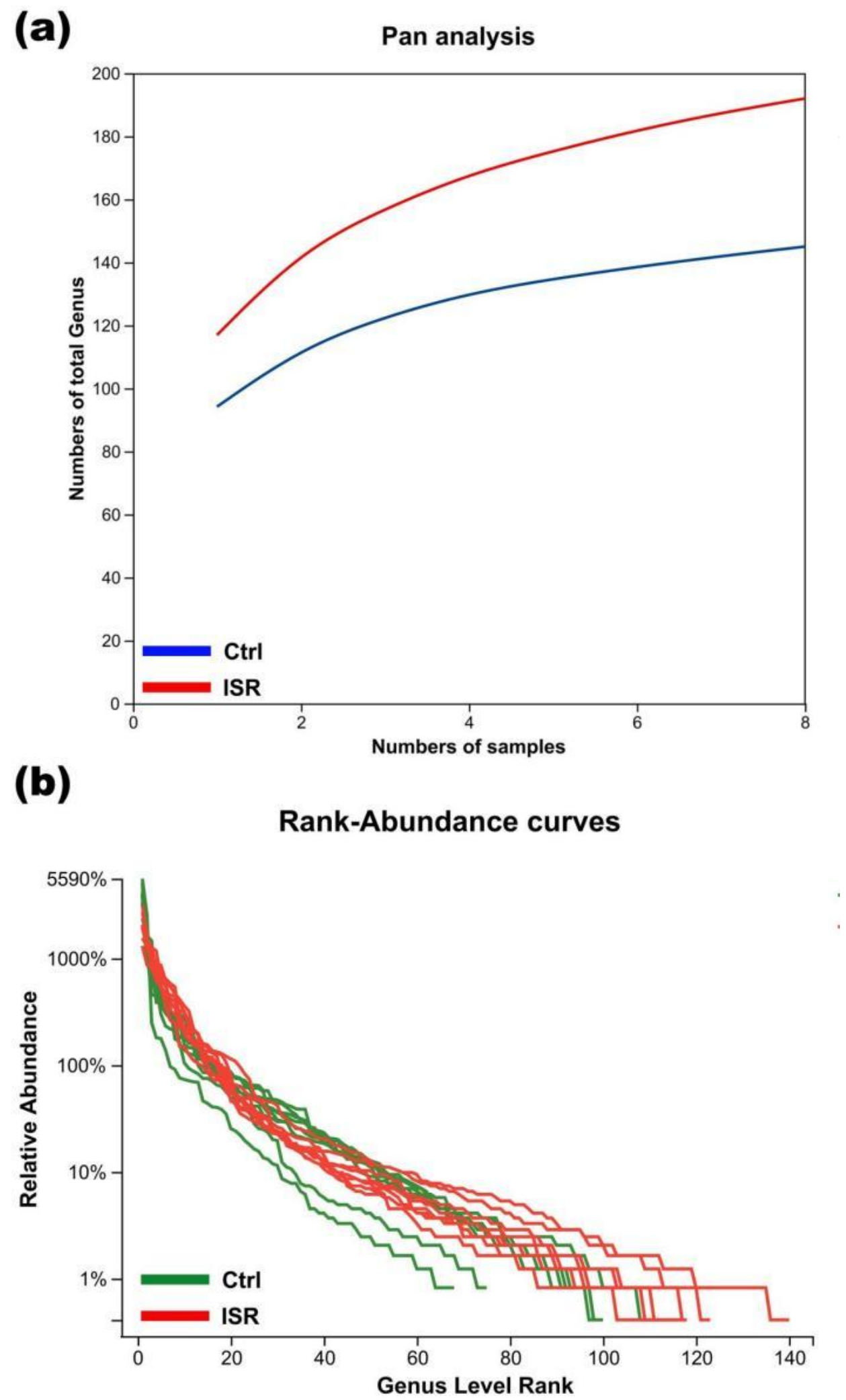

Fig. S1 Pangenome and rank abundance profiles of gut microbiota. (a) Pangenome curves of genus accumulation in Ctrl and in-stent restenosis (ISR) groups. **(b)** Genus-level rank abundance distributions for Ctrl and ISR samples.

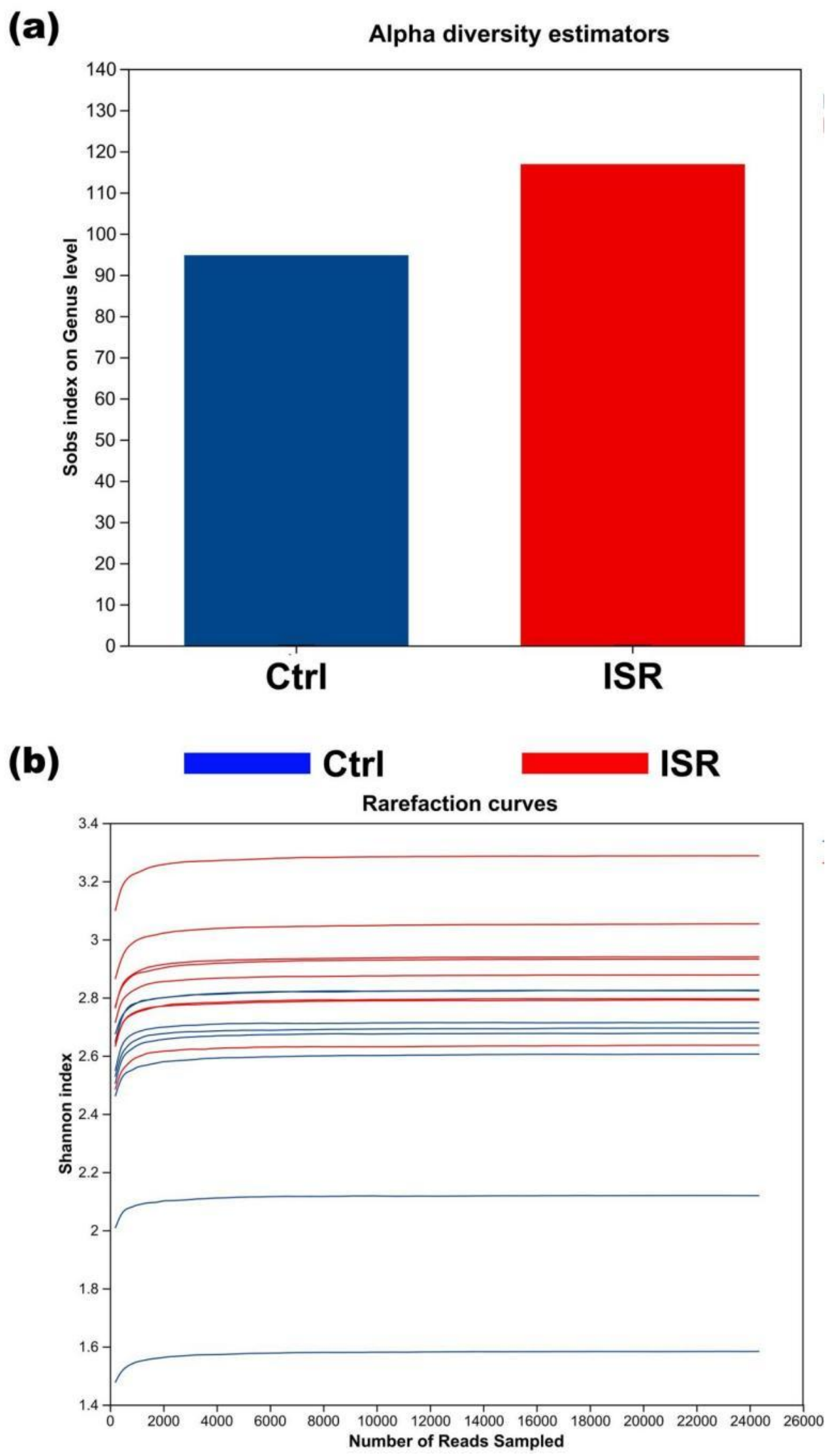

Fig. S2 Alpha diversity features of the gut microbiota in Ctrl and in-stent restenosis (ISR) groups. (a) Genus-level Sobs index for alpha diversity comparison. **(b)** Shannon-based rarefaction curves for Ctrl and ISR samples.

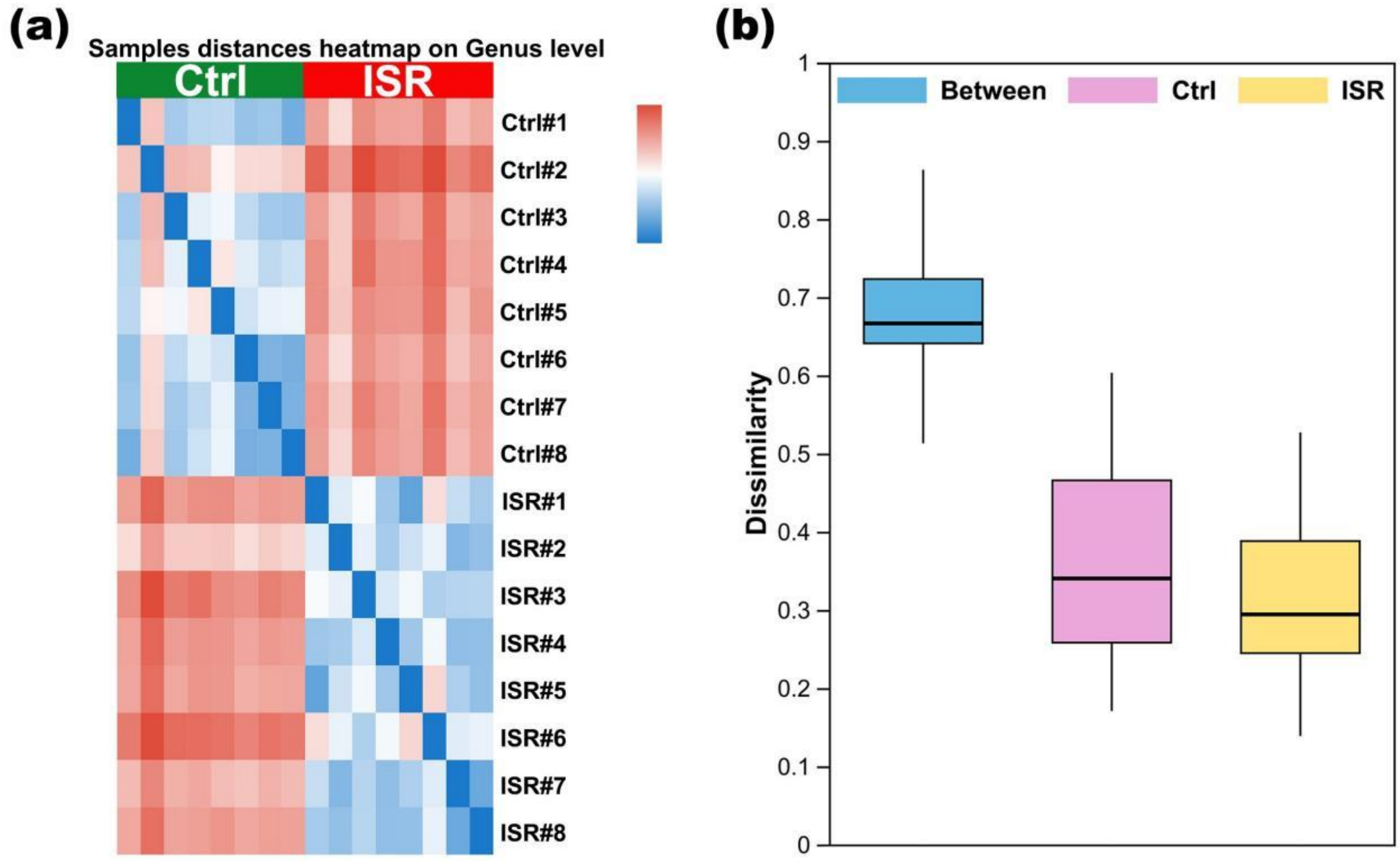

Fig. S3 Sample-level dissimilarity of gut microbiota between Ctrl and in-stent restenosis (ISR) groups. (a) Heatmap of genus-level pairwise distances among samples from Ctrl and ISR groups. **(b)** Boxplots of within-group and between-group dissimilarity.

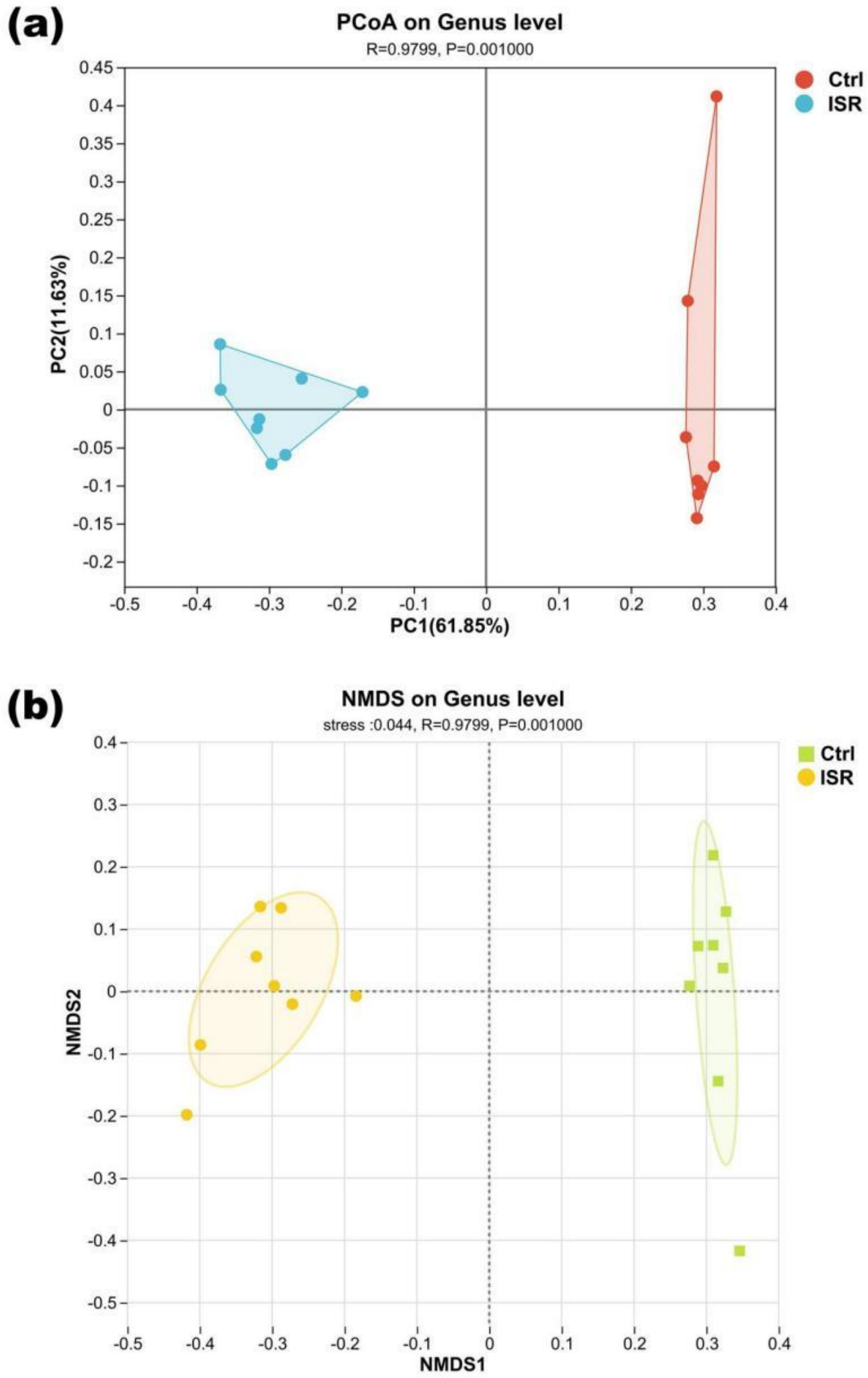

Fig. S4 β-diversity comparison of gut microbiota between Ctrl and in-stent restenosis (ISR) groups. (a) Genus-level principal coordinate analysis ordination of microbial communities in Ctrl and ISR samples. (b) Genus-level non-metric multidimensional scaling ordination illustrating community separation between groups.

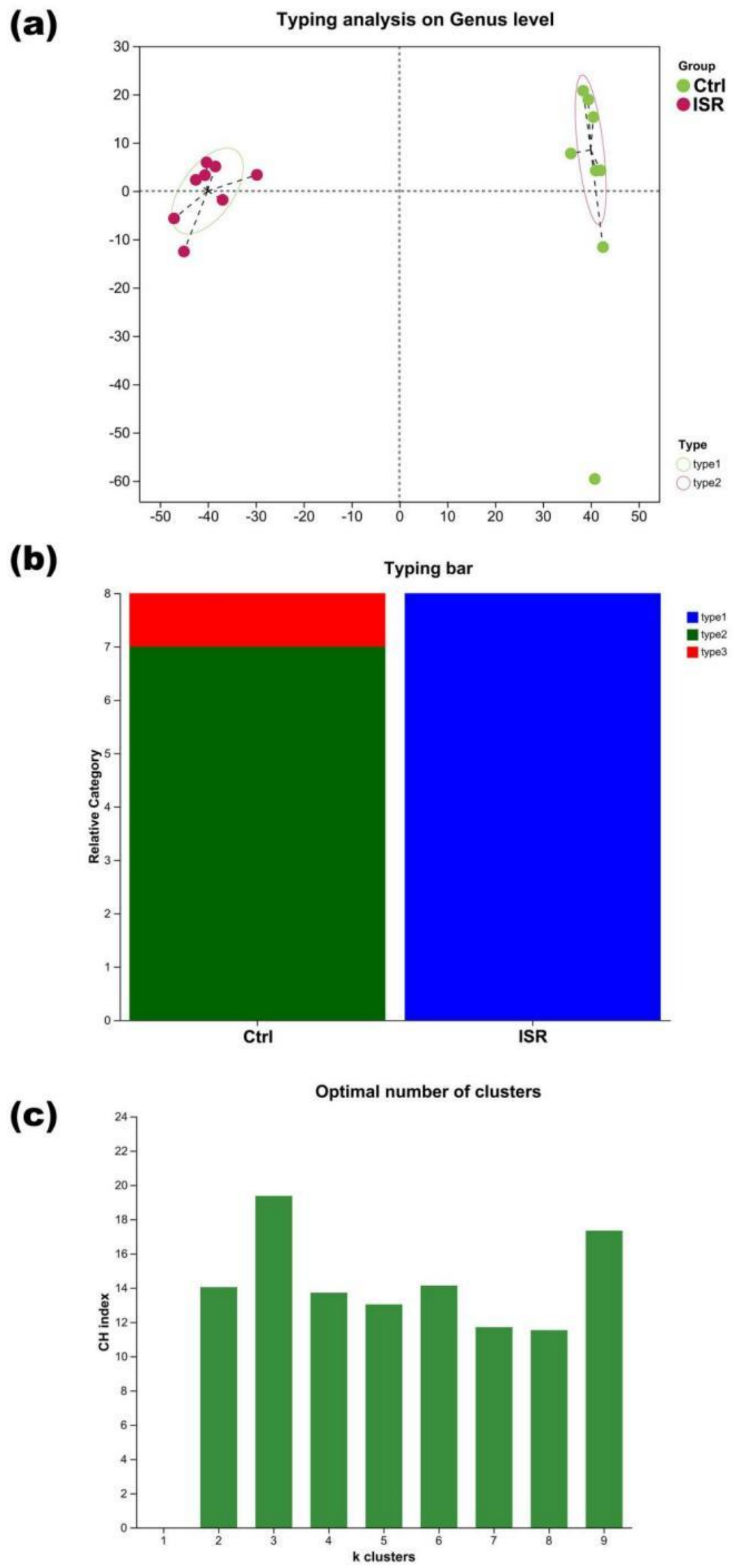

Fig. S5 Genus-level community typing analysis of Ctrl and in-stent restenosis (ISR) groups. (a) Genus-level typing ordination of gut microbial communities. **(b)** Stack bar plot of typing categories in Ctrl and ISR groups. **(c)** CH index-based estimation of the optimal cluster number.

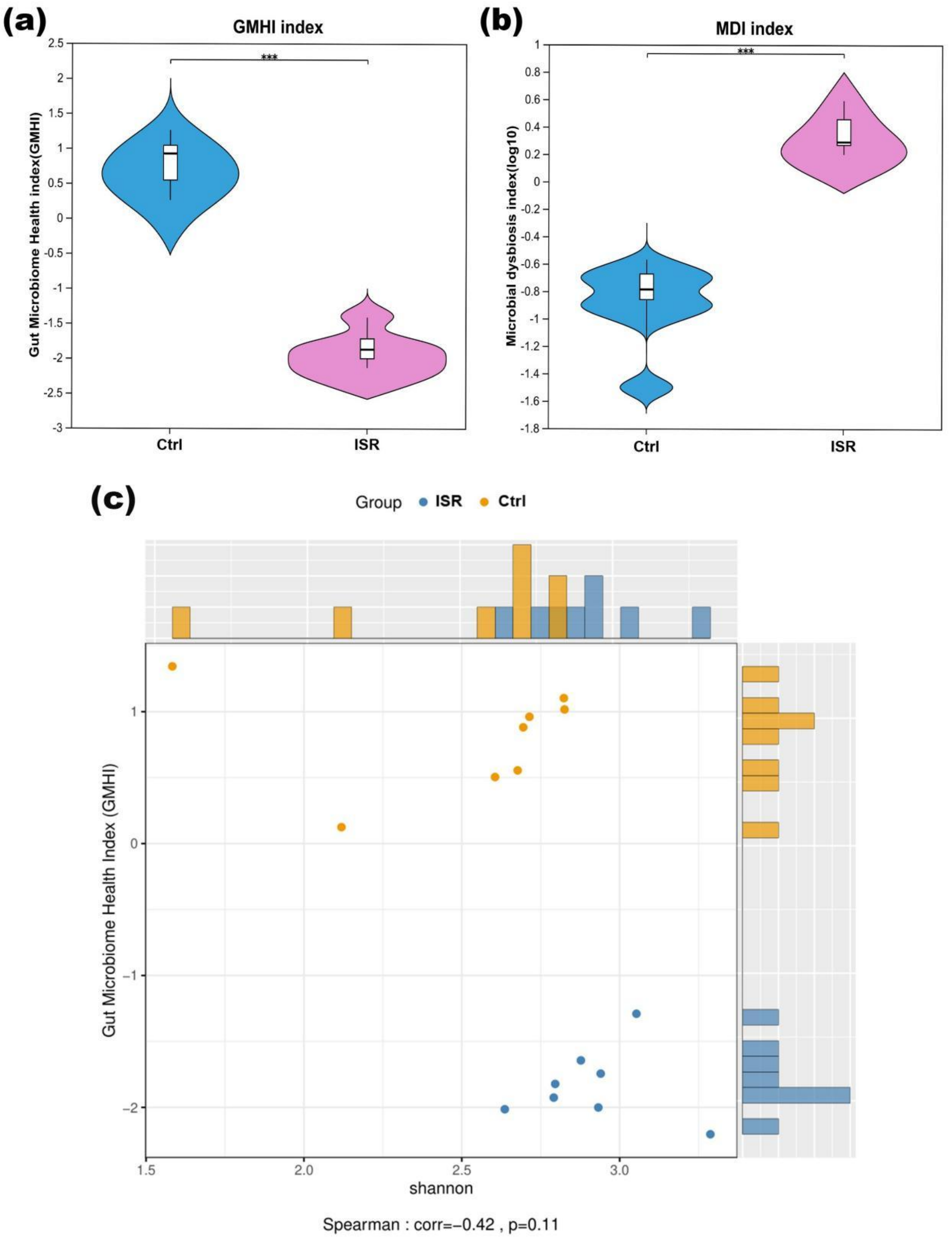

Fig. S6 Gut microbiome health and dysbiosis indices in Ctrl and in-stent restenosis (ISR) groups. (a) Distribution of the gut microbiome health index (GMHI) in Ctrl and ISR samples. **(b)** Distribution of the microbial dysbiosis index in Ctrl and ISR samples. **(c)** Scatterplot of Shannon diversity versus GMHI with marginal histograms and Spearman correlation.

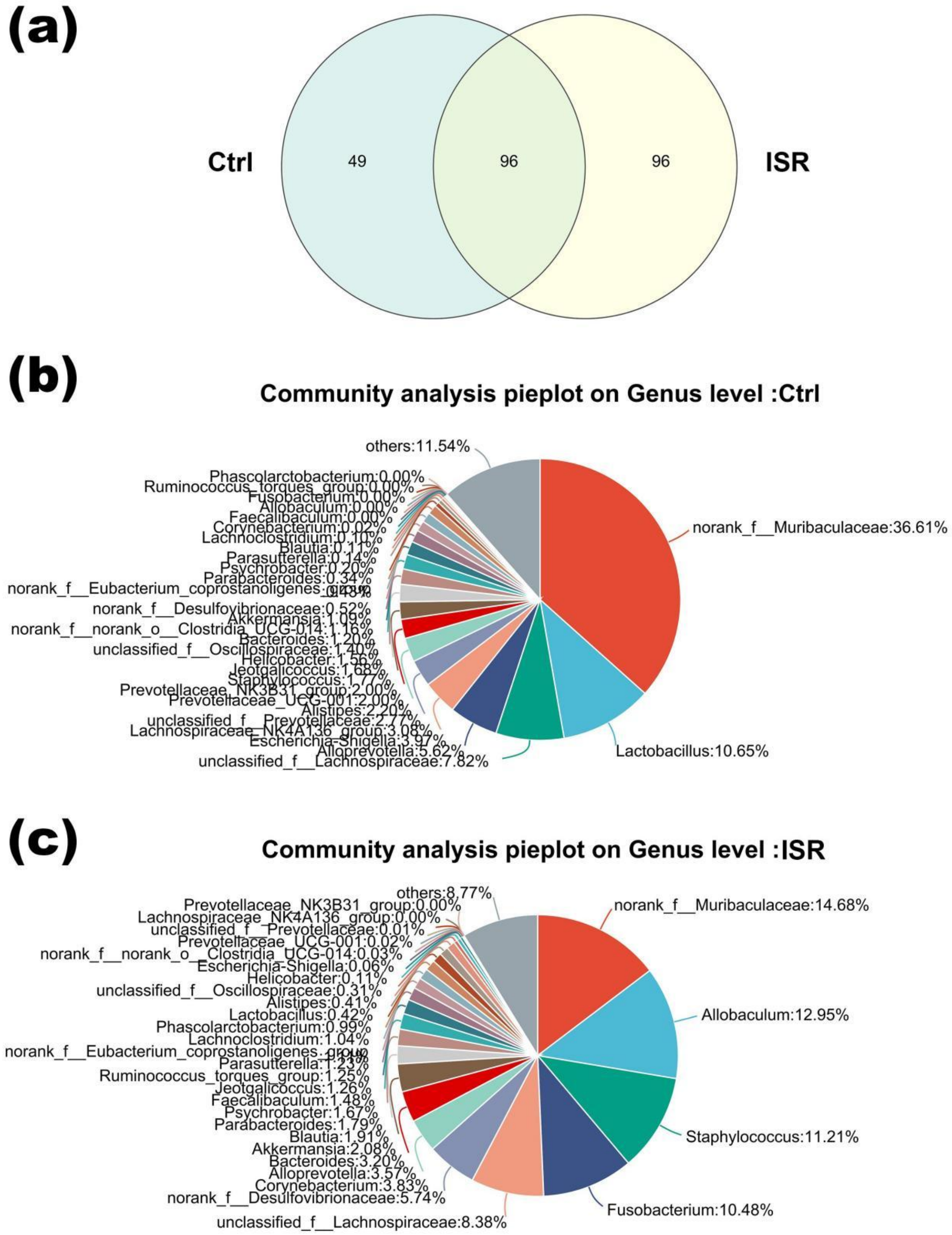

Fig. S7 Genus-level community composition of the gut microbiota in Ctrl and in-stent restenosis (ISR) groups. **(a)** Venn diagram of shared and unique genus-level operational taxonomic units between Ctrl and ISR groups. **(b, c)** Genus-level community composition in the Ctrl and ISR groups.

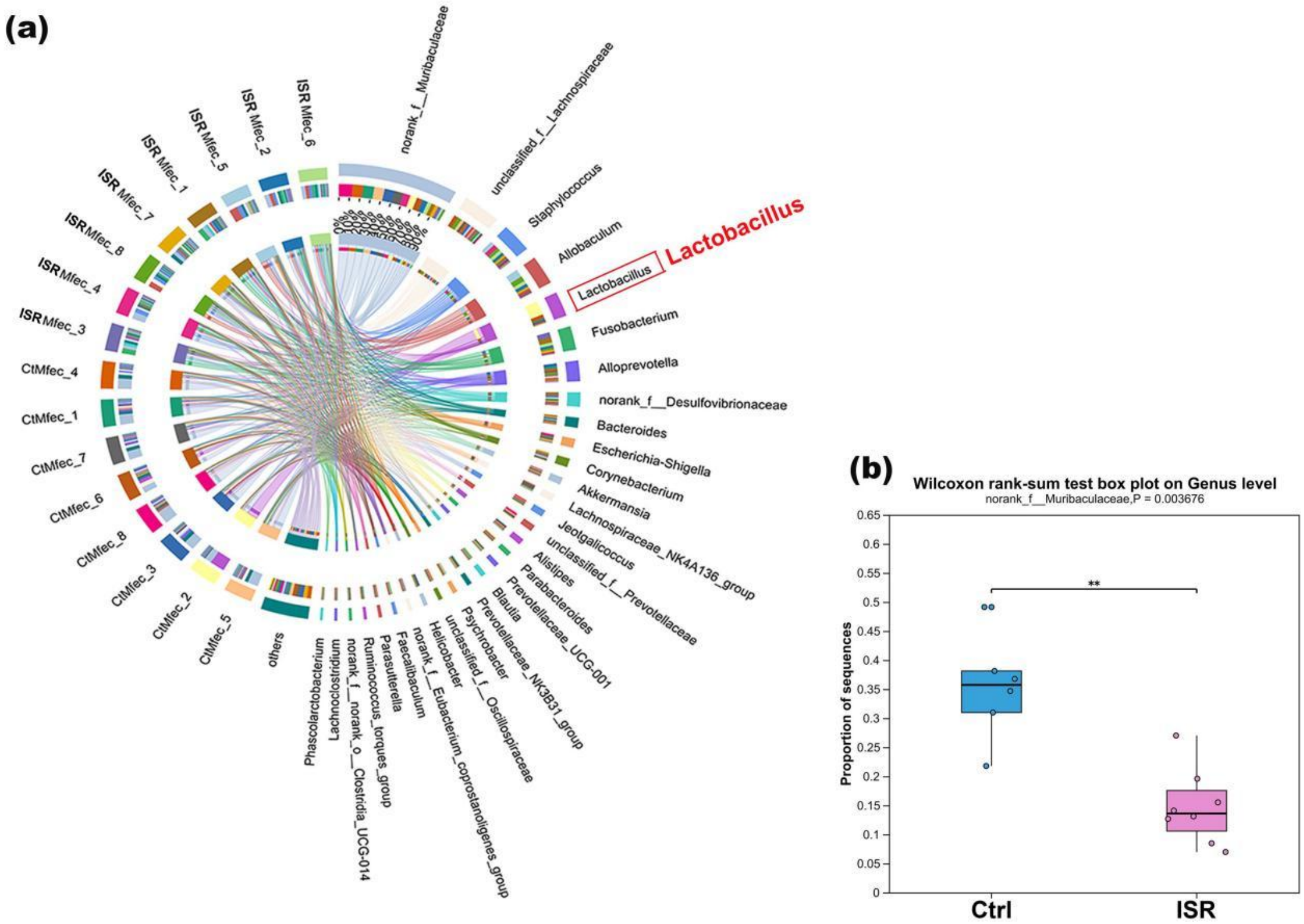

Fig. S8 Genus-level association network and differential abundance in Ctrl and in-stent restenosis (ISR) groups. (a) Circular association plot of genus-level microbial communities. **(b)** Boxplot of *norank f Muribaculaceae* relative abundance based on the Wilcoxon rank-sum test.

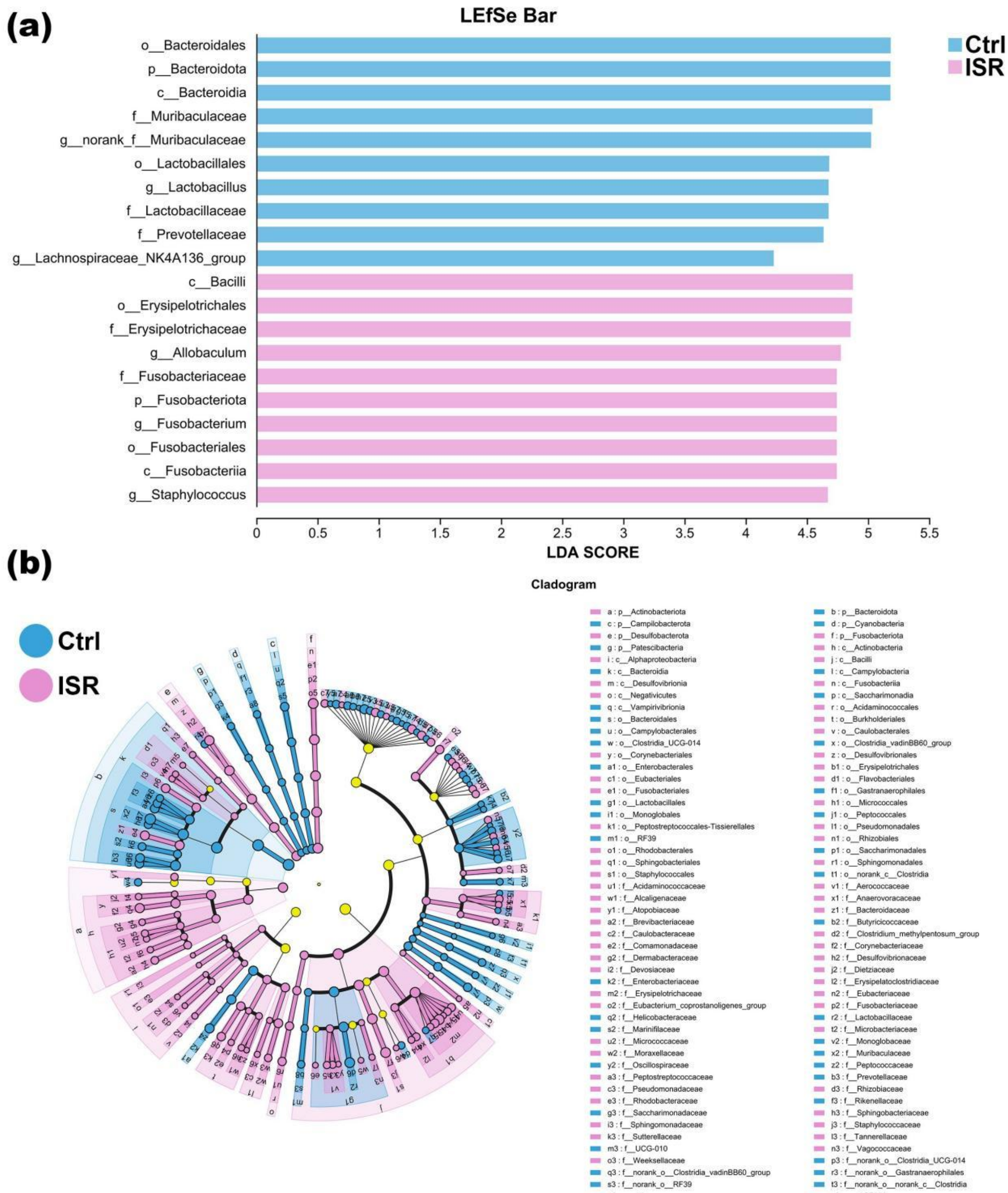

Fig. S9 Differential bacterial taxa between Ctrl and in-stent restenosis groups identified by linear discriminant analysis effect size (LEfSe). (a) LDA score bar plot of significantly enriched taxa. **(b)** LEfSe cladogram depicting group-specific differential taxa.

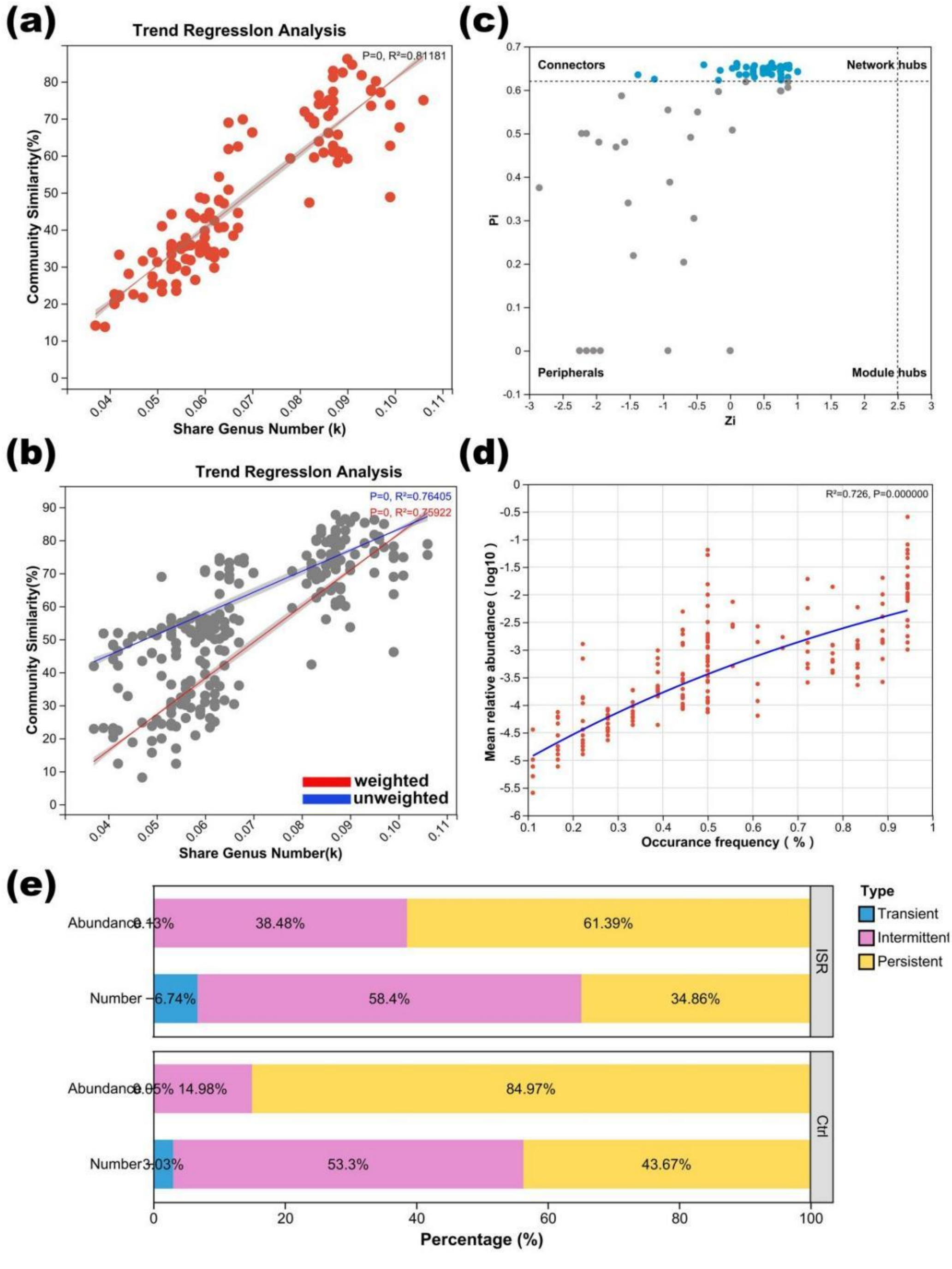

Fig. S10 Trend, network topology, and stability analyses of gut microbial communities in Ctrl and in-stent restenosis (ISR) groups. (a, b) Trend regression analyses of shared genera versus community similarity (weighted and unweighted models). **(c)** Zi-Pi scatterplot of microbial network topological roles. **(d)** Relationship between occurrence frequency and mean relative abundance. **(e)** Proportions of transient, intermittent, and persistent taxa in Ctrl and ISR groups.

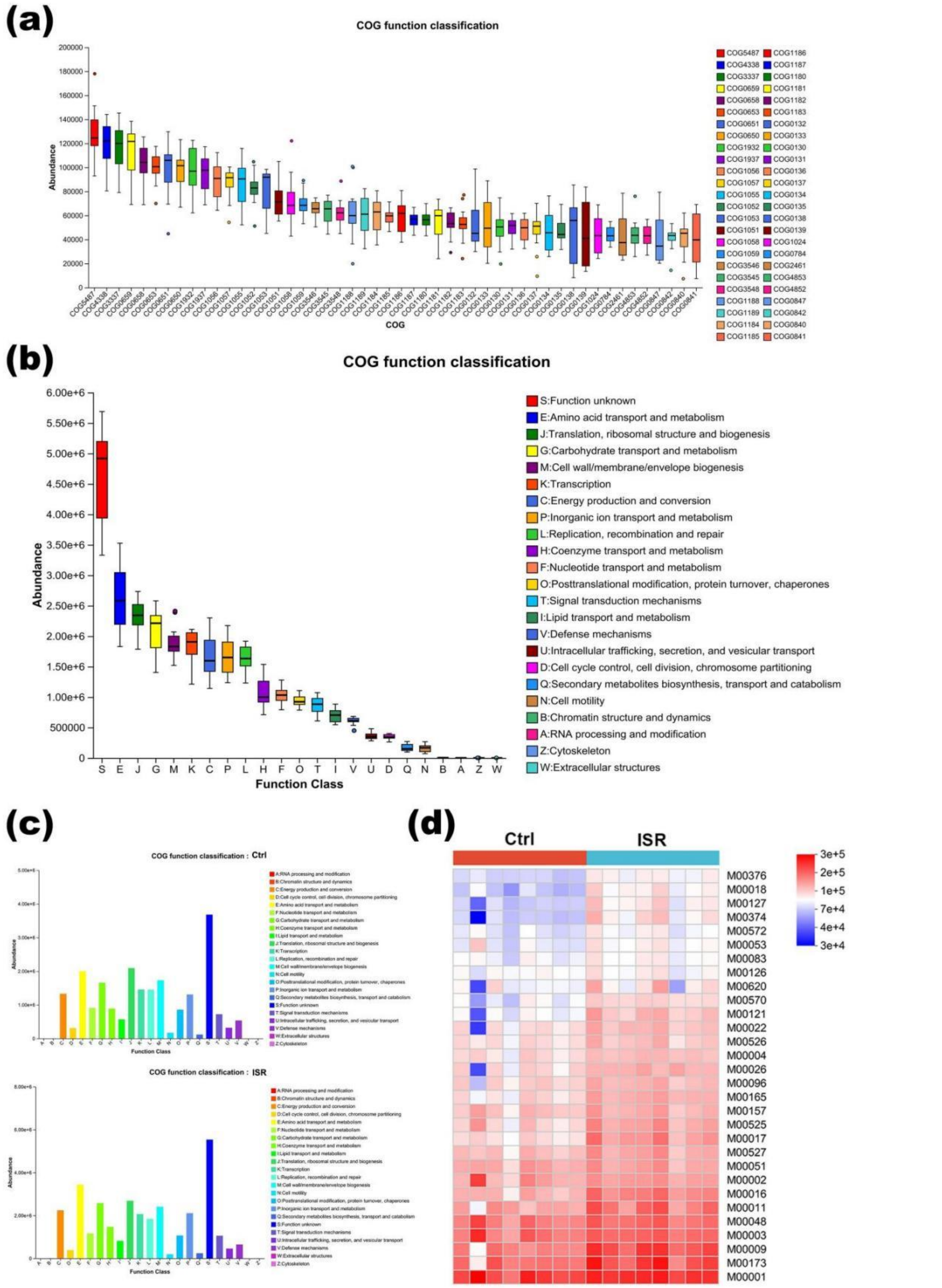

Fig. S11 Cluster of Orthologous Groups (COGs) functional classification of gut microbiota in Ctrl and in-stent restenosis (ISR) groups. (a) Boxplots of COG functional category abundance. **(b)** Abundance distribution of major COG functional classes. **(c)** Bar plots of COG functional profiles in Ctrl and ISR groups. **(d)** Heatmap

of COG functional features in Ctrl and ISR samples.

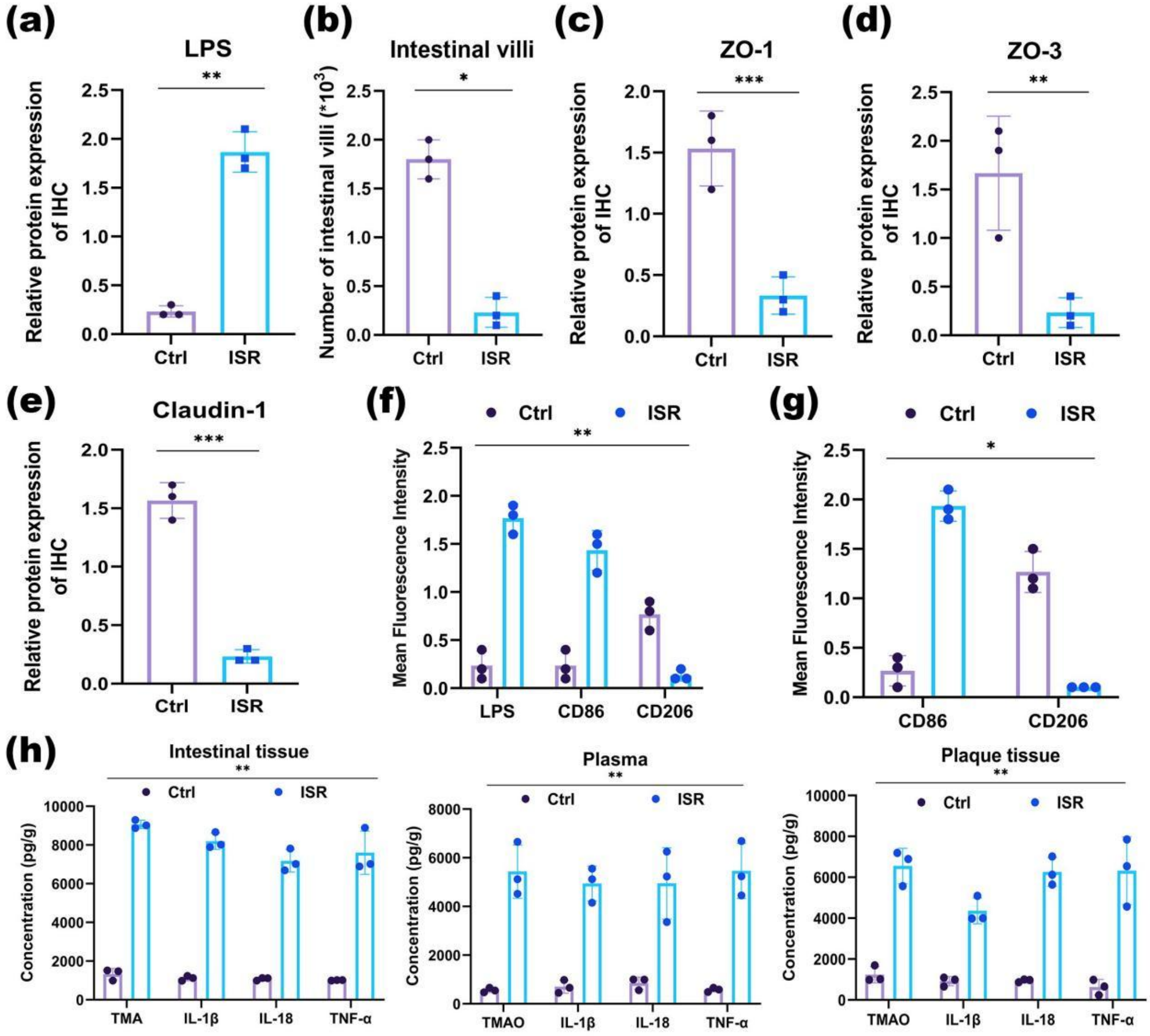

Fig. S12 Quantitative analyses of intestinal barrier disruption, immune imbalance, and inflammatory mediators in patients with in-stent restenosis. (a) IHC quantification of lipopolysaccharide (LPS) corresponding to Fig. 1j. **(b-e)** IHC quantification of intestinal villi, ZO-1, ZO-3, and Claudin-1 corresponding to Fig. 1k. **(f)** IF fluorescence quantification of LPS, CD86, and CD206 in intestinal tissues corresponding to Fig. 1l. **(g)** IF fluorescence quantification of CD86 and CD206 in plaque tissues corresponding to Fig. 1m. **(h)** ELISA measurements of TMA(O) and inflammatory cytokines (IL-1β, IL-18, TNF-α) in intestinal tissue, plasma, and plaque tissue. Data are presented as mean ± SEM across three biologically independent samples. Significance: *$P < 0.05$; **$P < 0.01$; ***$P < 0.001$; ****$P < 0.0001$.

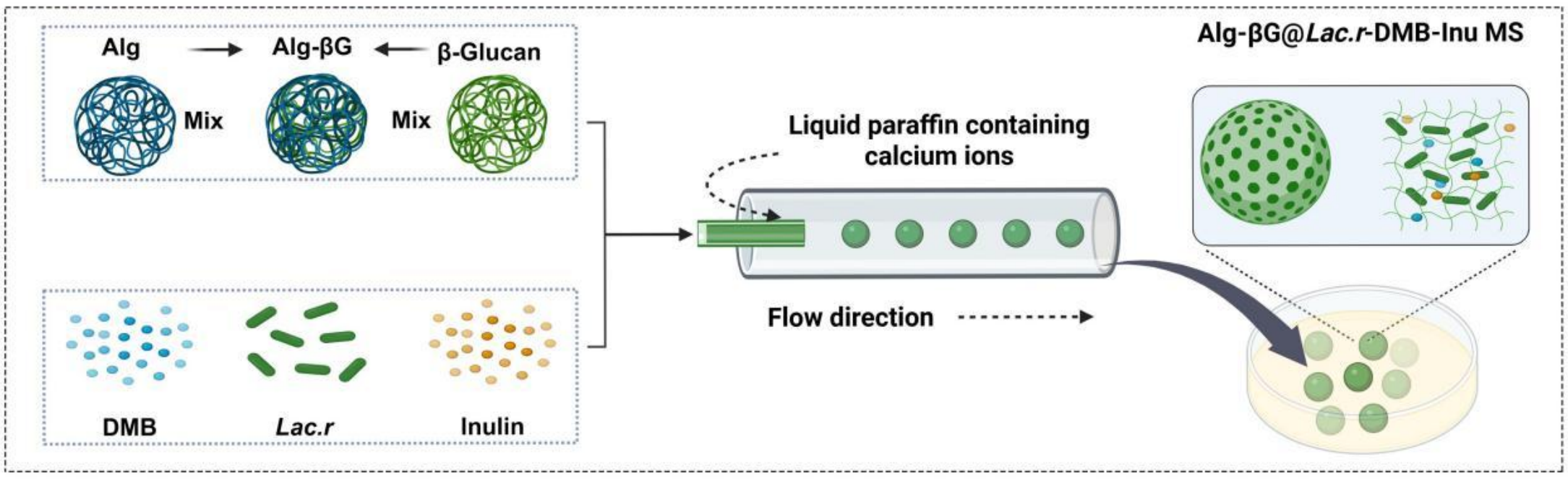

Fig. S13 Microfluidic fabrication of Alg–βG–*Lac.r*–DMB–Inu microspheres. A premixed matrix of alginate (Alg) and β-glucan (βG) was combined with DMB, *Lac.r*, and inulin and injected into a calcium-containing liquid paraffin phase through a microfluidic nozzle. Ionic crosslinking during droplet formation yielded uniform Alg–βG–*Lac.r*–DMB–Inu microspheres encapsulating probiotics and functional components. Created in BioRender. Shen, L. (2026) https://BioRender.com/rtoybrc.

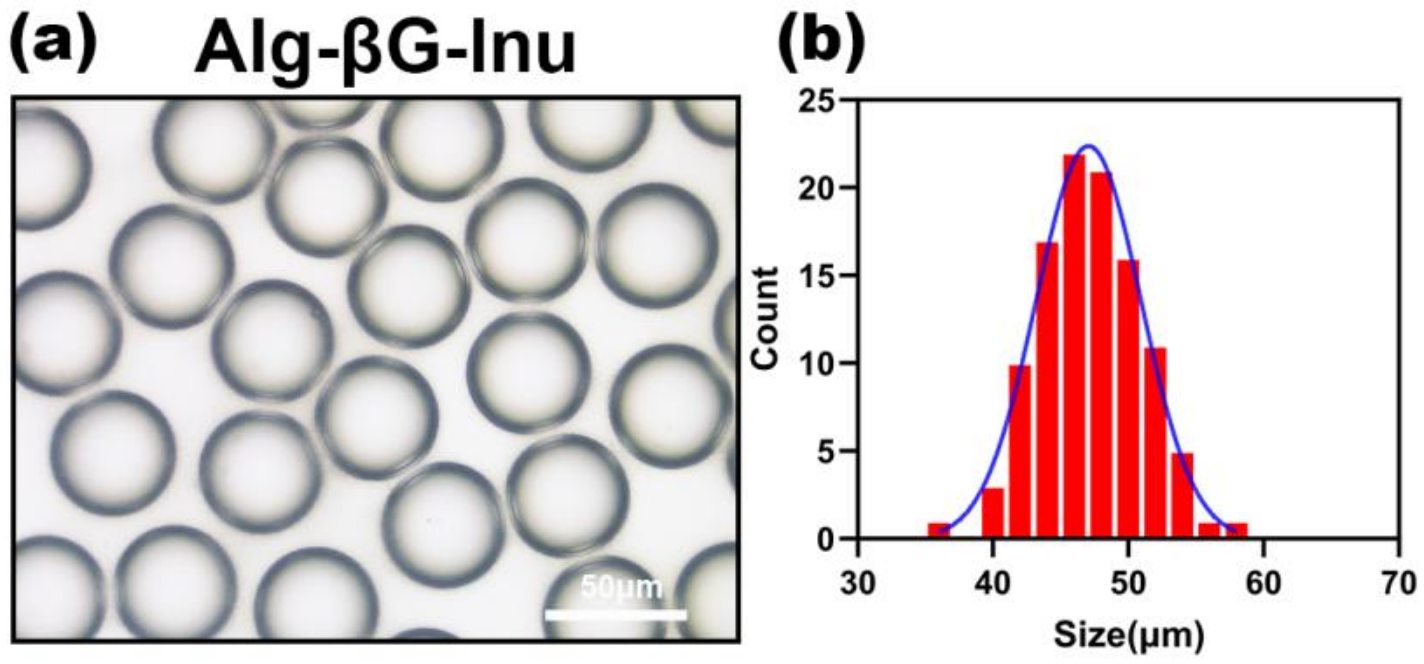

Fig. S14 Quantitative characterization of microsphere monodispersity. (a) Representative bright-field image of Alg–βG–Inu microspheres. Scale bar, 50 μm. **(b)** Particle size distribution of microspheres determined by statistical analysis of 100 individual microspheres. The microspheres exhibited a narrow size distribution, confirming their high degree of monodispersity and fabrication uniformity.

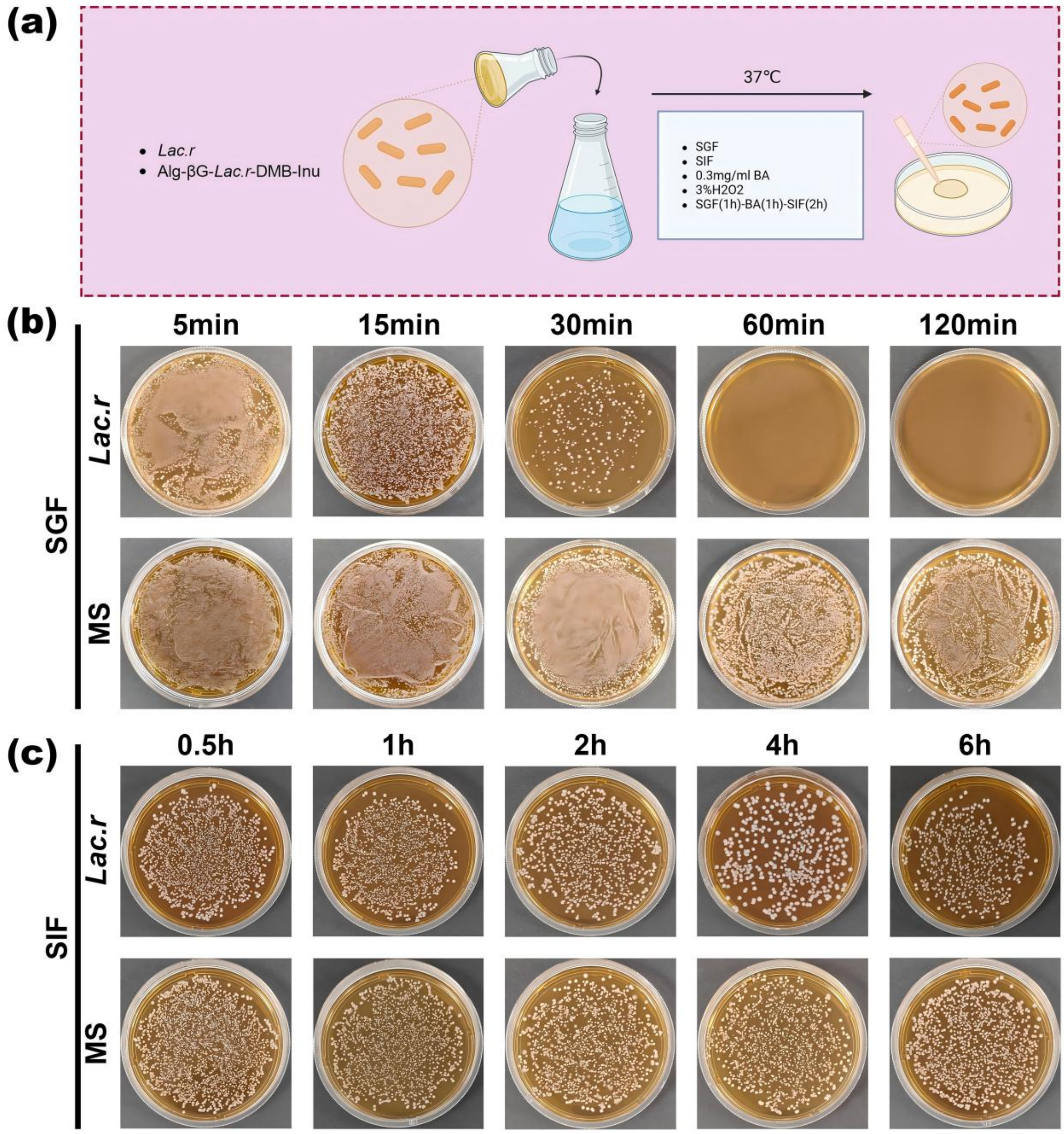

Fig. S15 ***In vitro*** **gastrointestinal tolerance assay of free** ***Lac.r*** **and Alg–βG–*Lac.r*–DMB–Inu microspheres. (a)** Schematic illustration of the experimental workflow for evaluating the gastrointestinal stability of free *Lac.r* and Alg–βG–*Lac.r*–DMB–Inu microspheres. Samples were sequentially exposed to simulated gastric fluid (SGF), simulated intestinal fluid (SIF), 0.3 mg mL^{-1} bile acids, and 3% H_2O_2, followed by plating on MRS agar after incubation at 37 °C. Created with BioRender.com. **(b)** Representative MRS agar plates showing the survival of free *Lac.r* and microspheres after incubation in SGF for 5, 15, 30, 60, and 120 min. **(c)** Representative MRS agar plates of free *Lac.r* and microspheres following exposure to

SIF for 0.5, 1, 2, 4, and 6 h. Created in BioRender. Shen, L. (2026) https://BioRender.com/979uwb6.

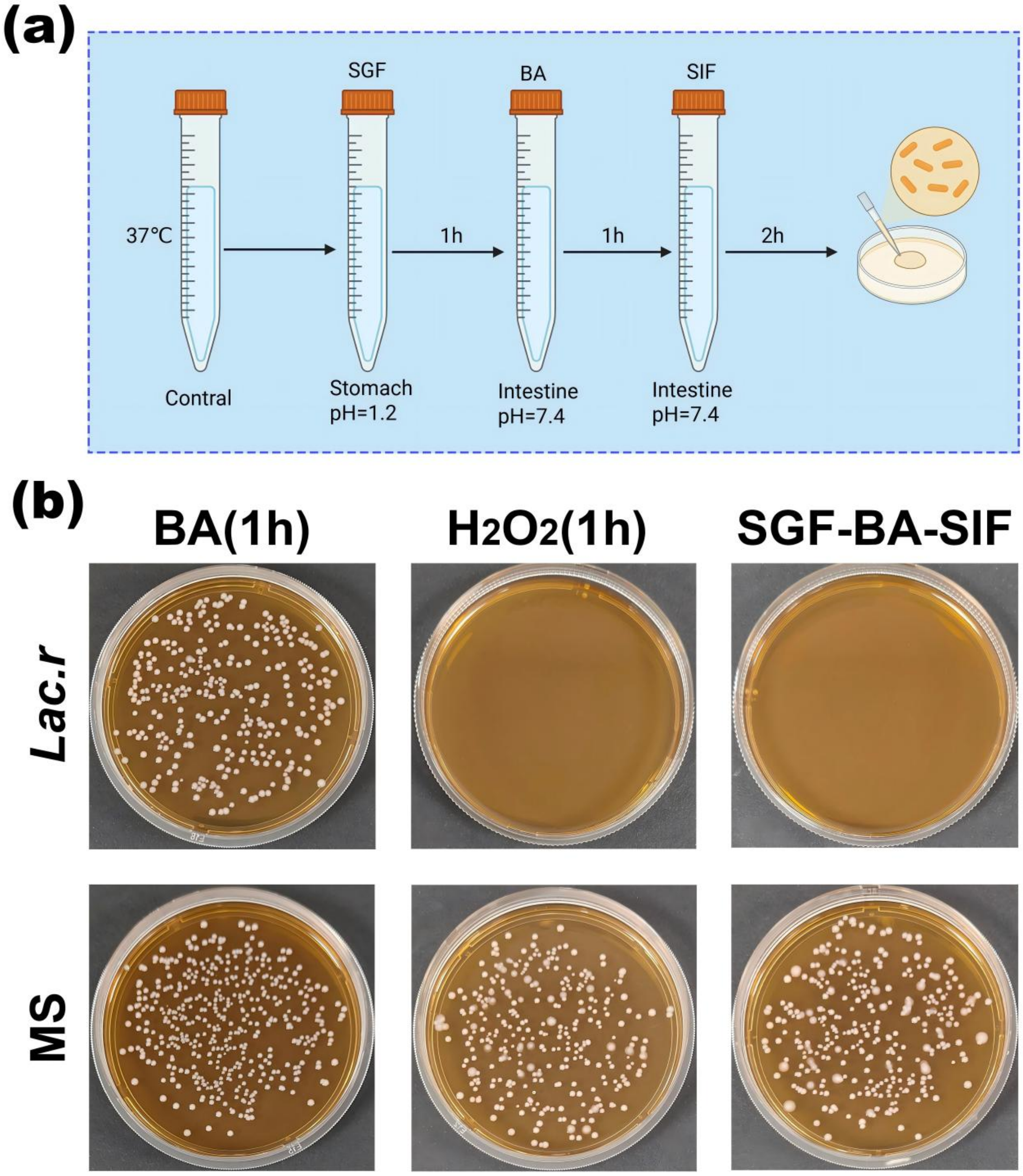

Fig. S16 ***In vitro*** **multistage gastrointestinal challenge of free** ***Lac.r*** **and Alg–βG–*Lac.r*–DMB–Inu microspheres (MS).** **(a)** Schematic workflow of the sequential gastrointestinal simulation. Samples of free *L. reuteri* and Alg–βG–*Lac.r*–DMB–Inu MS were incubated at 37 °C and sequentially exposed to simulated gastric fluid (SGF; pH 1.2, 1 h), bile acids (BA; pH 7.4, 1 h), and simulated intestinal fluid (SIF; pH 7.4, 2 h), followed by plating on MRS agar. Created with BioRender.com. **(b)** Representative MRS agar plates of free *Lac.r* and MS after

exposure to individual stressors—BA (1 h) and H_2O_2 (1 h)—and following the full sequential SGF–BA–SIF treatment. Created in BioRender. Shen, L. (2026) https://BioRender.com/wgmcfdg.

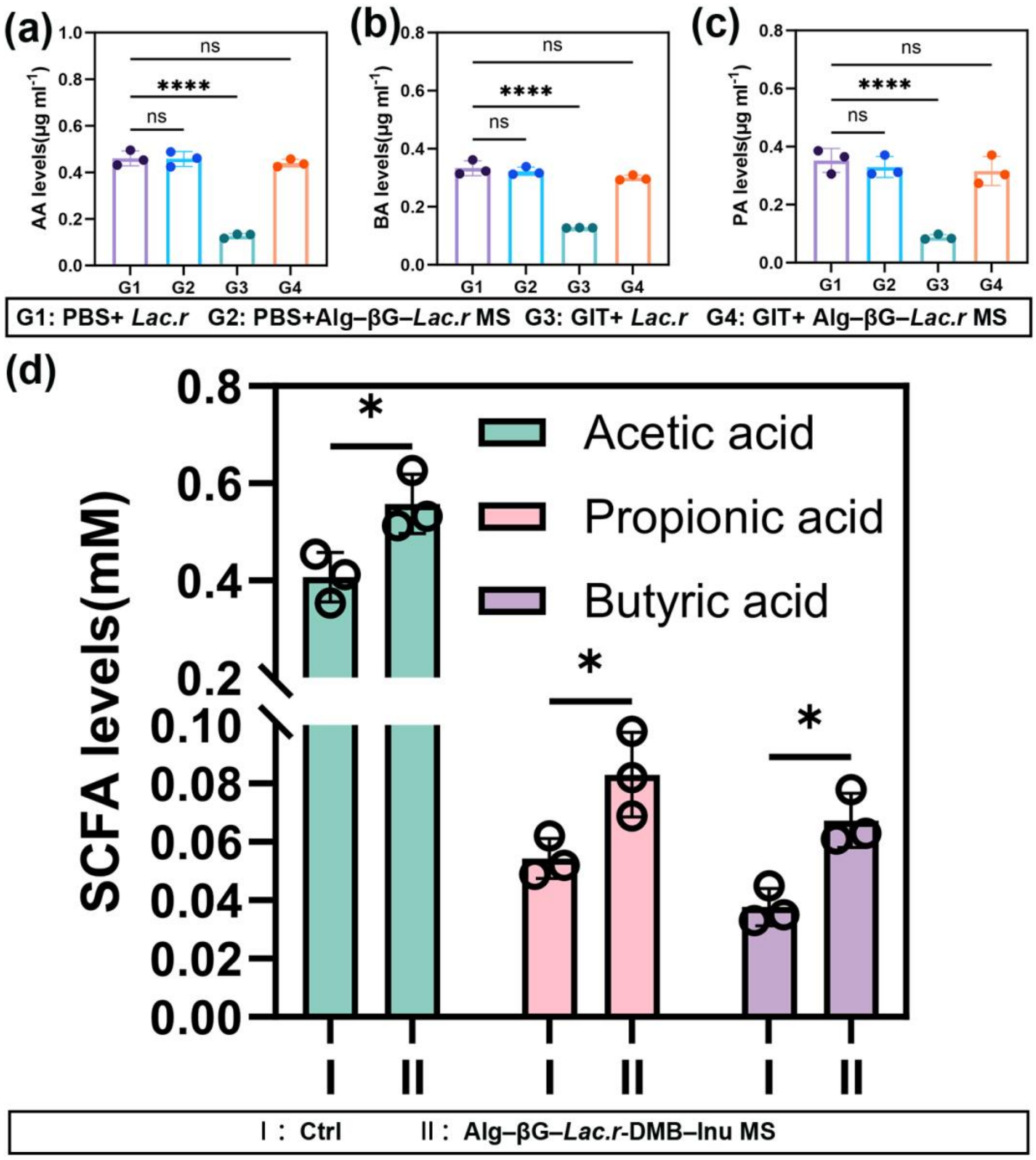

Fig. S17. Alg–βG–*Lac.r* microspheres preserve the metabolic functionality of released *L. reuteri* following simulated gastrointestinal exposure. (a–c) Concentrations of acetic acid (AA), butyric acid (BA), and propionic acid (PA) produced by free or microsphere-encapsulated *L. reuteri* following incubation in phosphate-buffered saline (PBS) or simulated gastrointestinal tract (GIT) fluid. G1, PBS + free *L. reuteri*; G2, PBS + Alg–βG–*Lac.r* microspheres (MS); G3, simulated GIT + free *L. reuteri*; G4, simulated GIT + Alg–βG–*Lac.r* MS. **(d)** Serum concentrations of AA, PA, and BA in mice following treatment. I, Ctrl; II, Alg–βG–*Lac.r*–DMB–Inu MS. Data are presented as mean ± SD. Statistical significance was determined by one-way ANOVA followed by Tukey's

multiple-comparisons test for (a–c) and by a two-tailed Student's t-test for (d). *$P < 0.05$, **$P < 0.01$, ***$P < 0.001$, and ****$P < 0.0001$.

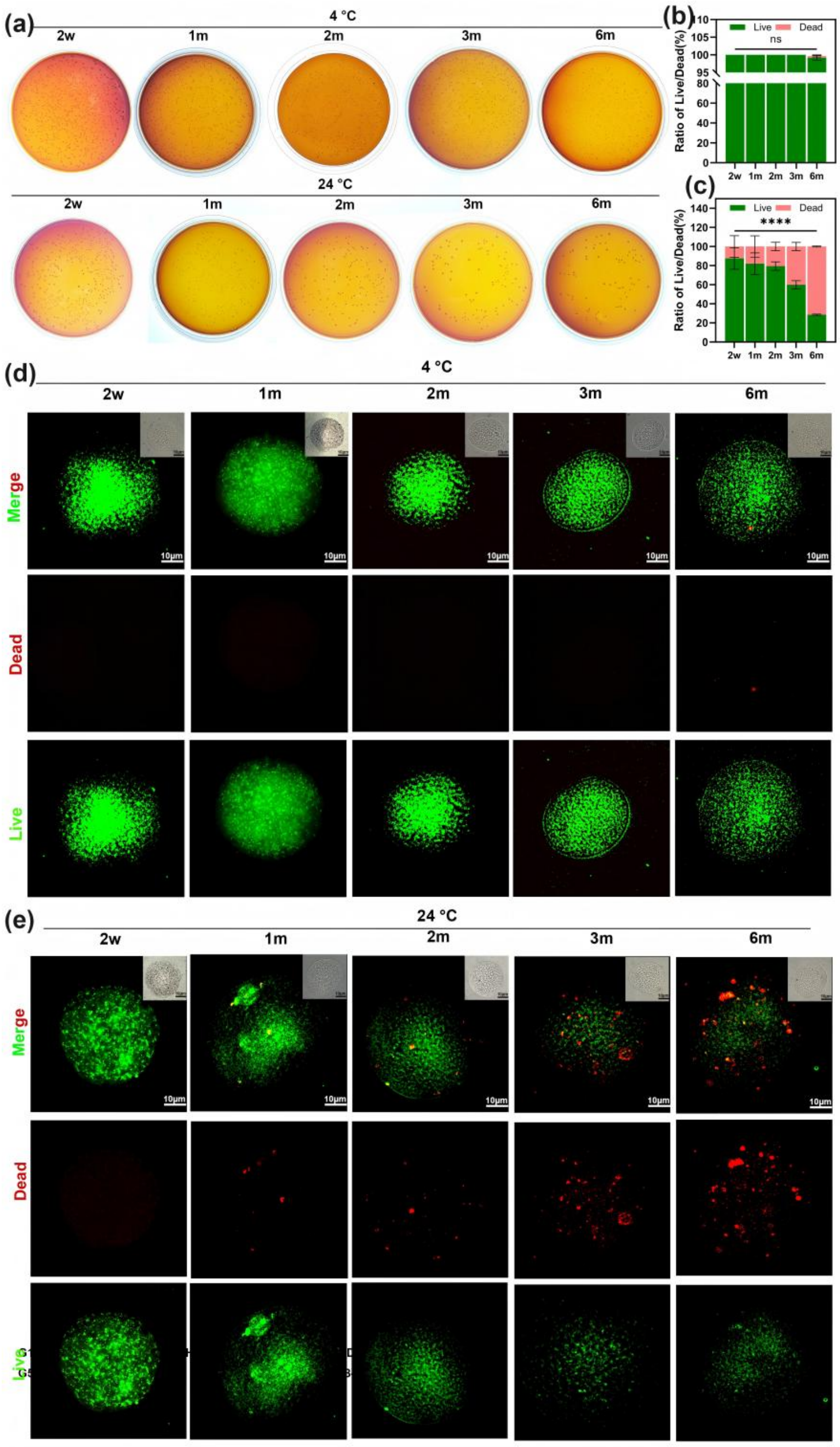

Fig. S18. Long-term viability and survival of encapsulated *L. reuteri* in Alg–βG–*Lac.r*–DMB–Inu microspheres under simulated storage conditions. **(a)** Representative macroscopic images of microspheres stored at 4°C or 24°C for 2 weeks (2w), 1 month (1m), 2 months (2m), 3 months (3m), and 6 months (6m). **(b, c)** Quantification of live/dead bacterial ratios in microspheres following storage at 4°C

(b) and 24 °C (c) (n = 3). **(d, e)** Representative fluorescence images of Live/Dead-stained microspheres after storage at 4 °C (d) and 24 °C (e) for the indicated time points. Live bacteria are shown in green, dead bacteria in red, and merged images are shown in the top row. Insets show the corresponding bright-field images. Scale bars, 10 μm. Data are presented as mean ± SD. Statistical significance was determined by one-way ANOVA followed by Tukey's multiple-comparisons test.$^*P < 0.05$; $^{**}P < 0.01$; $^{***}P < 0.001$; $^{****}P < 0.0001$.; ns, not significant.

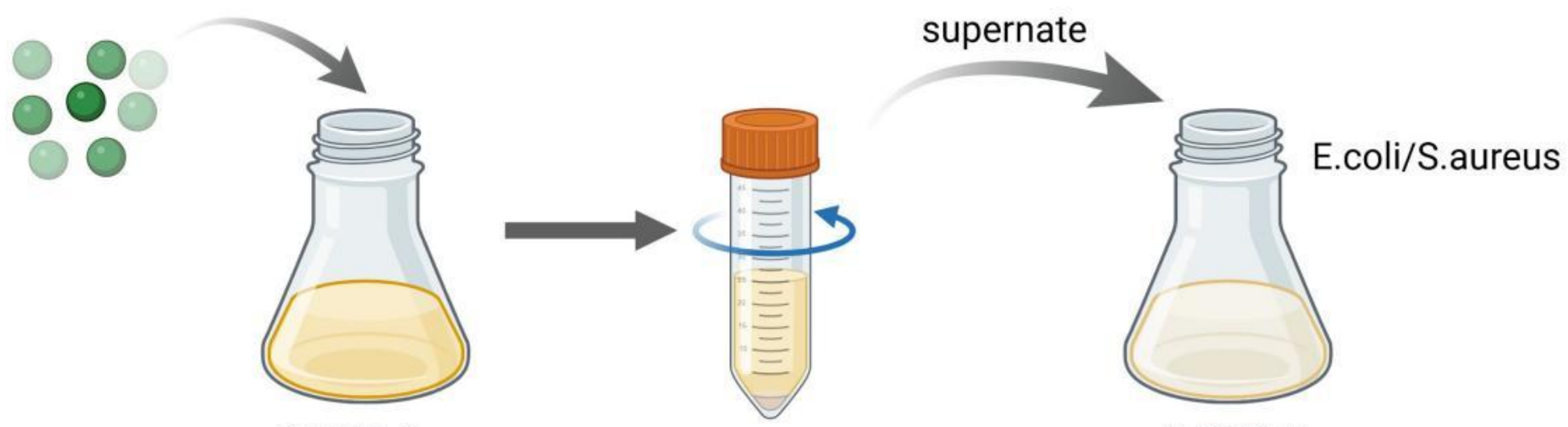

Fig. S19 Schematic workflow for evaluating the inhibitory effects of microsphere leachates on gut pathogens. Created in BioRender. Shen, L. (2026) https://BioRender.com/mliuthe.

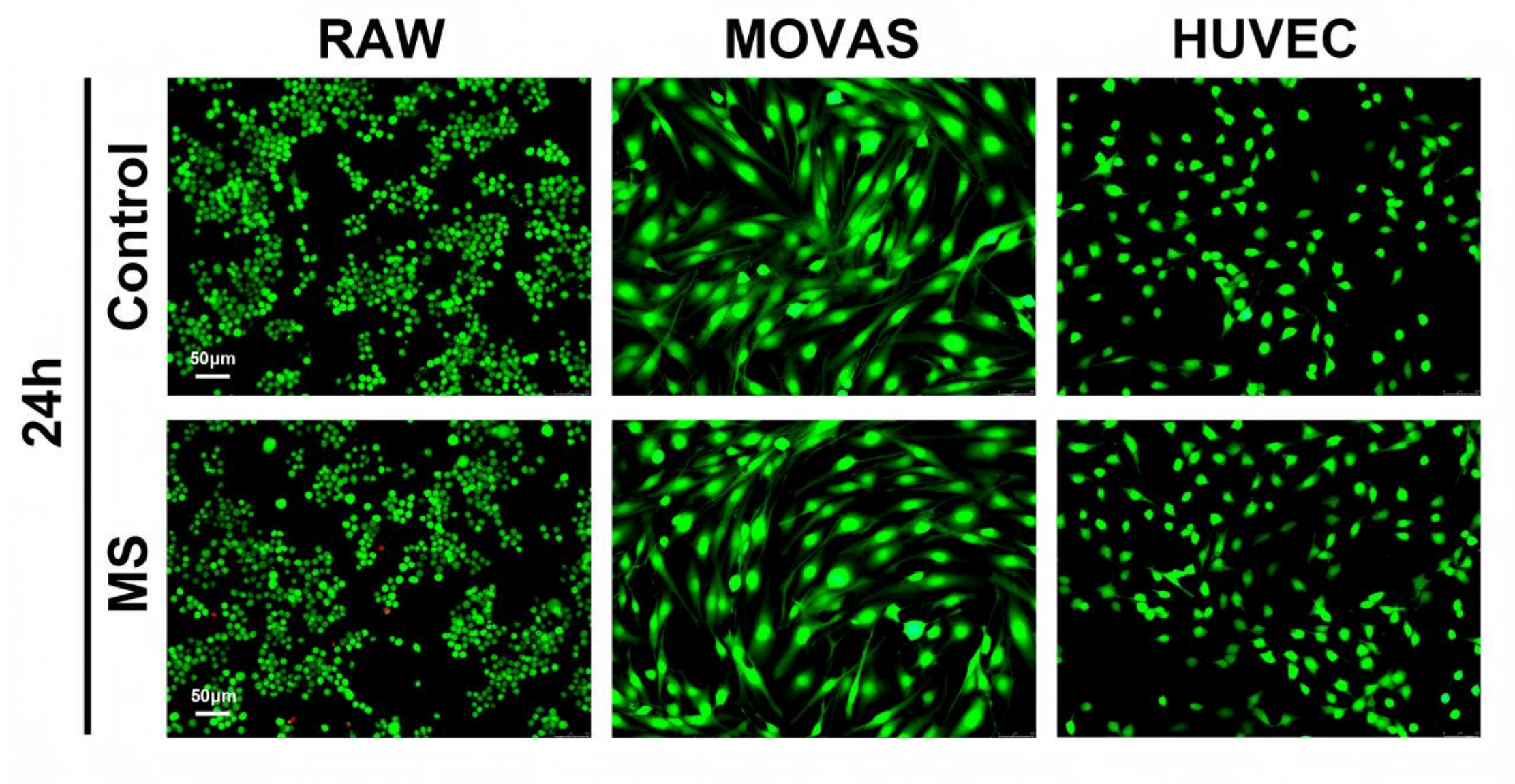

Fig. S20 Fluorescence images of RAW264.7, MOVAS, and HUVEC cells after 24-h incubation with Alg–βG–*Lac.r*–DMB–Inu microspheres or control medium. Calcein-AM (green) and PI (red) staining were used for cell viability visualization.

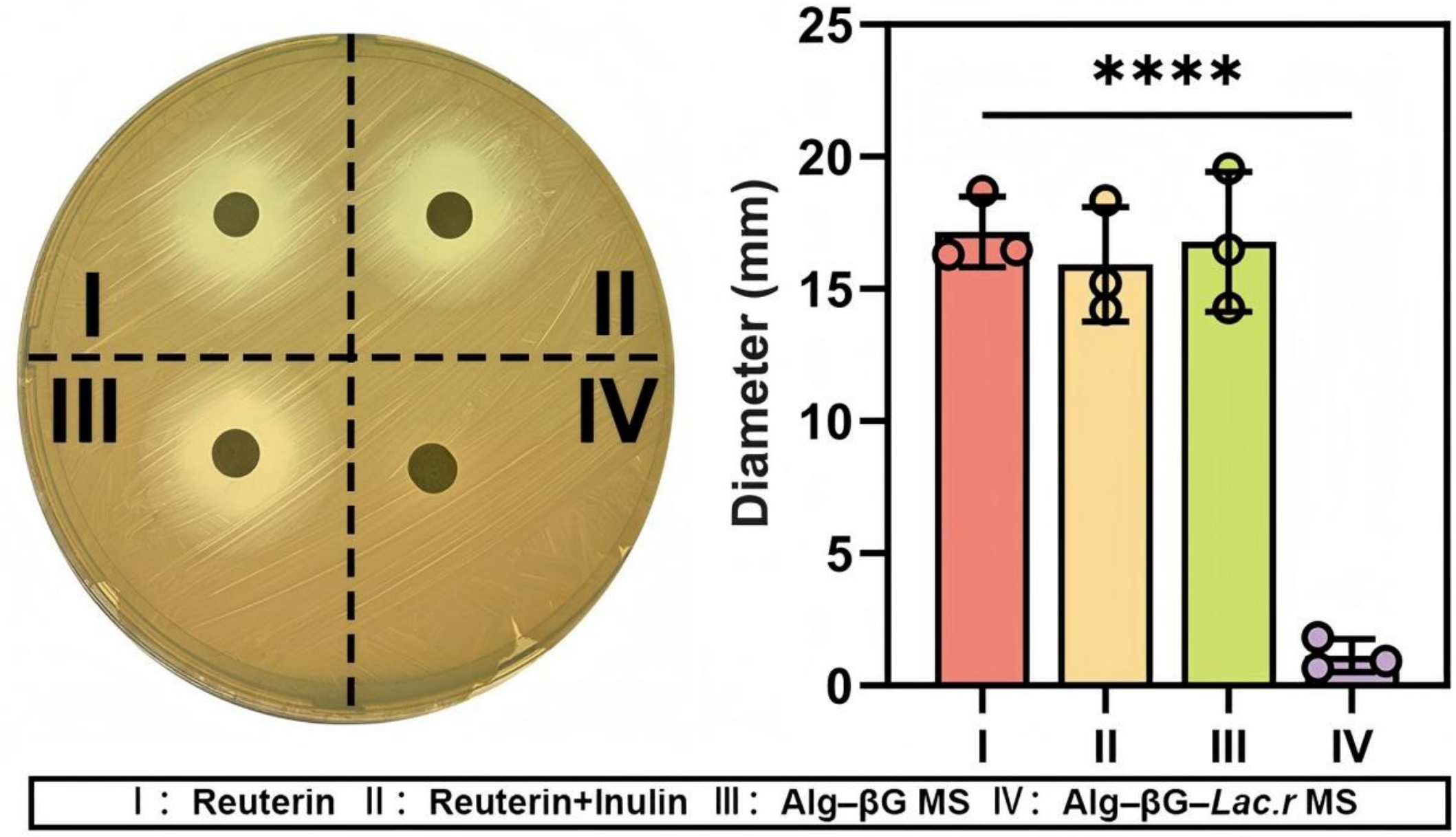

Fig. S21 Identification of the primary source of antibacterial activity in microsphere leachates. (a) Representative images of agar plates showing inhibition zones against indicator bacteria. I: Reuterin; II: Reuterin + inulin; III: Alg-βG MS; IV: Alg-βG-*Lac.r* MS. **(b)** Quantification of inhibition zone diameters (n = 3 biologically independent replicates). Data are presented as means ± SD. Statistical significance was determined by one-way ANOVA with Tukey's multiple comparisons test. ****$P < 0.0001$.

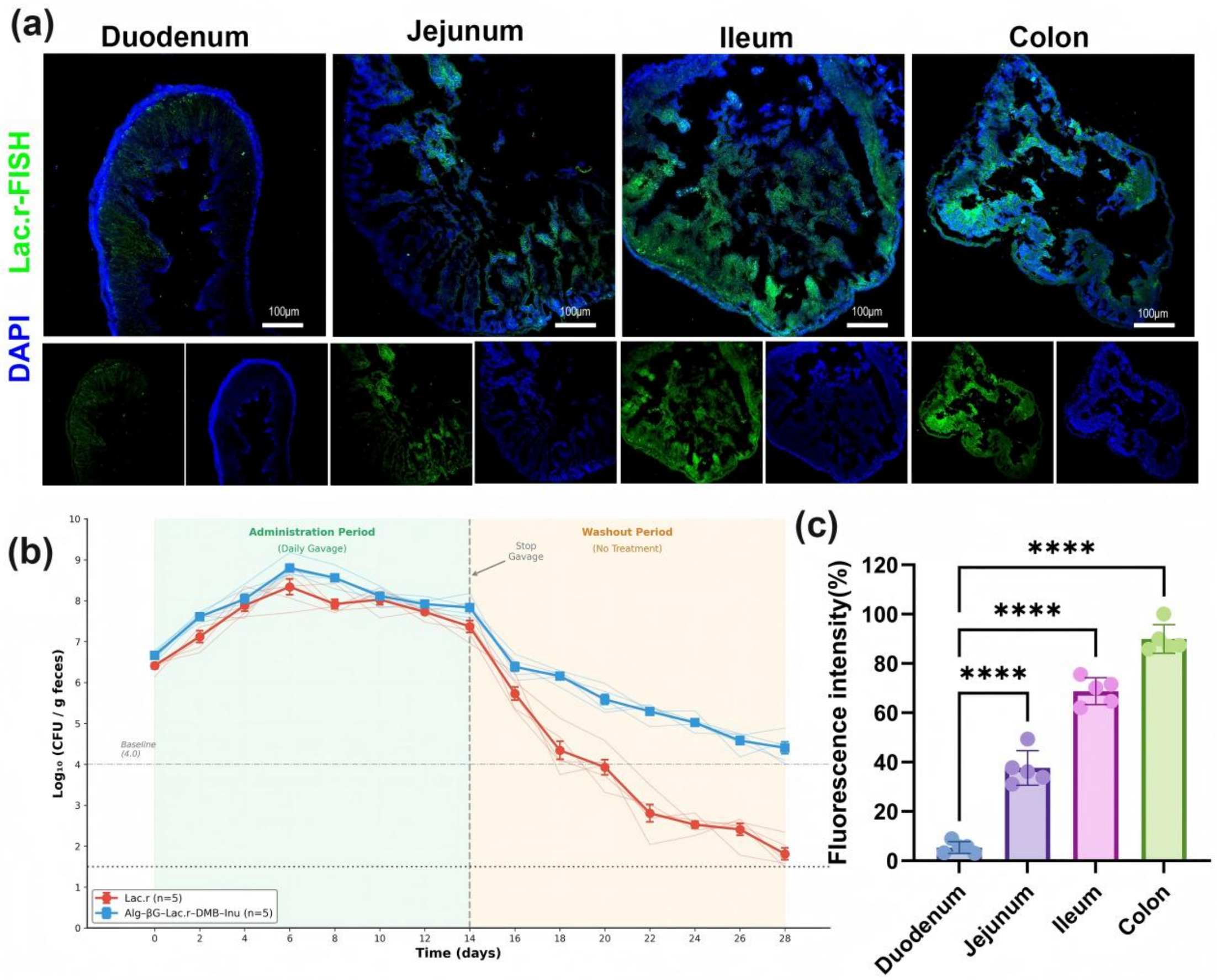

Fig. S22. Alg–βG–*Lac.r*–DMB–Inu microspheres enhance intestinal retention and persistence of Lactobacillus reuteri *in vivo*. (a) Representative fluorescence in situ hybridization (FISH) images of the duodenum, jejunum, ileum, and colon from mice treated with Alg–βG–*Lac.r*–DMB–Inu microspheres. *L. reuteri* was detected using a species-specific FISH probe (green), and nuclei were counterstained with DAPI (blue). Merged images are shown in the upper panels, with the corresponding FISH and DAPI channels displayed below. Scale bars, 100 μm. **(b)** Longitudinal quantification of fecal *L. reuteri* abundance over 28 days, expressed as $\log_{10}$ colony-forming units (CFU) per gram of feces. Mice received daily oral gavage of free *L. reuteri* (red, n = 5) or Alg–βG–*Lac.r*–DMB–Inu microspheres (blue, n = 5) during the administration period (days 0–14, green shaded region), followed by a treatment-free washout period (days 14–28, orange shaded region). **(c)** Quantification of *L. reuteri* FISH fluorescence intensity in different intestinal segments from mice treated with Alg–βG–*Lac.r*–DMB–Inu microspheres (n = 5). Data are presented as mean ± SD.

Statistical significance was determined by one-way ANOVA followed by Tukey's multiple-comparisons test. *$P < 0.05$, **$P < 0.01$, ***$P < 0.001$, and ****$P < 0.0001$.

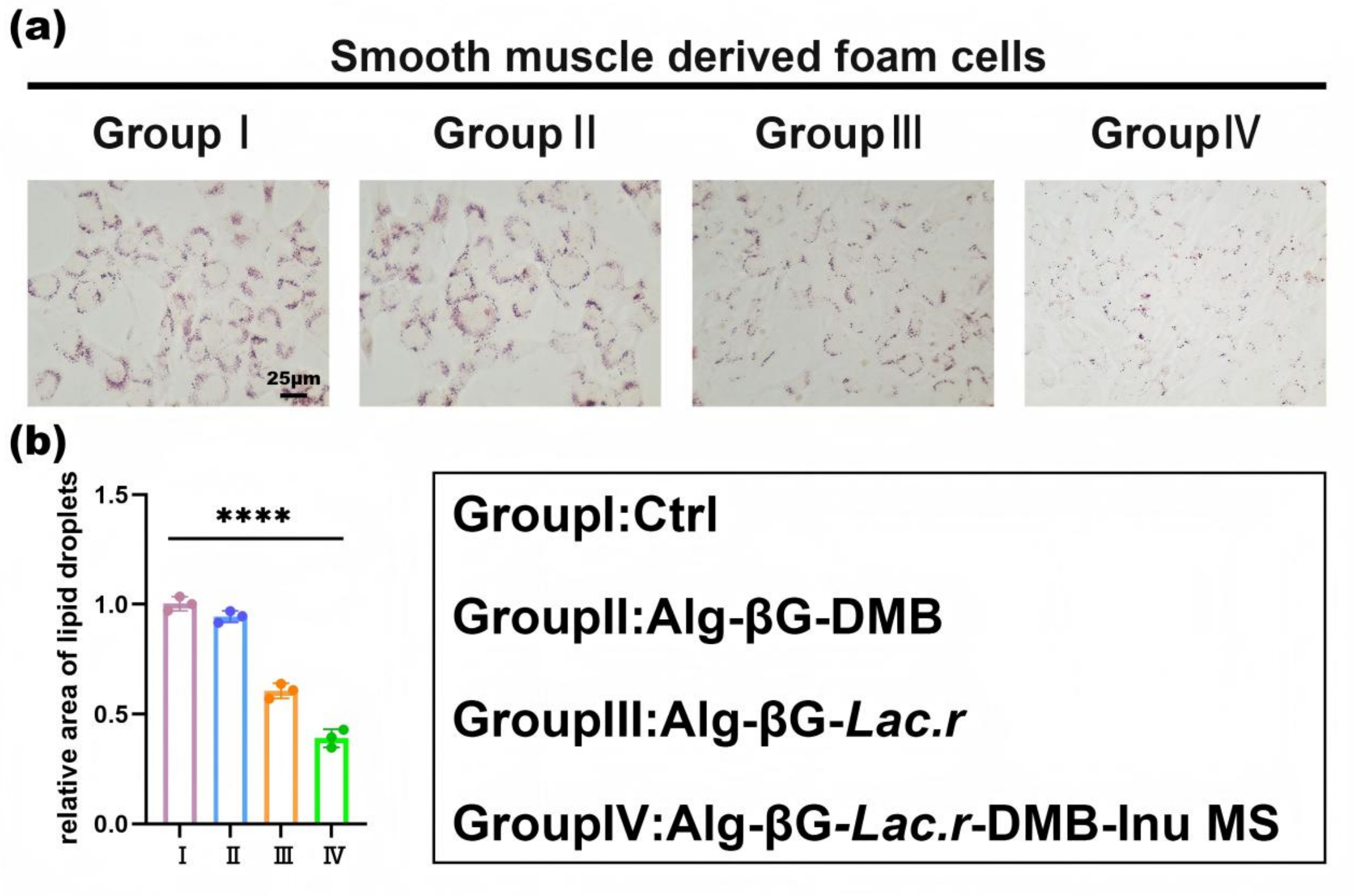

Fig. S23 ORO staining of smooth muscle-derived foam cells across four treatment groups. (a) Representative ORO-stained images of foam cells in Group I (Ctrl), Group II (Alg–βG–DMB), Group III (Alg–βG–*Lac.r*), and Group IV (Alg–βG–*Lac.r*–DMB–Inu). **(b)** Quantification of relative lipid droplet area corresponding to the four groups.*$P < 0.05$; **$P < 0.01$; ***$P < 0.001$; ****$P < 0.0001$.

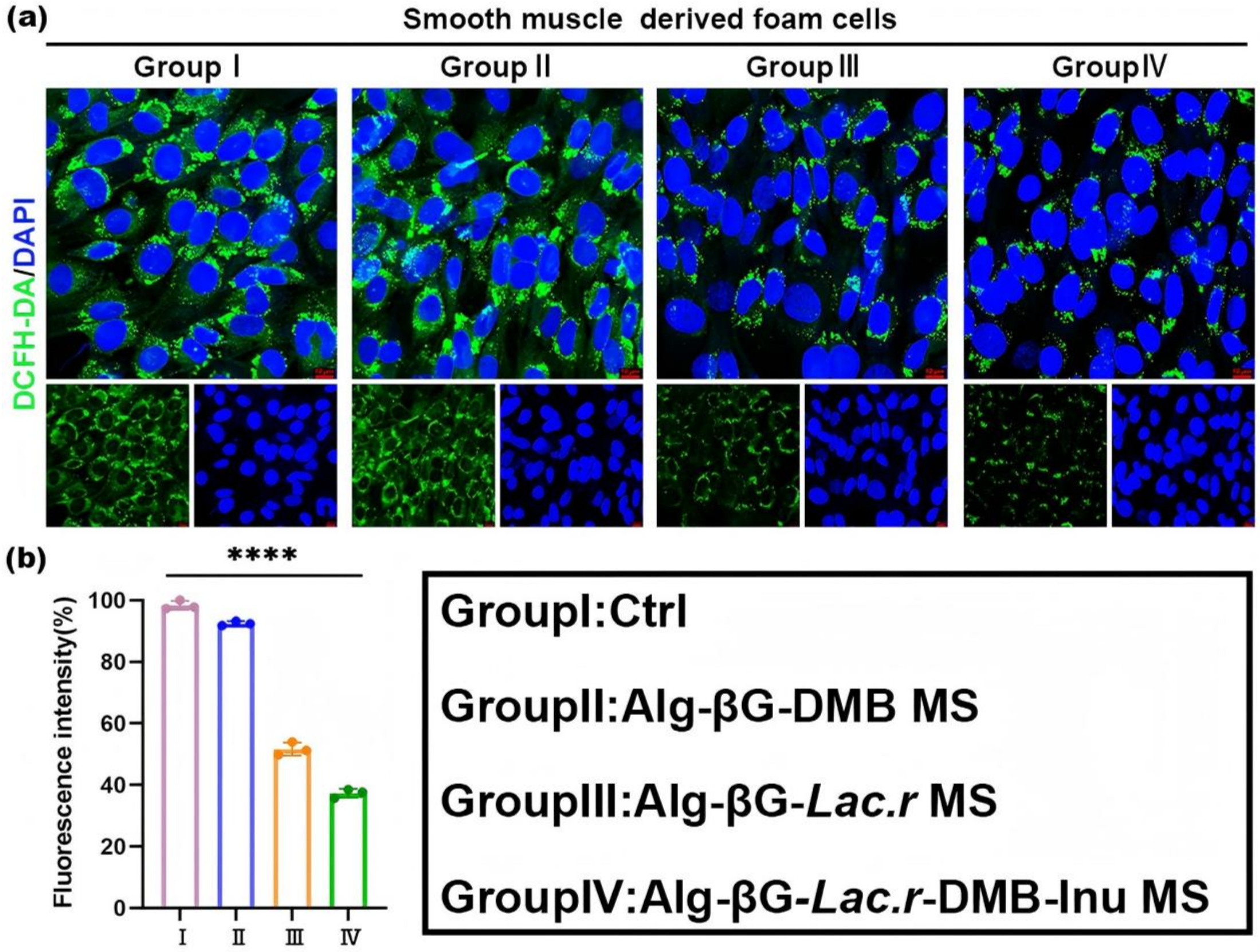

Fig. S24 DCFH-DA staining of smooth muscle-derived foam cells under different treatments. (a) Representative confocal images of foam cells in Group I (Ctrl), Group II (Alg–βG–DMB), Group III (Alg–βG–*Lac.r*), and Group IV (Alg–βG–*Lac.r*–DMB–Inu), stained with DCFH-DA (green) and DAPI (blue). **(b)** Quantification of fluorescence intensity corresponding to the four groups.*$P < 0.05$; **$P < 0.01$; ***$P < 0.001$; ****$P < 0.0001$.

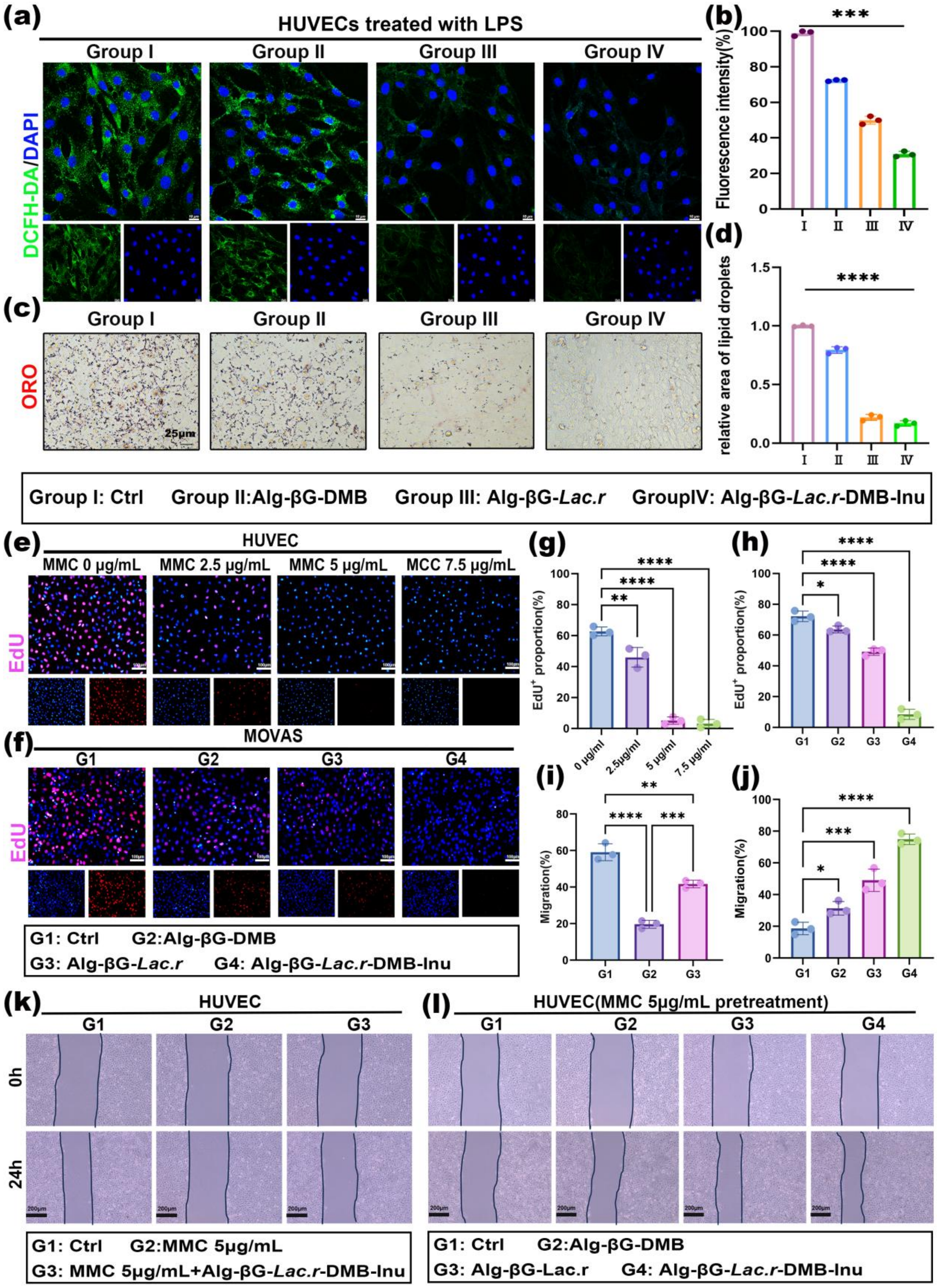

Fig. S25 Encapsulated synbiotic microspheres alleviate oxidative stress and lipid accumulation while preserving physiological endothelial migration and suppressing smooth muscle cell proliferation. (a) Representative confocal images of DCFH-DA (green) and DAPI (blue) staining in LPS-treated HUVECs following

different treatments: Group I, Ctrl; Group II, Alg–βG–DMB; Group III, Alg–βG–*Lac.r*; and Group IV, Alg–βG–*Lac.r*–DMB–Inu MS. Scale bars, 10 μm. **(b)** Quantification of DCFH-DA fluorescence intensity.Scale bars, 25 μm.**(c)** Representative Oil Red O (ORO)-stained images of HUVECs following the indicated treatments. **(d)** Quantification of relative lipid droplet area. **(e)** Representative EdU (red) and DAPI (blue) staining of HUVECs following treatment with the indicated concentrations of mitomycin C (MMC). Scale bars, 100 μm. **(f)** Representative EdU and DAPI images of MOVAS cells. Scale bars, 100 μm. G1, Ctrl; G2，Alg–βG–DMB; G3, Alg–βG–*Lac.r*; G4, Alg–βG–*Lac.r*–DMB–Inu. **(g)** Quantification of EdU-positive HUVECs at the indicated MMC concentrations (n = 3). **(h)** Quantification of EdU-positive MOVAS cells following the indicated treatments (n = 3). **(i)** Quantification of HUVEC migration in the wound-healing assay (n = 3). **(j)** Quantification of HUVEC (MMC 5 μg mL^{-1} pretreatment) migration in the wound-healing assay (n = 3). **(k)** Representative wound-healing images of untreated HUVECs. G1, Ctrl; G2, Alg–βG–*Lac.r*; G3, Alg–βG–*Lac.r*–DMB–Inu MS. Scale bars, 200 μm. **(l)** Representative wound-healing images of HUVECs pretreated with 5 μg mL^{-1} MMC. G1, Ctrl; G2, MMC (5 μg mL^{-1})+ Alg–βG–DMB; G3, MMC (5 μg mL^{-1}) + Alg–βG–*Lac.r*; G4, MMC (5 μg mL^{-1}) + Alg–βG–*Lac.r*–DMB–Inu. Scale bars, 200 μm. Data are presented as mean ± SD. Statistical significance was determined by one-way ANOVA followed by Tukey's multiple-comparisons test. *$P < 0.05$, **$P < 0.01$, ***$P < 0.001$, and ****$P < 0.0001$.

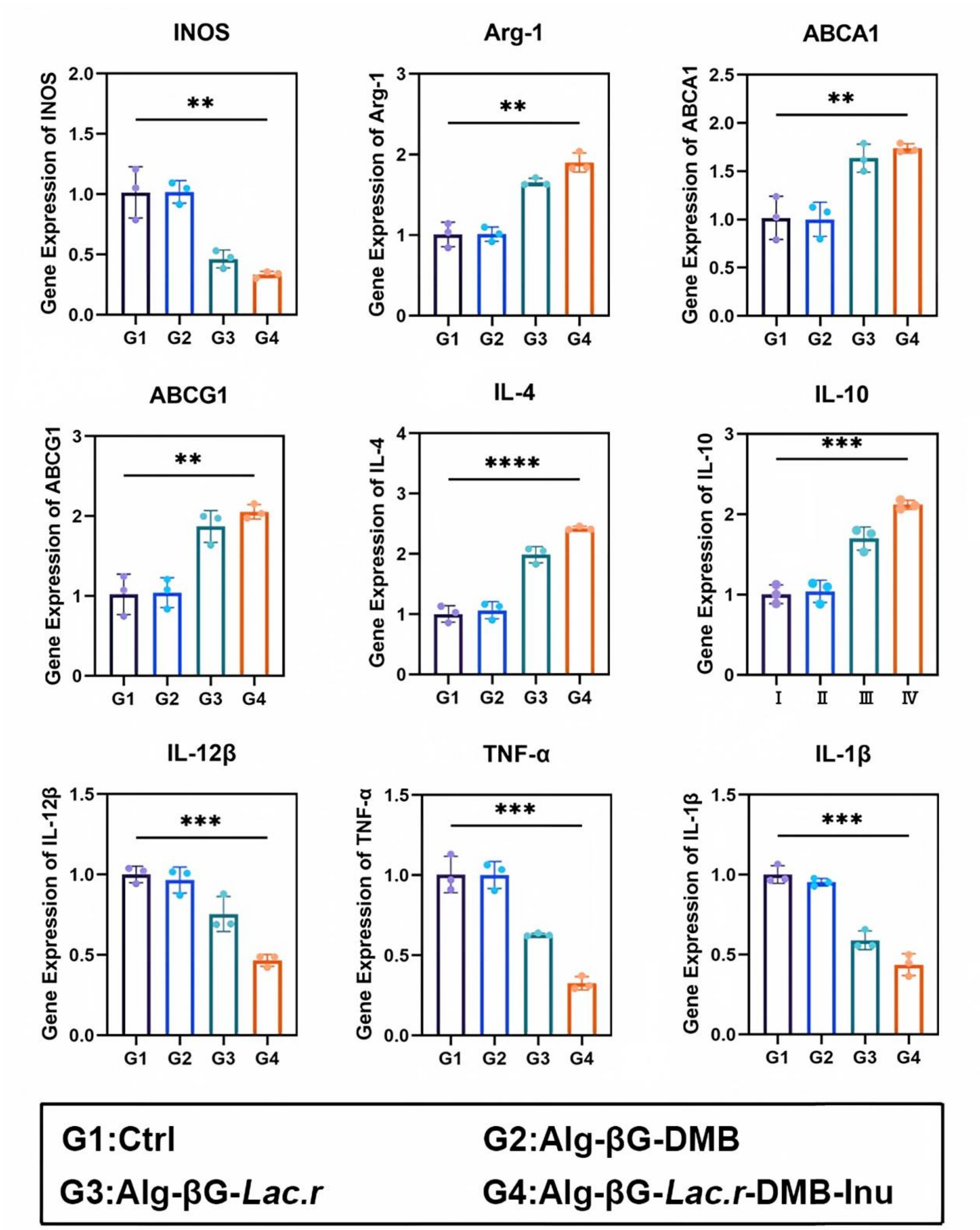

Fig. S26 qRT-PCR analysis of gene expression in macrophage-derived foam cells. RAW264.7 macrophages were induced to form foam cells and treated with different microsphere formulations, including Ctrl (G1), Alg–βG–DMB (G2), Alg–βG–*Lac.r* (G3), and Alg–βG–*Lac.r*–DMB–Inu (G4). Data are presented as mean ± SEM from three independent experiments. Statistical significance indicated in the figure.*$P < 0.05$; **$P < 0.01$; ***$P < 0.001$; ****$P < 0.0001$.

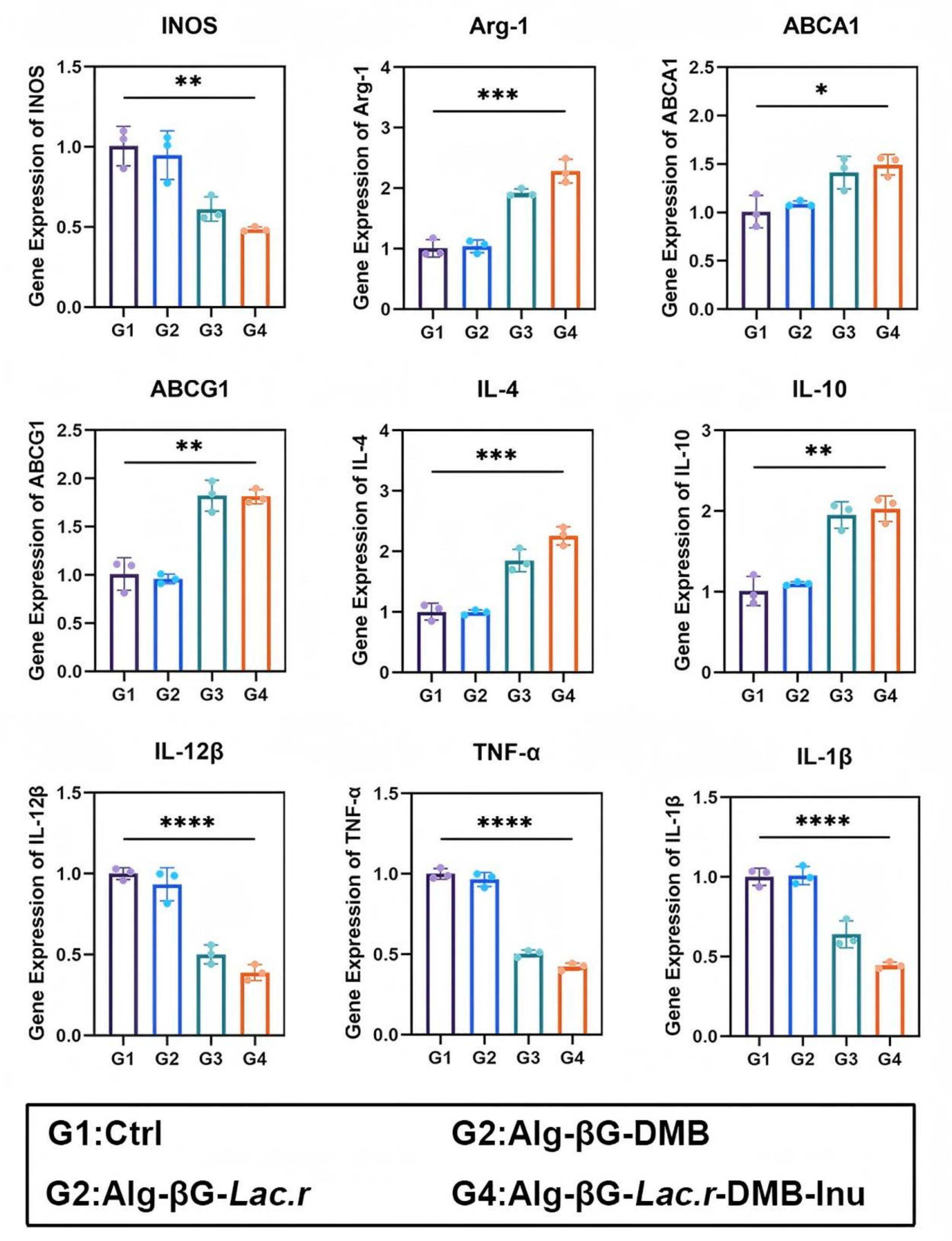

Fig. S27 qRT-PCR analysis of gene expression in smooth muscle cell-derived foam cells. MOVAS smooth muscle cells were induced to form foam cells and treated with different microsphere formulations, including Ctrl (G1), Alg–βG–DMB (G2), Alg–βG–*Lac.r* (G3), and Alg–βG–*Lac.r*–DMB–Inu (G4). Data are presented as mean

± SEM from three independent experiments. Statistical significance indicated in the figure.*$P < 0.05$; **$P < 0.01$; ***$P < 0.001$; ****$P < 0.0001$.

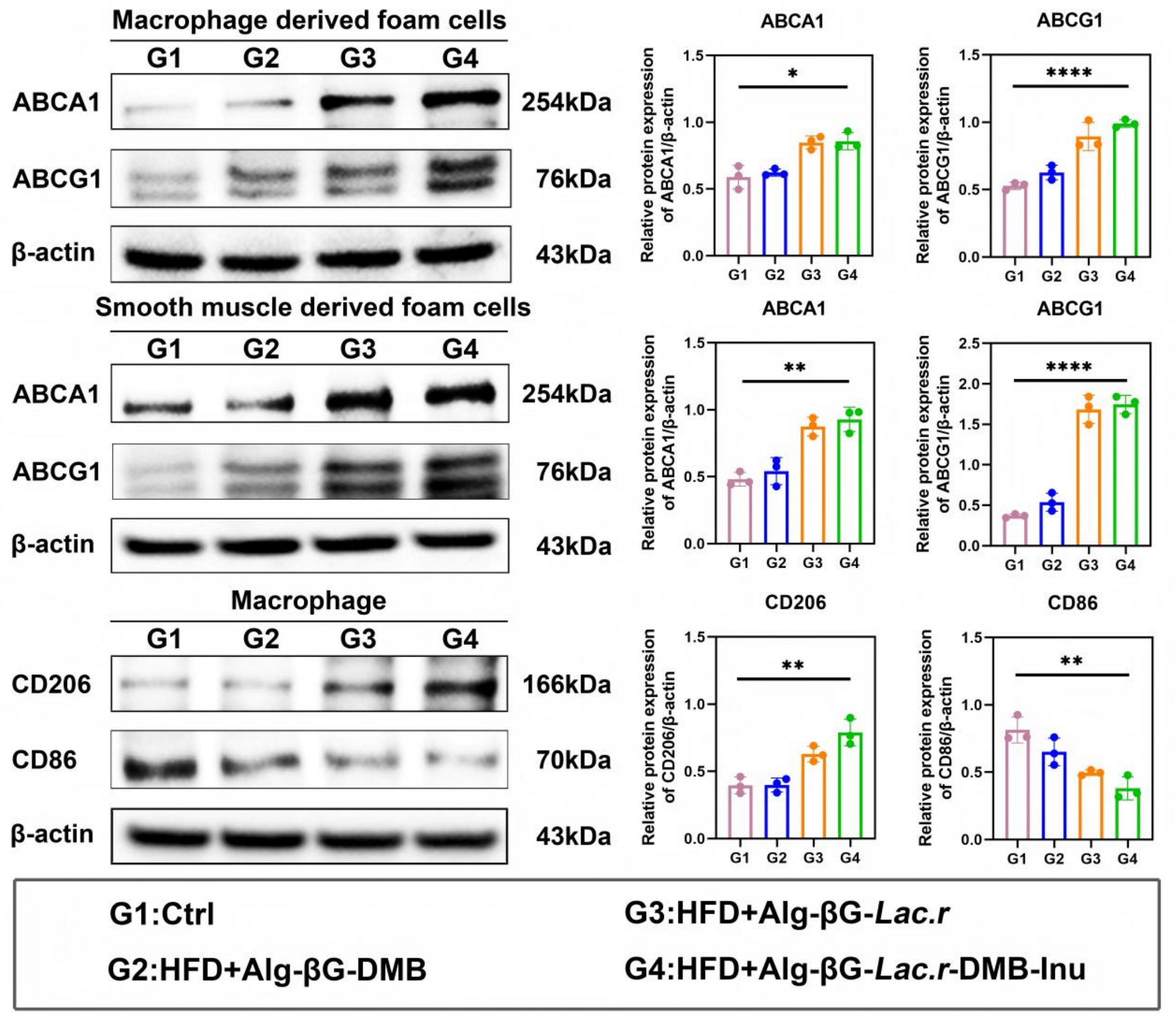

Fig. S28 Western blot analysis of protein expression in macrophage- and smooth muscle cell-derived foam cells. Groups: Ctrl (G1), Alg–βG–DMB (G2), Alg–βG–*Lac.r* (G3), and Alg–βG–*Lac.r*–DMB–Inu (G4).*$P < 0.05$; **$P < 0.01$; ***$P < 0.001$; ****$P < 0.0001$.

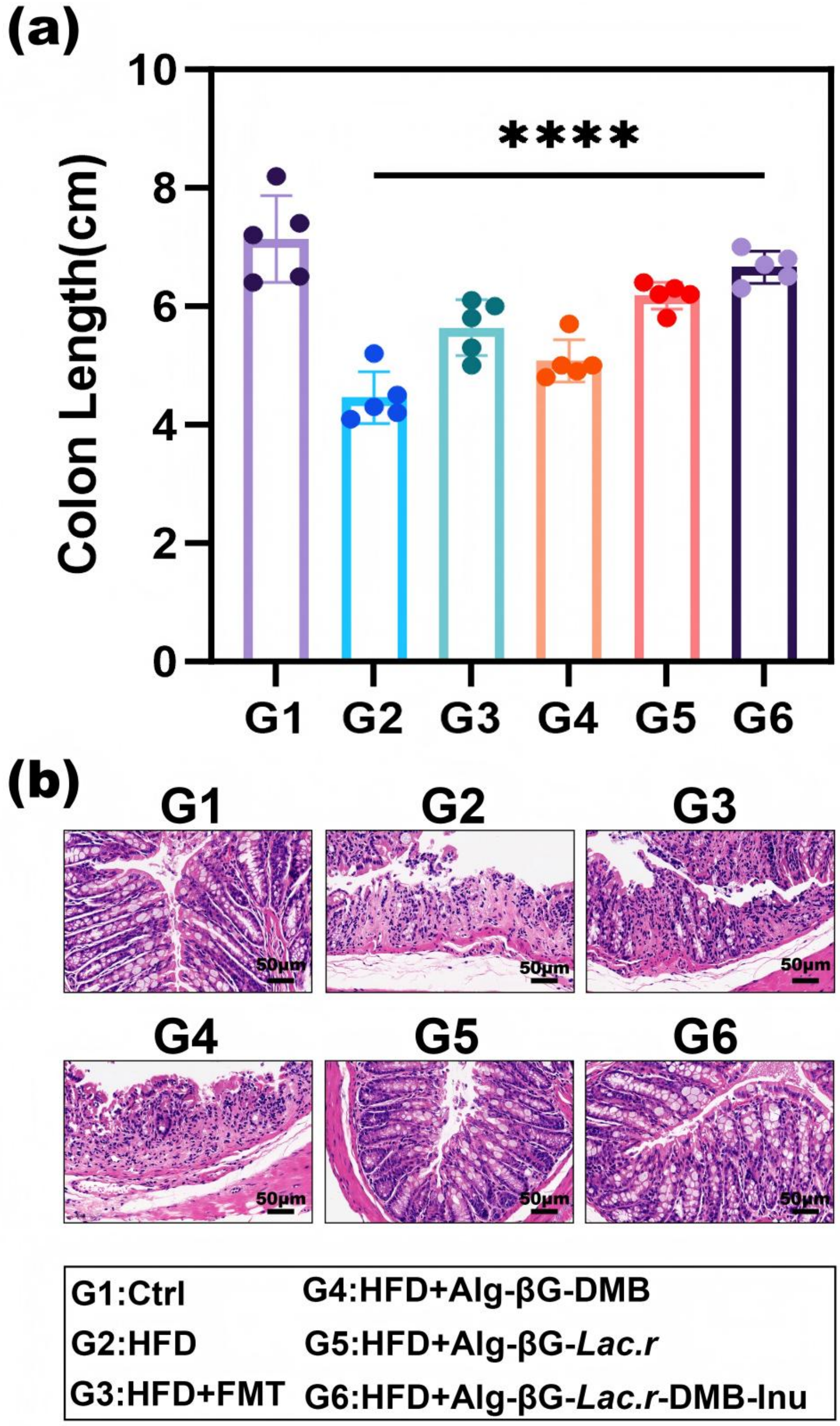

Fig. S29 Assessment of colonic morphology across treatment groups. (a) Quantification of colon length in six experimental groups, corresponding to the treatment regimens shown in **Fig. 6b**. **(b)** Representative hematoxylin and eosin-stained sections of colonic tissues from each group, illustrating histological features of the colonic mucosa and crypt architecture. Groups: G1, Ctrl; G2, HFD; G3, HFD + FMT; G4, HFD + Alg–βG–DMB; G5, HFD + Alg–βG–*Lac.r*; G6, HFD +

Alg–βG–*Lac.r*–DMB–Inu.

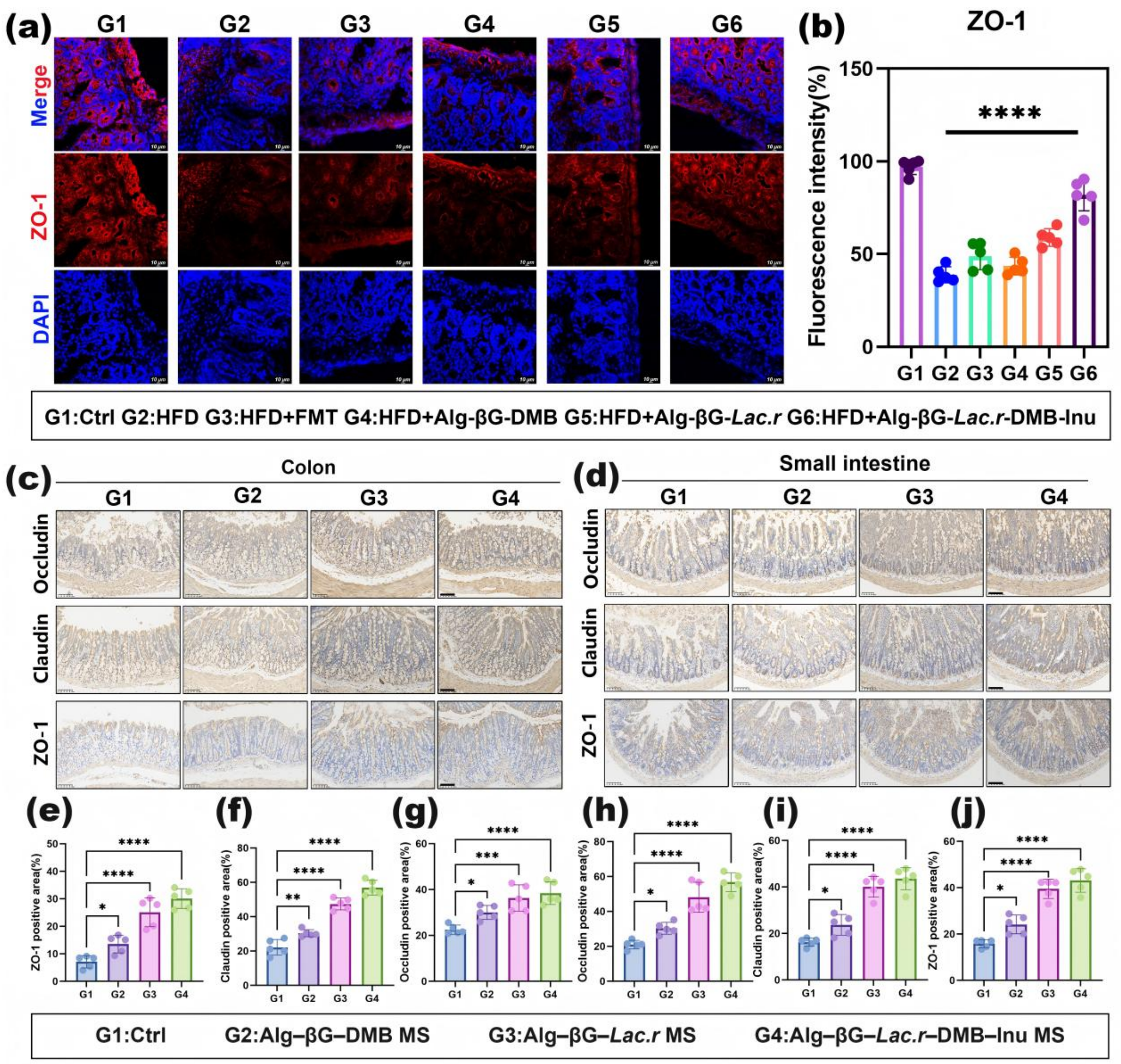

Fig. S30 Alg–βG–*Lac.r*–DMB–Inu microspheres restore intestinal tight junction integrity in the colon and small intestine. (a) Representative immunofluorescence images of ZO-1 (red) and nuclei (DAPI, blue) in colonic tissues from G1, Ctrl; G2, HFD; G3, HFD + FMT; G4, HFD + Alg–βG–DMB; G5, HFD + Alg–βG–*Lac.r*; and G6, HFD + Alg–βG–DMB–Inu. Scale bars, 10 μm. **(b)** Quantification of ZO-1 fluorescence intensity. **(c)** Representative immunohistochemical staining of Occludin, Claudin-1, and ZO-1 in colonic tissues. G1, Ctrl; G2, Alg–βG–DMB MS; G3, Alg–βG–*Lac.r* MS; G4, Alg–βG–*Lac.r*–DMB–Inu MS. Scale bars, 50 μm. **(d)** Representative immunohistochemical staining of Occludin, Claudin-1, and ZO-1 in small-intestinal tissues. Grouping is identical to that in (c). Scale bars, 50 μm. **(e–g)**

Quantification of ZO-1, Claudin-1, and Occludin positive areas in the colon (n = 5). **(h–j)** Quantification of Occludin, Claudin-1, and ZO-1 positive areas in the small intestine (n = 5). Data are presented as mean ± SD. Statistical significance was determined by one-way ANOVA followed by Tukey's multiple-comparisons test. *$P < 0.05$, **$P < 0.01$, ***$P < 0.001$, and ****$P < 0.0001$.

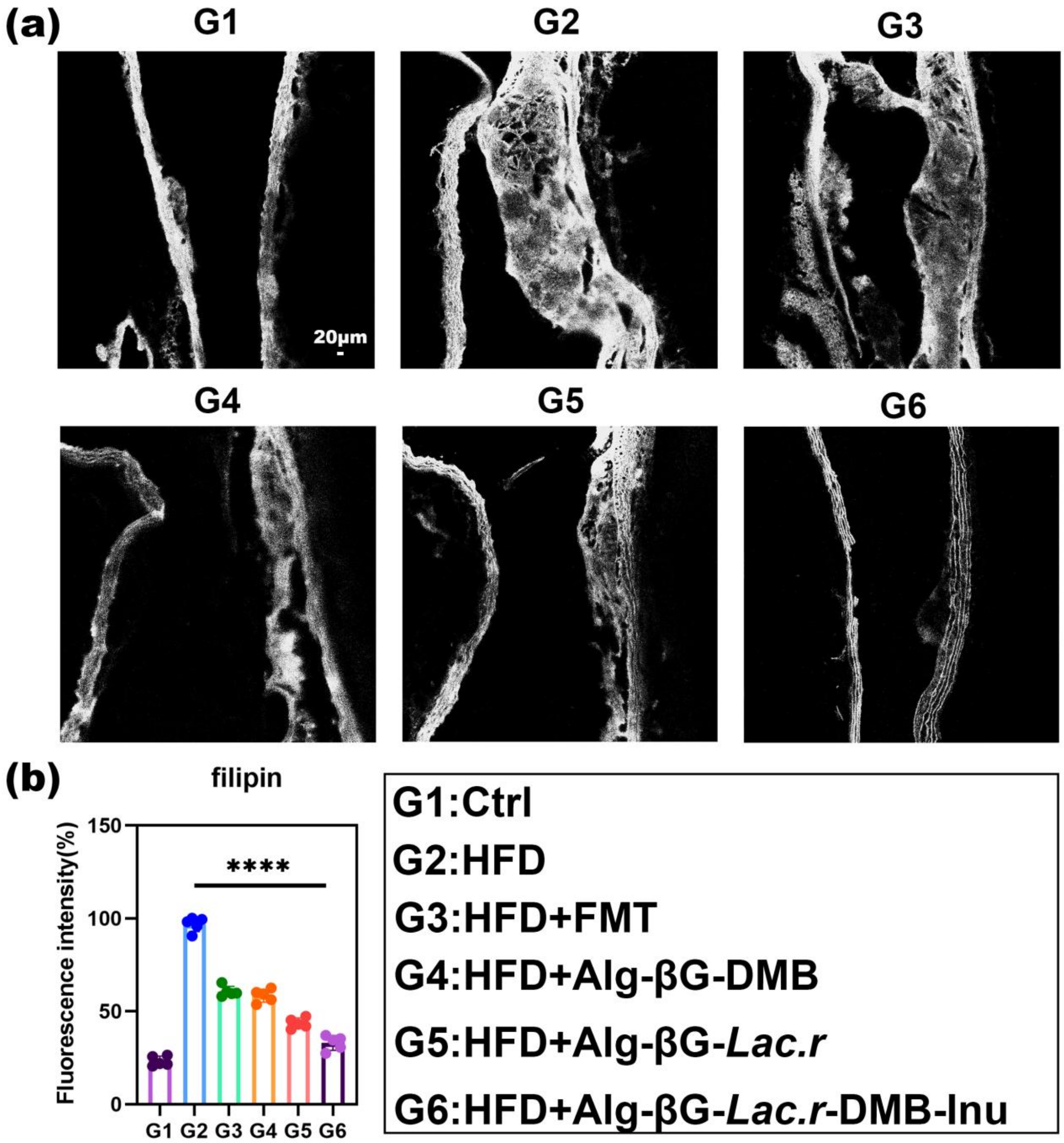

Fig. S31 Filipin staining of aortic plaques across six treatment groups. (a) Representative filipin-stained images from Group 1 (Ctrl), Group 2 (HFD), Group 3 (HFD + FMT), Group 4 (HFD + Alg–βG–DMB), Group 5 (HFD + Alg–βG–*Lac.r*), and Group 6 (HFD + Alg–βG–*Lac.r*–DMB–Inu). **(b)** Quantification of filipin fluorescence intensity corresponding to the six groups.*$P < 0.05$; **$P < 0.01$; ***$P < 0.001$; ****$P < 0.0001$.

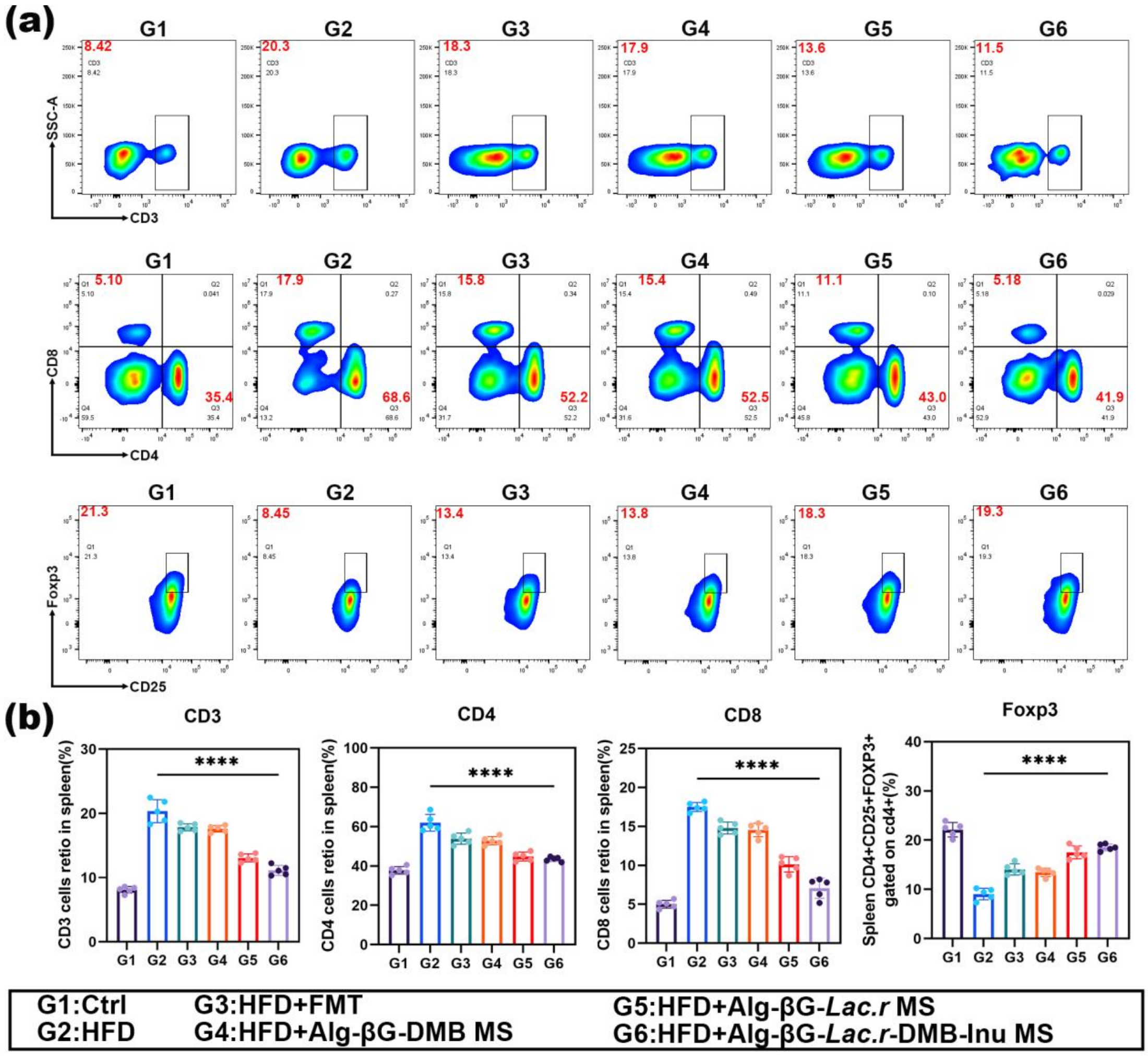

Fig. S32 Flow cytometric analysis of splenic T-cell subsets in different treatment groups. (a) Representative flow cytometry plots showing the gating strategy and distribution of $CD3^+$ T cells, $CD4^+$ and $CD8^+$ T-cell subsets, and $CD4^+CD25^+Foxp3^+$ regulatory T cells (Tregs) in spleens from mice subjected to different treatments. Sequential gating was performed to identify $CD3^+$ lymphocytes, followed by delineation of $CD4^+$ and $CD8^+$ populations and subsequent identification of $Foxp3^+$ Tregs within the $CD4^+$ compartment. (b) Quantitative analysis of the proportions of splenic $CD3^+$, $CD4^+$, $CD8^+$ T cells, and $CD4^+$ $CD25^+$ $Foxp3^+$ Tregs across groups. Data are presented as mean ± SEM. Statistical significance was assessed by one-way ANOVA with appropriate multiple-comparison tests. G1: Ctrl; G2: HFD; G3, HFD + FMT; G4, HFD + Alg–βG–DMB; G5, HFD + Alg–βG–*Lac.r*; G6, HFD + Alg–βG–*Lac.r*–DMB–Inu.**P* < 0.05; ***P* < 0.01; ****P* < 0.001; *****P* < 0.0001.

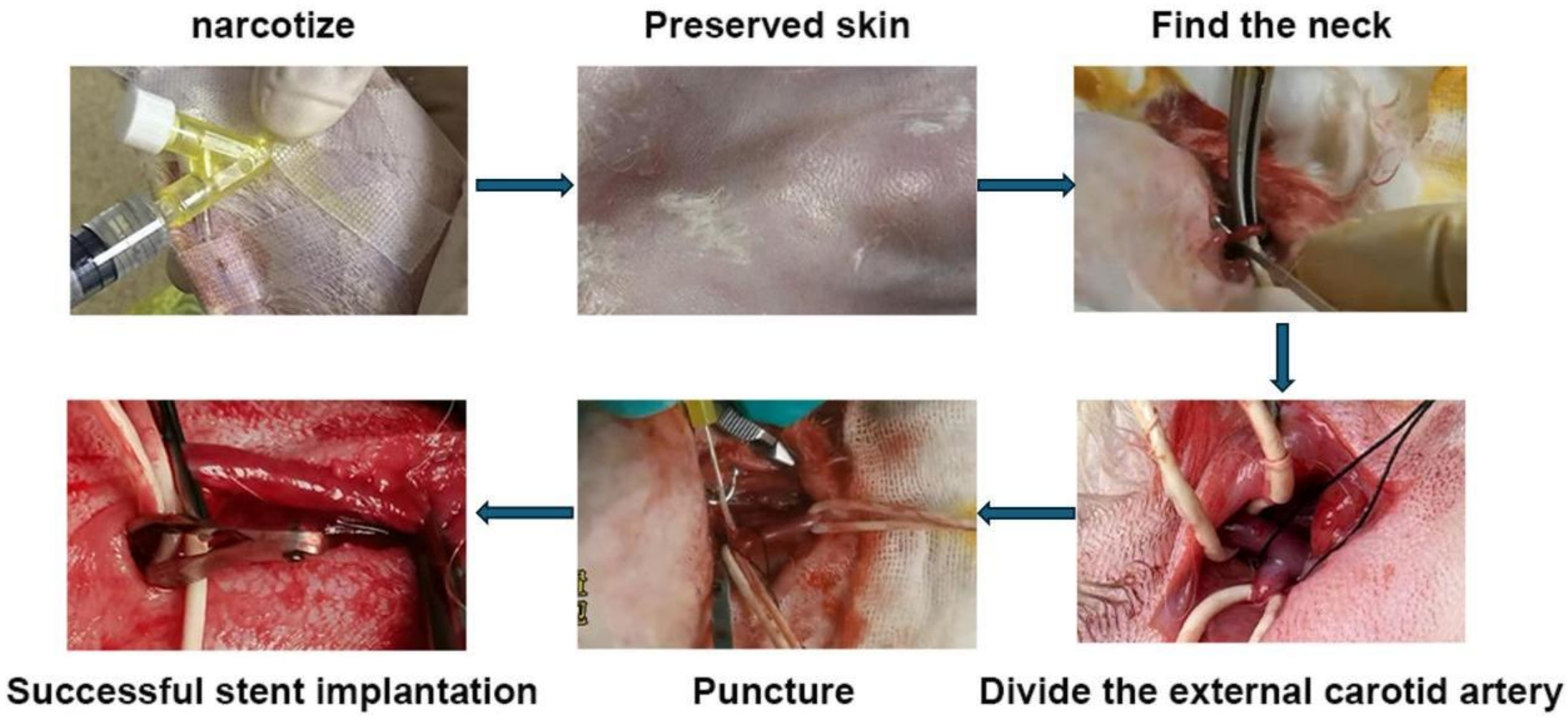

Fig. S33 Schematic workflow of the *in vivo* carotid stent-ISR model. Stepwise procedural illustration showing (i) induction of anesthesia, (ii) preparation of the surgical field with preserved cervical skin, (iii) identification and exposure of the neck region, (iv) isolation and ligation of the external carotid artery, (v) vascular puncture for endoluminal access, and (vi) successful deployment of the stent within the carotid artery. Images depict the key operative stages required to establish the *in situ* ISR model in rodents.

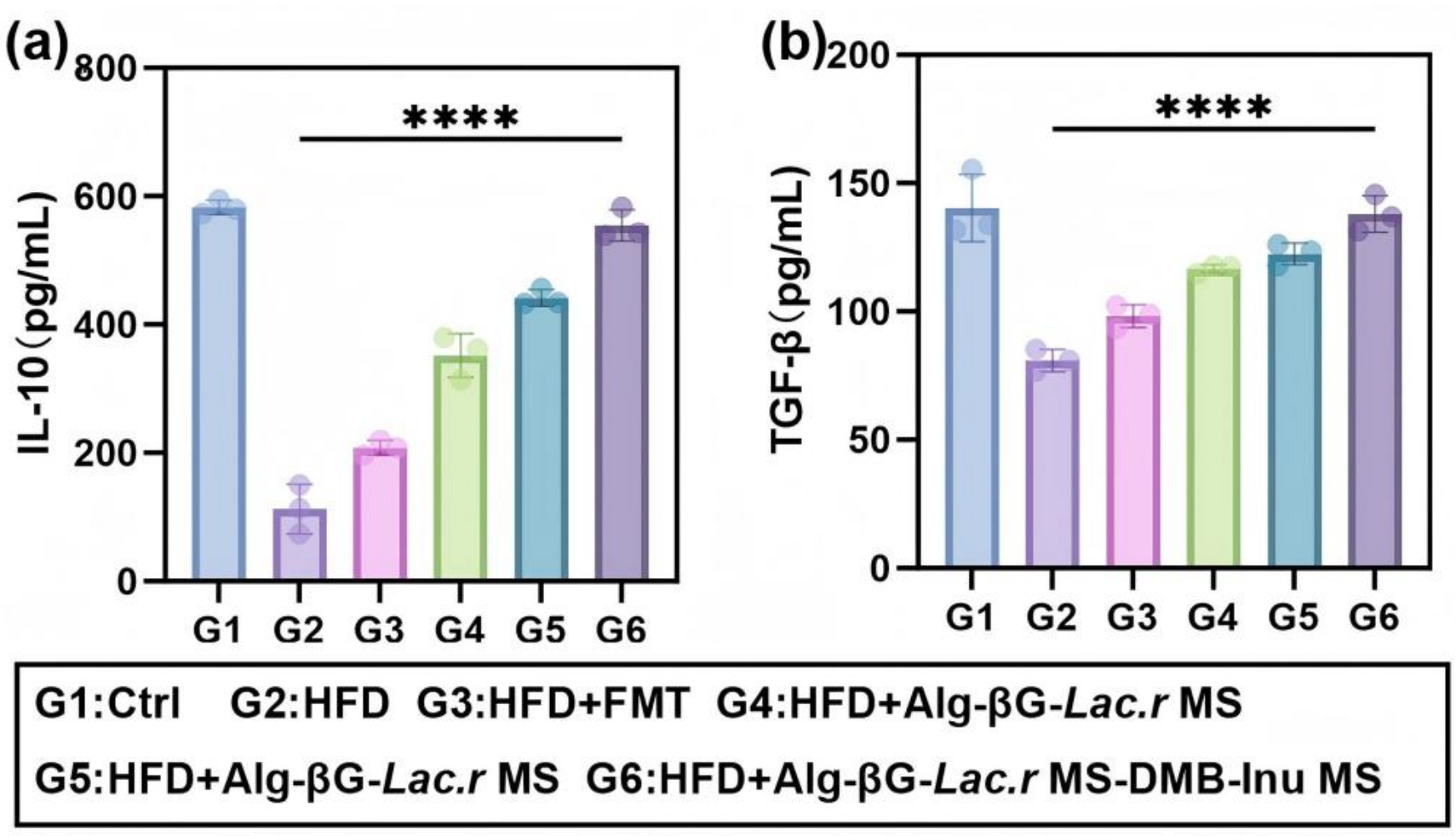

Fig. S34 Alg–βG–Lac.r–DMB–Inu microspheres enhance systemic anti-inflammatory responses in HFD-fed mice. (a) Serum IL-10 concentrations. **(b)** Serum transforming growth factor-β (TGF-β) concentrations. G1, Ctrl; G2, HFD; G3, HFD + FMT; G4, HFD + Alg–βG–DMB MS; G5, HFD + Alg–βG–*Lac.r* MS; and G6, HFD + Alg–βG–*Lac.r*–DMB–Inu MS. Data are presented as mean ± SD (n = 5 biologically independent animals). Statistical significance was determined by one-way ANOVA followed by Tukey's multiple-comparisons test. **P* < 0.05, ****P* < 0.0001.

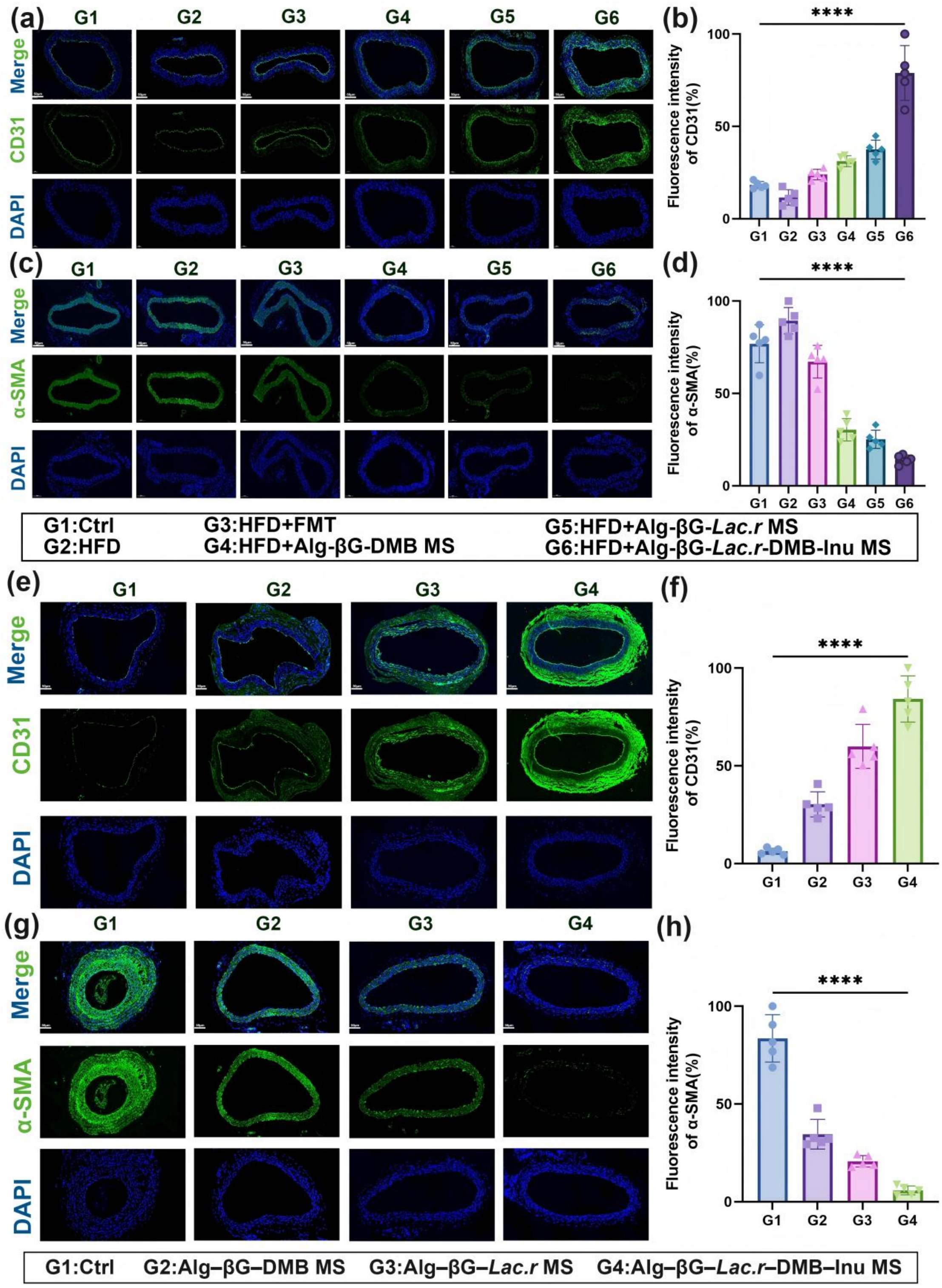

Fig. S35 Alg–βG–*Lac.r*–DMB–Inu microspheres promote vascular re-endothelialization and suppress smooth muscle remodeling *in vivo*. (a) Representative immunofluorescence images of CD31 (green) and nuclei (DAPI, blue) in aortic sections from G1, Ctrl; G2, HFD; G3, HFD + FMT; G4, HFD +

Alg–βG–DMB MS; G5, HFD + Alg–βG–*Lac.r* MS; and G6, HFD + Alg–βG–*Lac.r*–DMB–Inu MS. Scale bars, 100 μm. **(b)** Quantification of aortic CD31 fluorescence intensity (n = 5 biologically independent animals). **(c)** Representative immunofluorescence images of α-smooth muscle actin (α-SMA) (green) and DAPI (blue) in aortic sections from the same groups as in (a). Scale bars, 100 μm. **(d)** Quantification of aortic α-SMA fluorescence intensity (n = 5 biologically independent animals). **(e)** Representative immunofluorescence images of CD31 (green) and DAPI (blue) in arterial sections from ISR mice treated with different microsphere formulations. G1, Ctrl; G2, Alg–βG–DMB MS; G3, Alg–βG–*Lac.r* MS; G4, Alg–βG–*Lac.r*–DMB–Inu MS. Scale bars, 100 μm. **(f)** Quantification of CD31 fluorescence intensity in arterial sections (n = 5 biologically independent animals). **(g)** Representative immunofluorescence images of α-SMA (green) and DAPI (blue) in arterial sections from the same groups as in (e). Scale bars, 100 μm. **(h)** Quantification of α-SMA fluorescence intensity in arterial sections (n = 5 biologically independent animals). Data are presented as mean ± SD. Statistical significance was determined by one-way ANOVA followed by Tukey's multiple-comparisons test. *$P < 0.05$; **$P < 0.01$; ***$P < 0.001$; ****$P < 0.0001$.

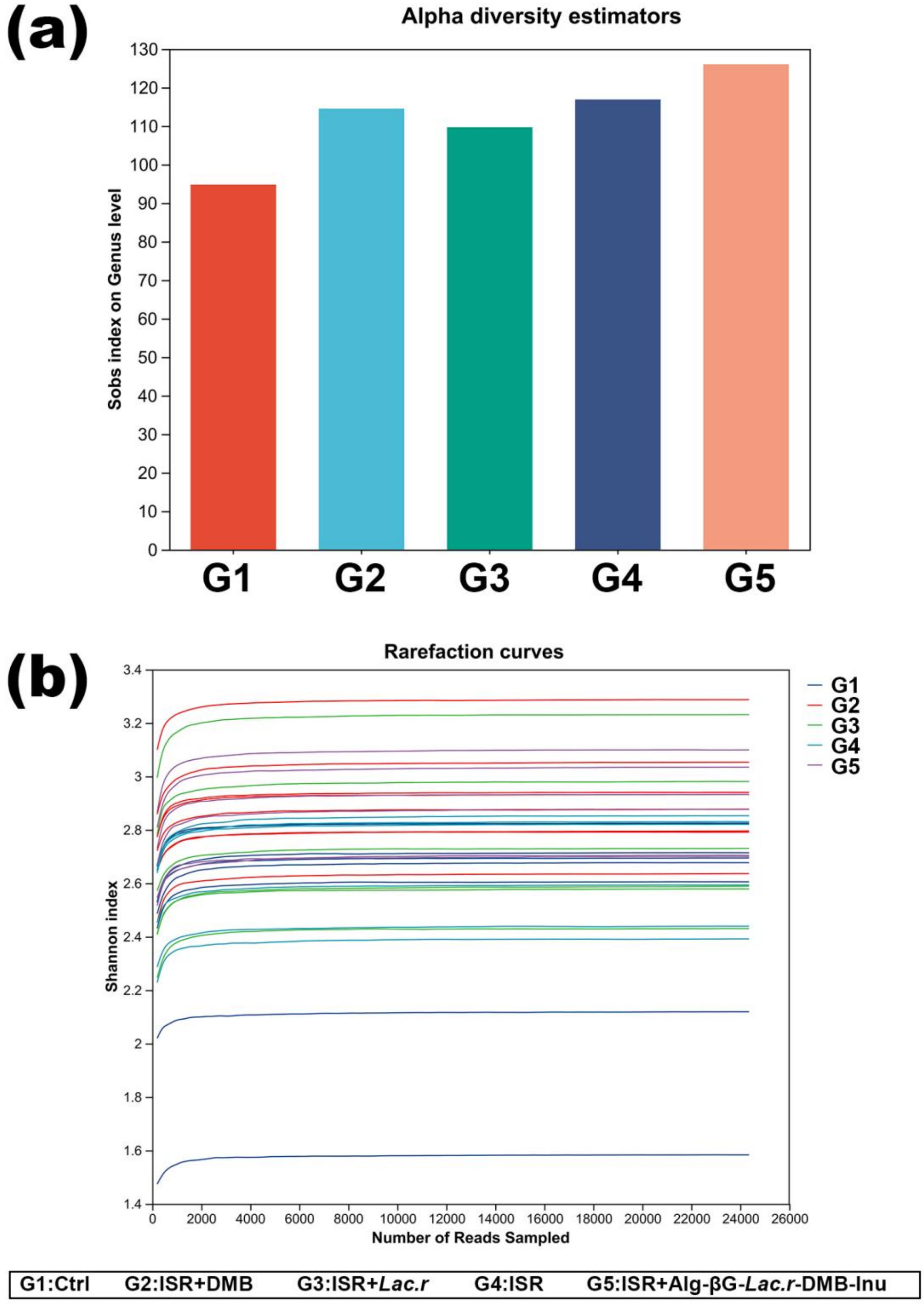

Fig. S36 α-diversity and sequencing depth assessment of the gut microbiota in in-stent restenosis (ISR) animal models. (a) Genus-level α-diversity analysis of gut microbial communities using the Sobs index across different treatment groups. **(b)** Shannon index-based rarefaction curves generated as a function of sequencing depth for each sample. Groups: G1, Ctrl; G2, ISR + DMB; G3, ISR +B*Lac.r*; G4, ISR; G5, ISR + Alg–βG–*Lac.r*–DMB–Inu.

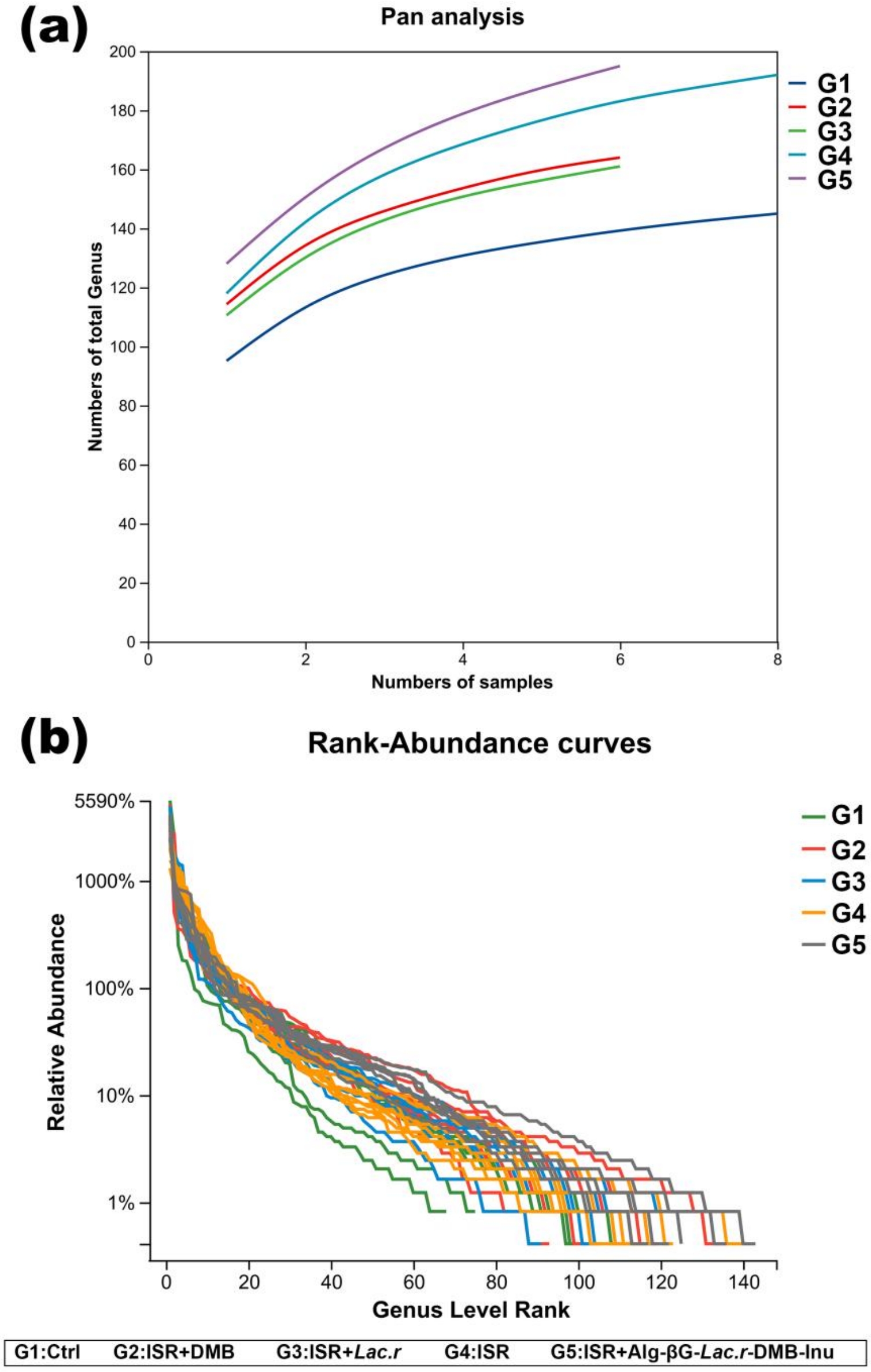

Fig. S37 Pangenome analysis and rank abundance profiling of gut microbiota in in-stent restenosis (ISR) animal models. (a) Pangenome analysis depicting the cumulative number of detected genera as a function of sample size at the genus level for each treatment group. **(b)** Genus-level rank abundance curves showing the relative abundance of taxa ordered by rank across samples. Groups: G1, Ctrl; G2, ISR + DMB; G3, ISR + *Lac.r*; G4, ISR; G5, ISR + Alg–βG–*Lac.r*–DMB–Inu.

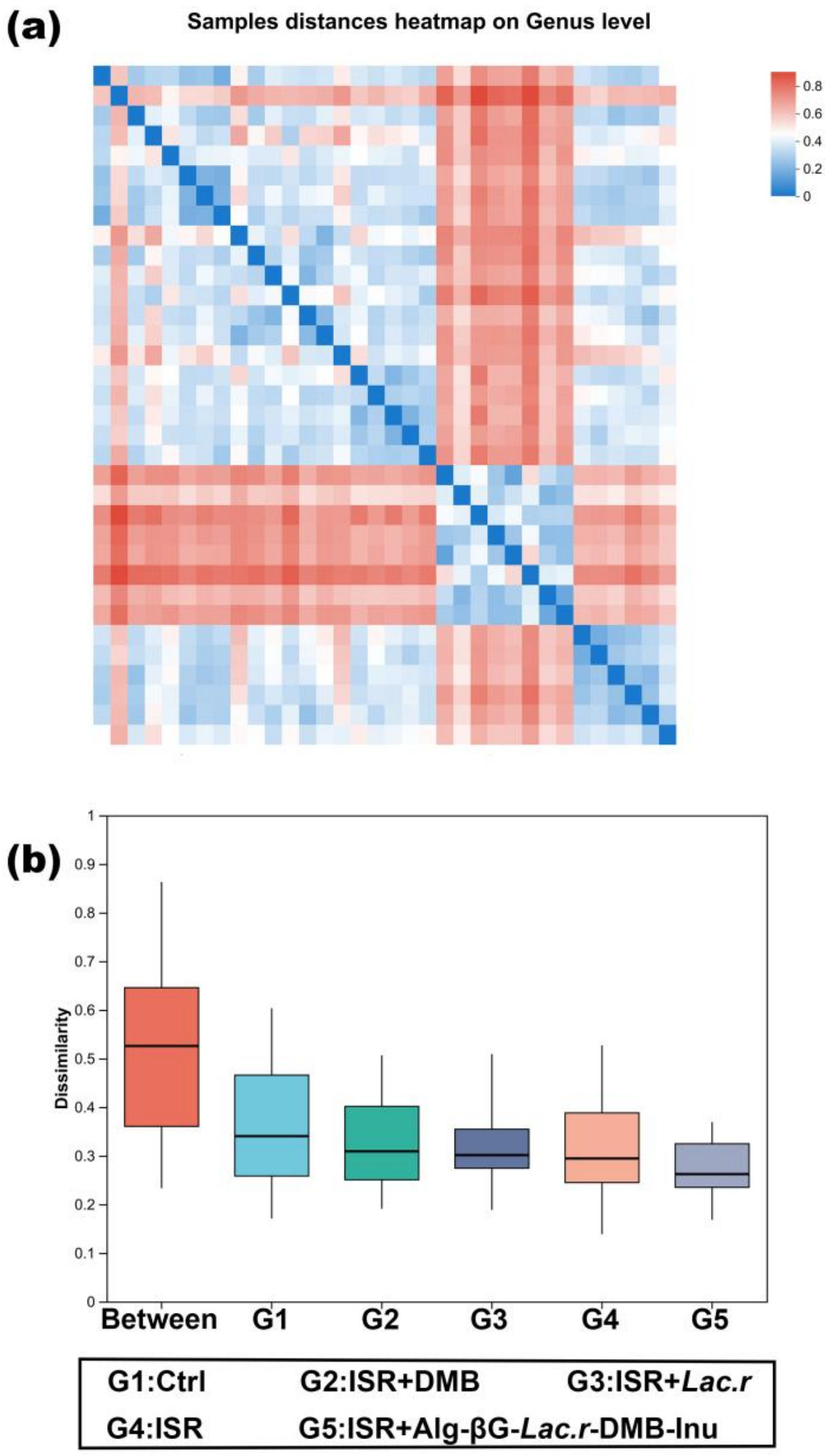

Fig. S38 Intra- and inter-group dissimilarity analysis of gut microbial communities at the genus level in in-stent restenosis (ISR) animal models. (a) Heatmap of pairwise sample dissimilarities calculated at the genus level across all samples. **(b)** Box plots summarizing genus-level dissimilarity values within individual groups and between groups. Groups: G1, Ctrl; G2, ISR + DMB; G3, ISR + *Lac.r*; G4, ISR; G5, ISR + Alg–βG–*Lac.r*–DMB–Inu. "Between" represents pairwise dissimilarities calculated between samples from different groups.

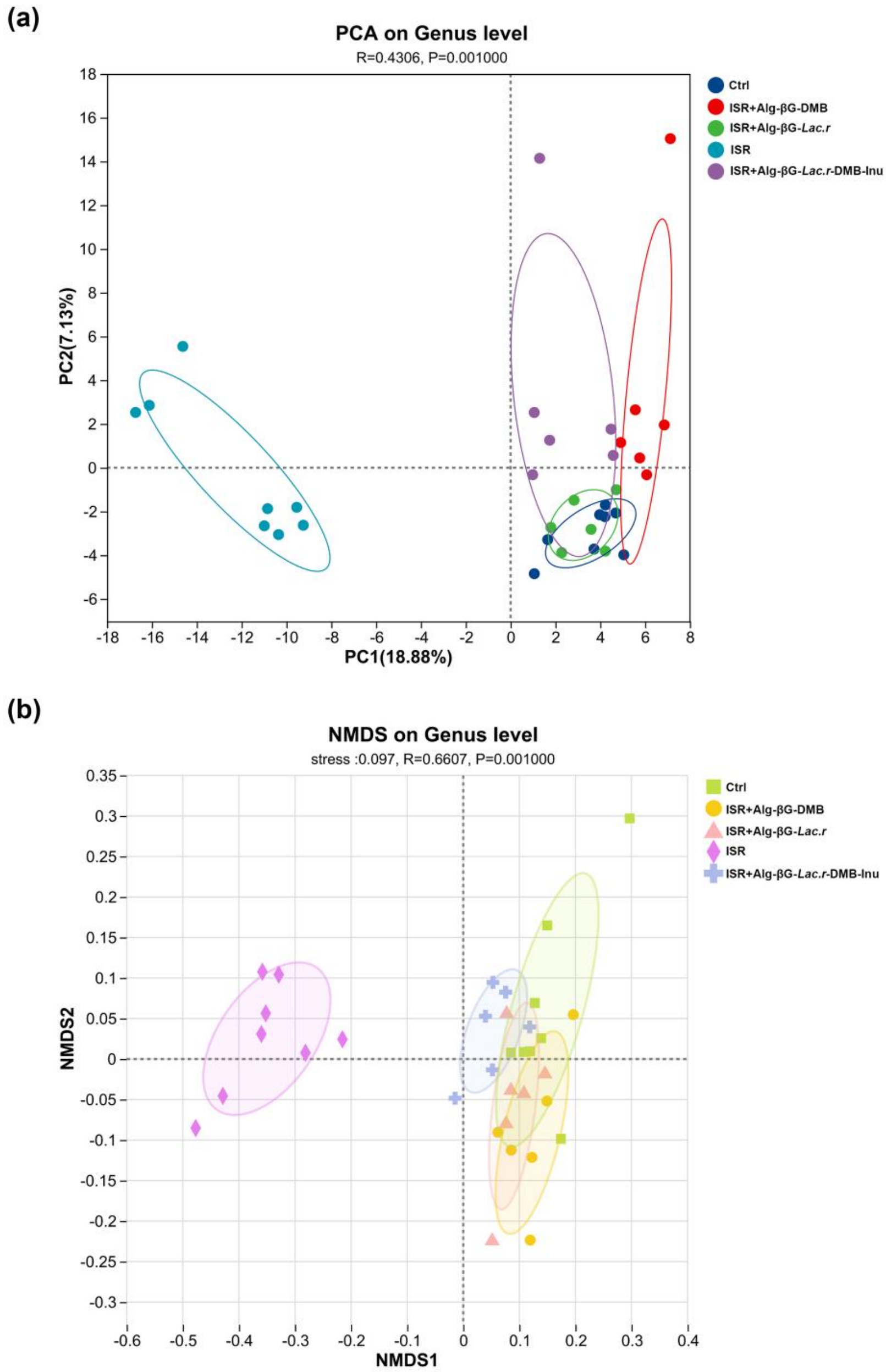

Fig. S39 Multivariate ordination analyses of gut microbial community structure at the genus level in ISR animal models. (a) Principal component analysis performed on genus-level microbial abundance profiles. Axes indicate the percentage of variance explained by each principal component. **(b)** Non-metric multidimensional scaling analysis based on genus-level dissimilarity matrices. Ellipses represent group-wise confidence regions. Stress values and permutation-based statistics are indicated where applicable.

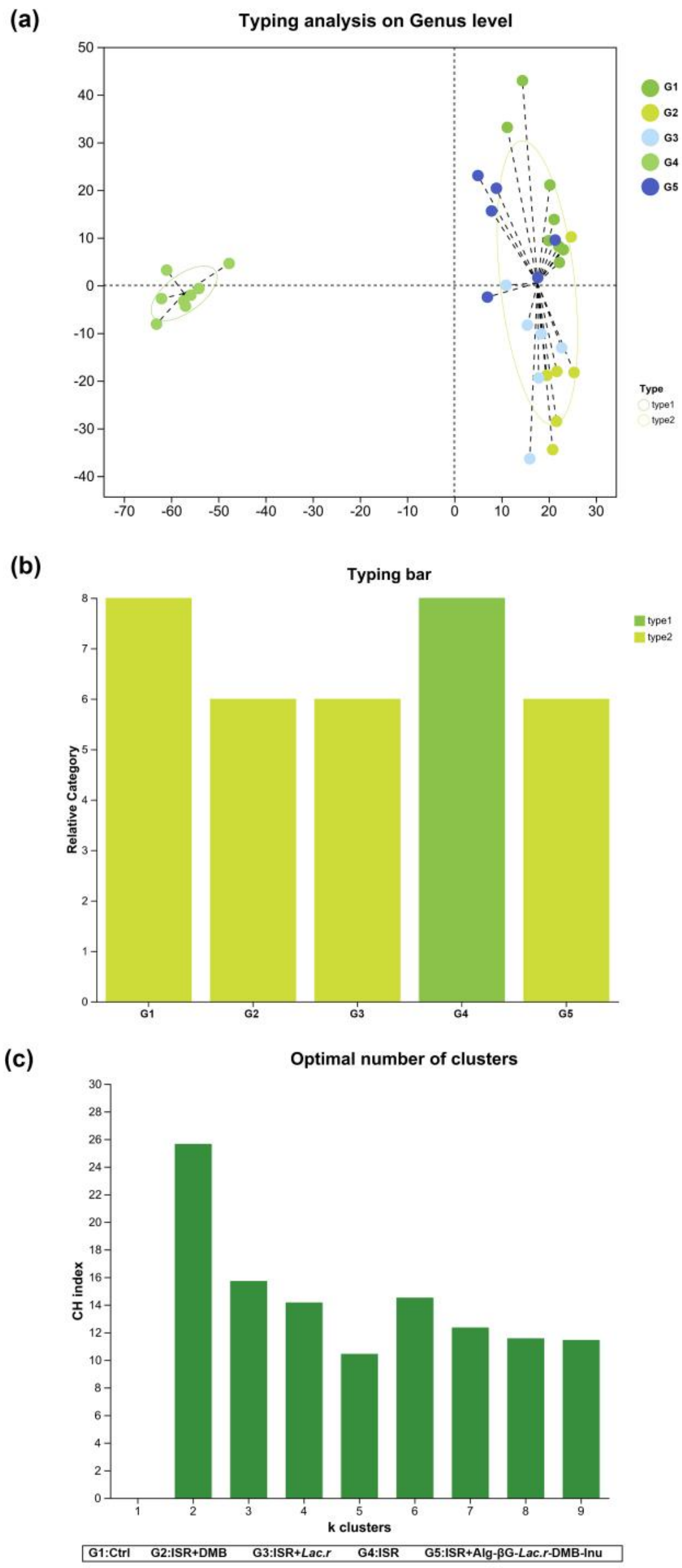

Fig. S40 Genus-level community typing and clustering analysis of gut microbiota in in-stent restenosis animal models. (a) Community typing analysis performed on genus-level gut microbial profiles, displaying sample distribution across inferred community types. Dashed lines indicate sample assignment relationships between community types; ellipses denote group-wise confidence regions. **(b)** Bar plot showing the distribution of community types across different treatment groups. **(c)** Evaluation of cluster numbers based on the Calinski–Harabasz index across a range of

k values.

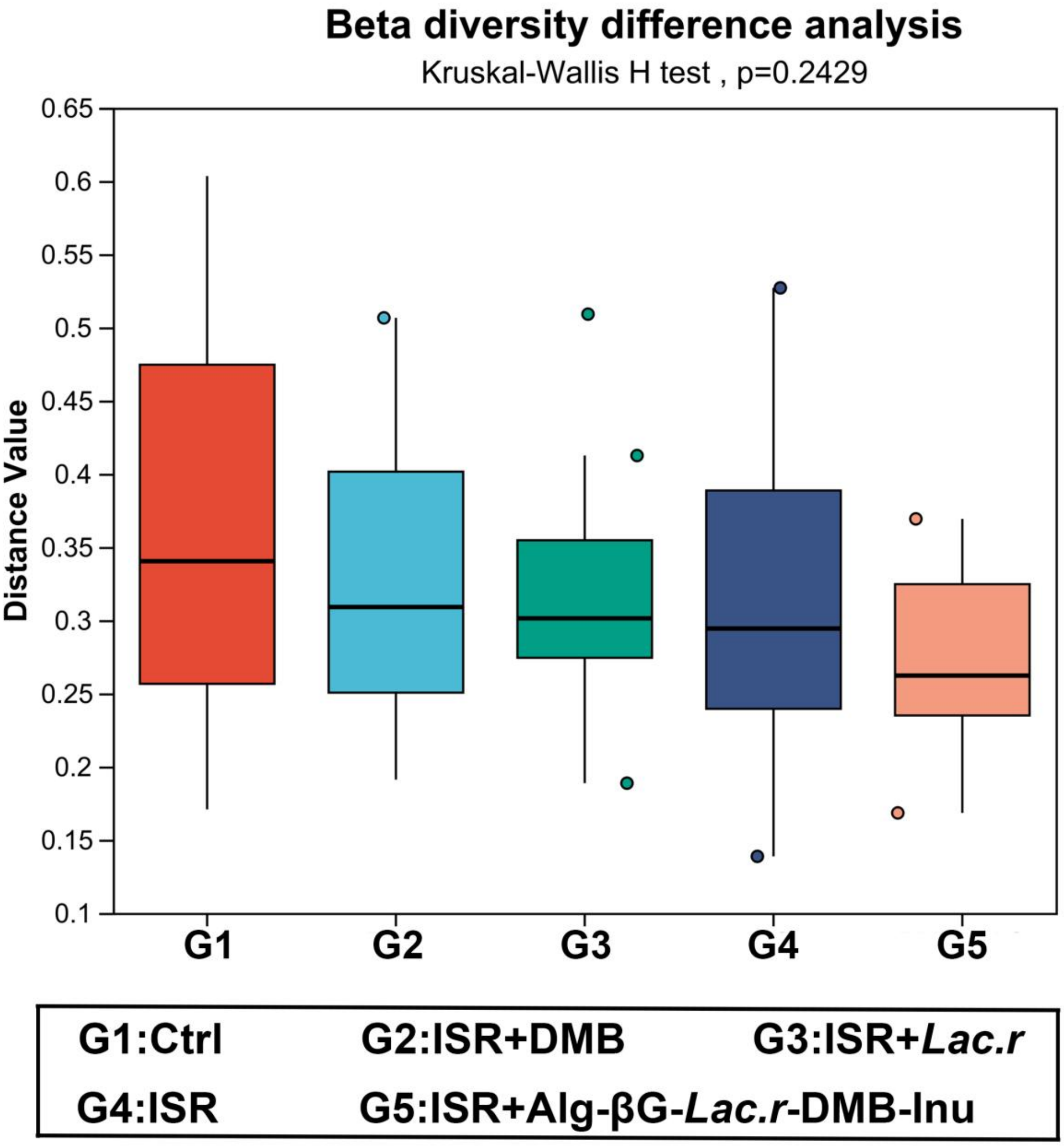

Fig. S41 Comparison of genus-level β-diversity distances among treatment groups in in-stent restenosis animal models. Box plots presenting the distribution of genus-level β-diversity distance values across different groups. Statistical comparison was performed using the Kruskal–Wallis H test.

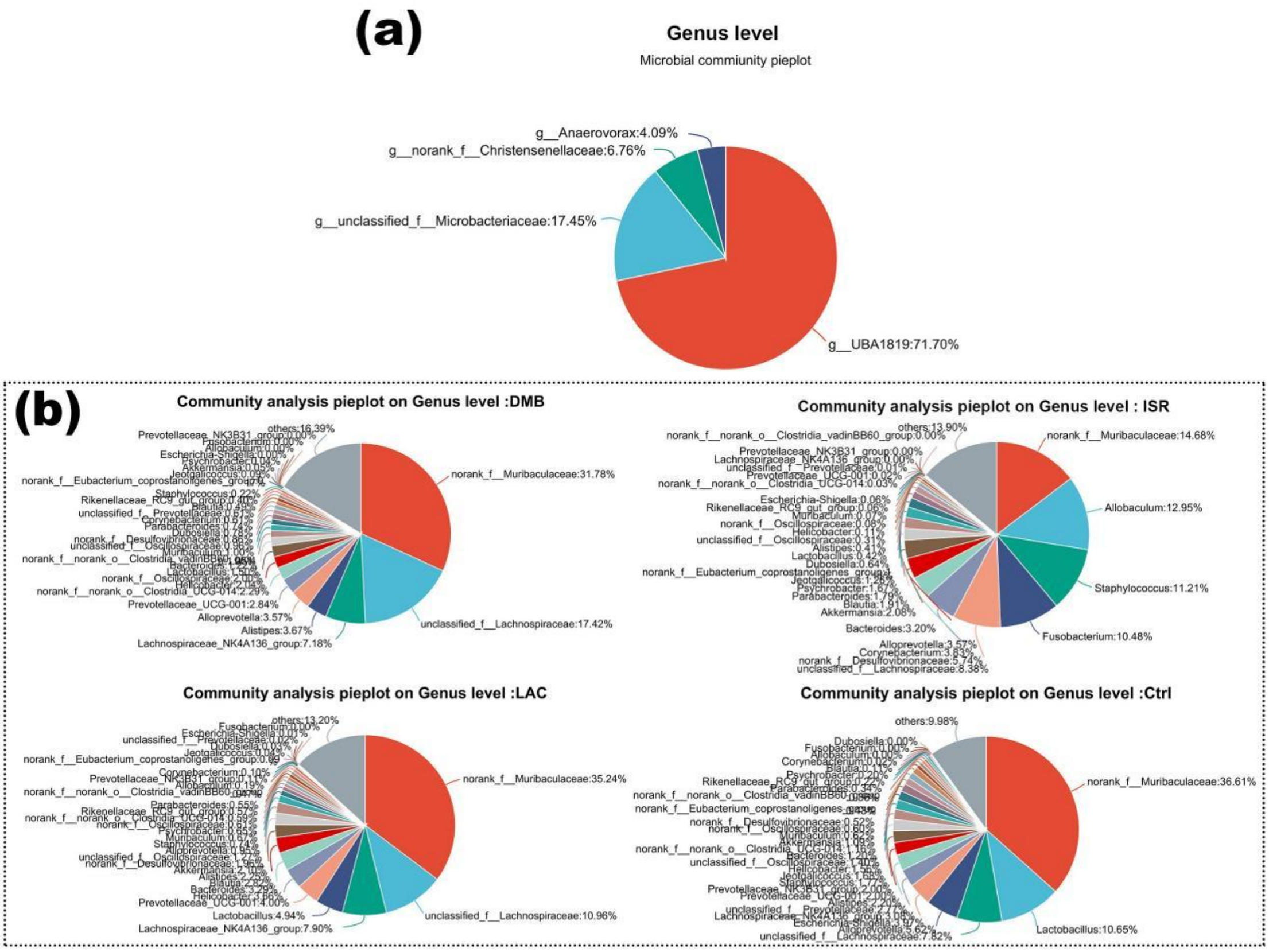

Fig. S42 Genus-level taxonomic composition of gut microbial communities in in-stent restenosis animal models. (a) Overall genus-level taxonomic composition of gut microbial communities across all samples. **(b)** Genus-level taxonomic composition for each treatment group. Genera are shown individually according to their relative abundance; taxa with low relative abundance are grouped as “others.”

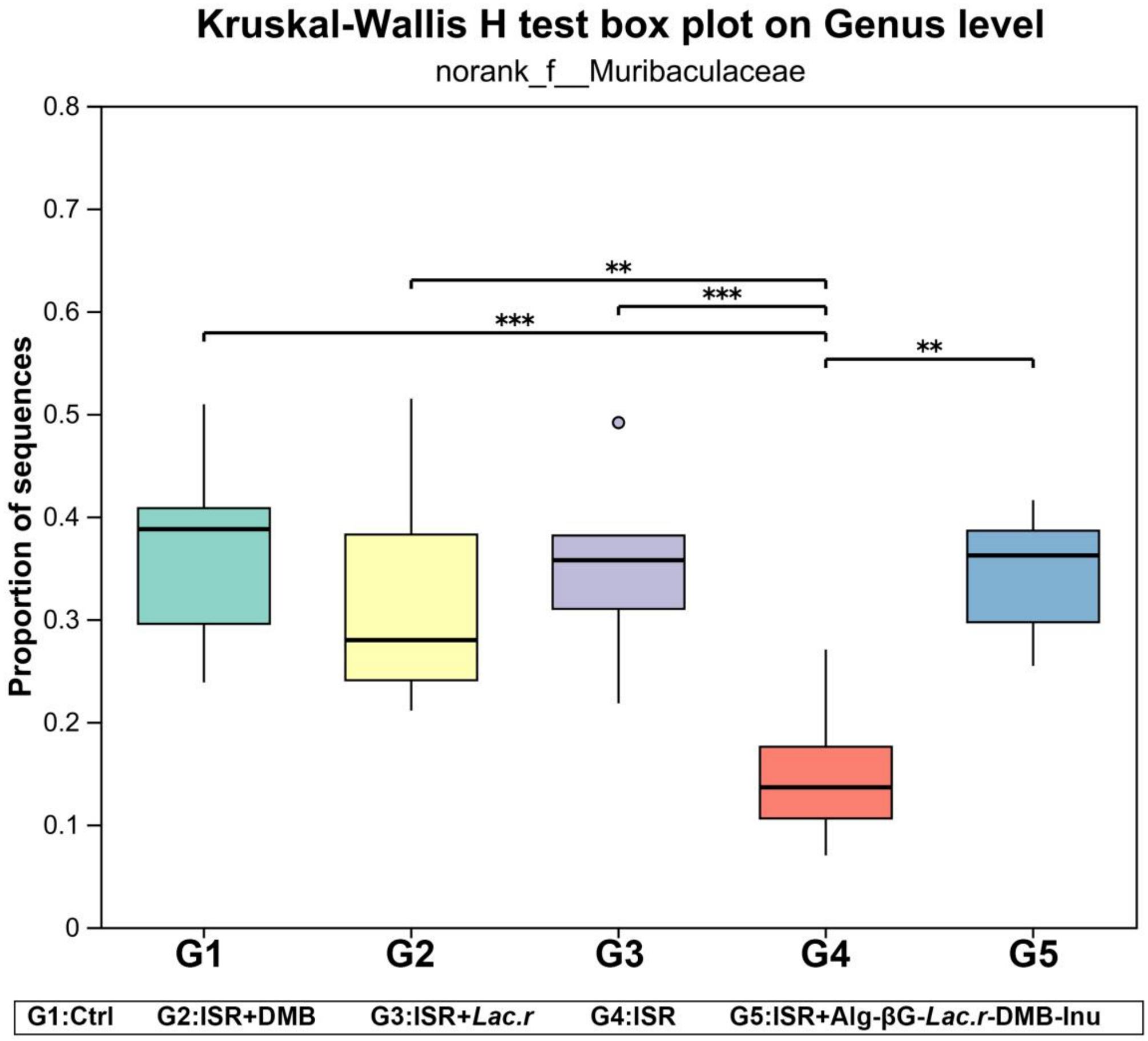

Fig. S43 Genus-level abundance analysis of *norank f Muribaculaceae* across treatment groups in in-stent restenosis animal models. Box plots show the relative abundance of *norank f Muribaculaceae* at the genus level in different groups. Group-wise statistical analysis was performed using the Kruskal–Wallis H test followed by pairwise multiple comparisons. Statistical significance levels are indicated on the plot. Boxes represent the interquartile range; center lines denote medians; whiskers indicate the data range excluding outliers.

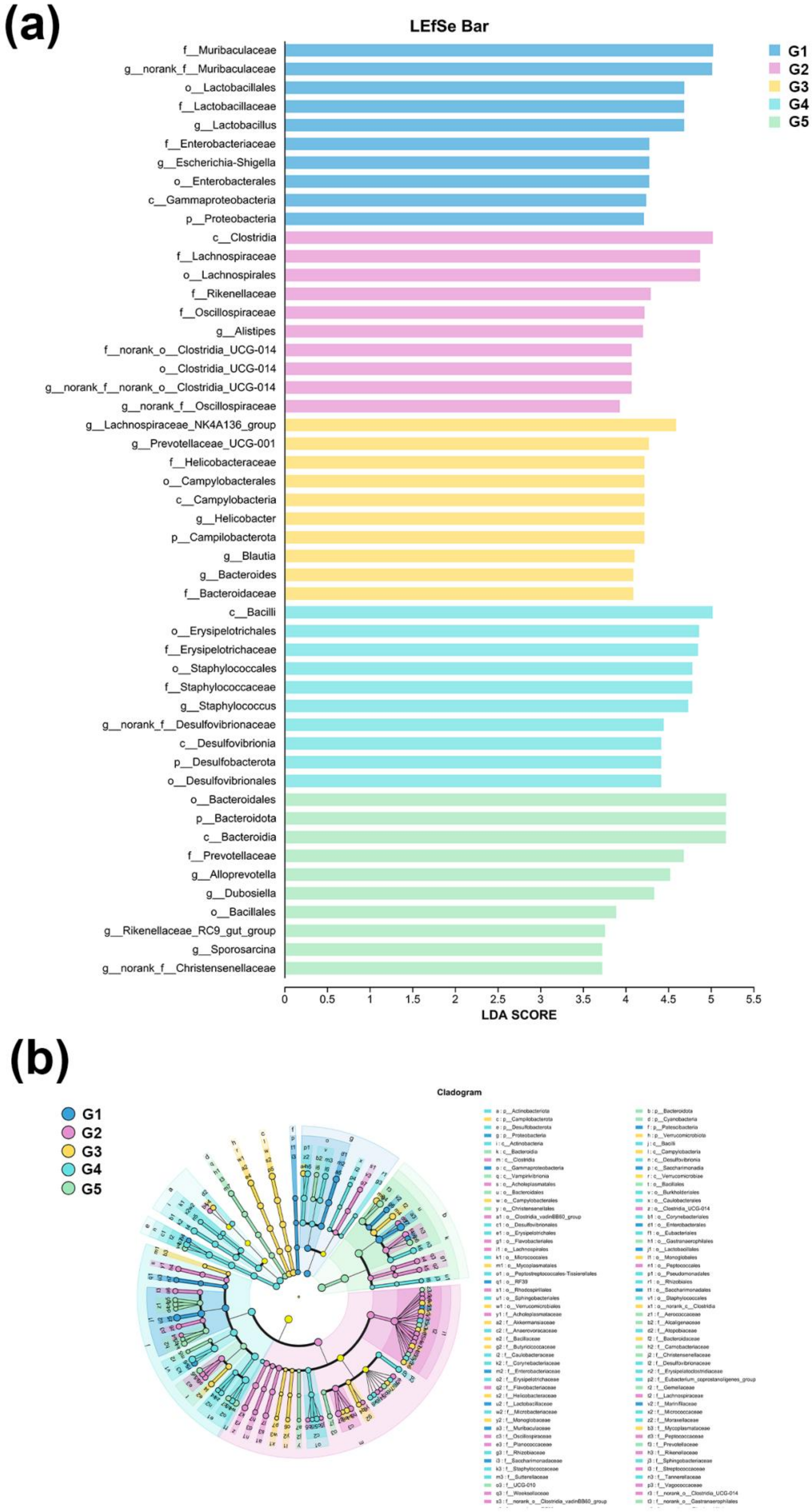

G1:Ctrl	G2:ISR+DMB	G3:ISR+*Lac.r*	G4:ISR	G5:ISR+Alg-βG-*Lac.r*-DMB-Inu

Fig. S44 Linear discriminant analysis effect size (LefSe)-based analysis of gut microbial taxa across treatment groups in in-stent restenosis animal models. (a) Linear discriminant analysis score bar plot generated from LEfSe analysis at multiple taxonomic levels. **(b)** Cladogram depicting the taxonomic hierarchy of microbial features identified by LEfSe analysis spanning from phylum to genus levels.

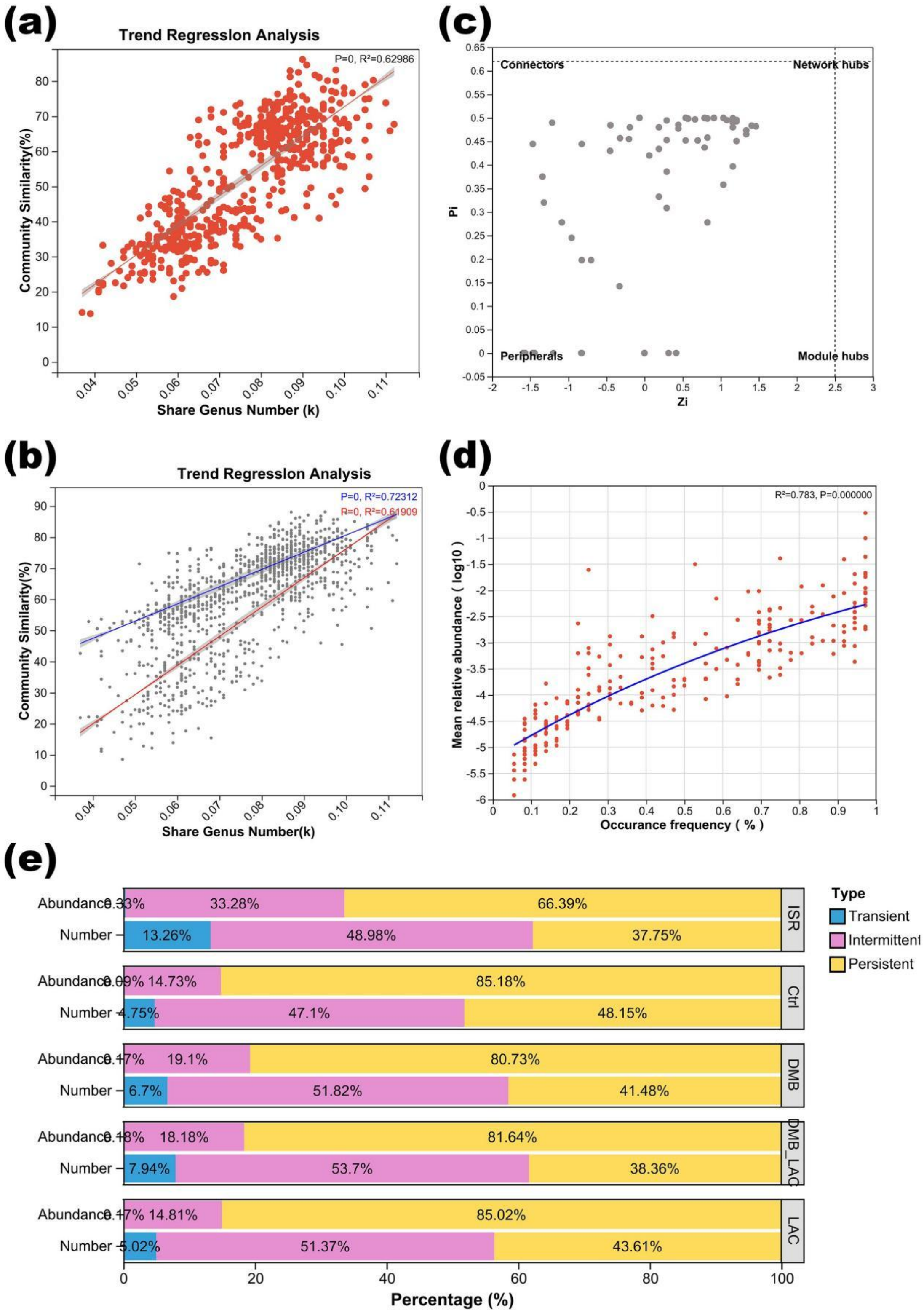

Fig. S45 Ecological stability and persistence analyses of gut microbial communities in in-stent restenosis animal models. (a) Trend regression analysis examining the relationship between community similarity and the number of shared

genera (k). **(b)** Group-stratified trend regression analysis of community similarity as a function of shared genus number. **(c)** Zi-Pi plot based on microbial co-occurrence network analysis, classifying genera according to within-module connectivity (Zi) and among-module connectivity (Pi). **(d)** The relationship between genus occurrence frequency and mean relative abundance (log-transformed). **(e)** Proportional distribution of taxa classified as transient, intermittent, or persistent based on occurrence frequency and abundance, presented at both abundance-weighted and number-based levels for each group.

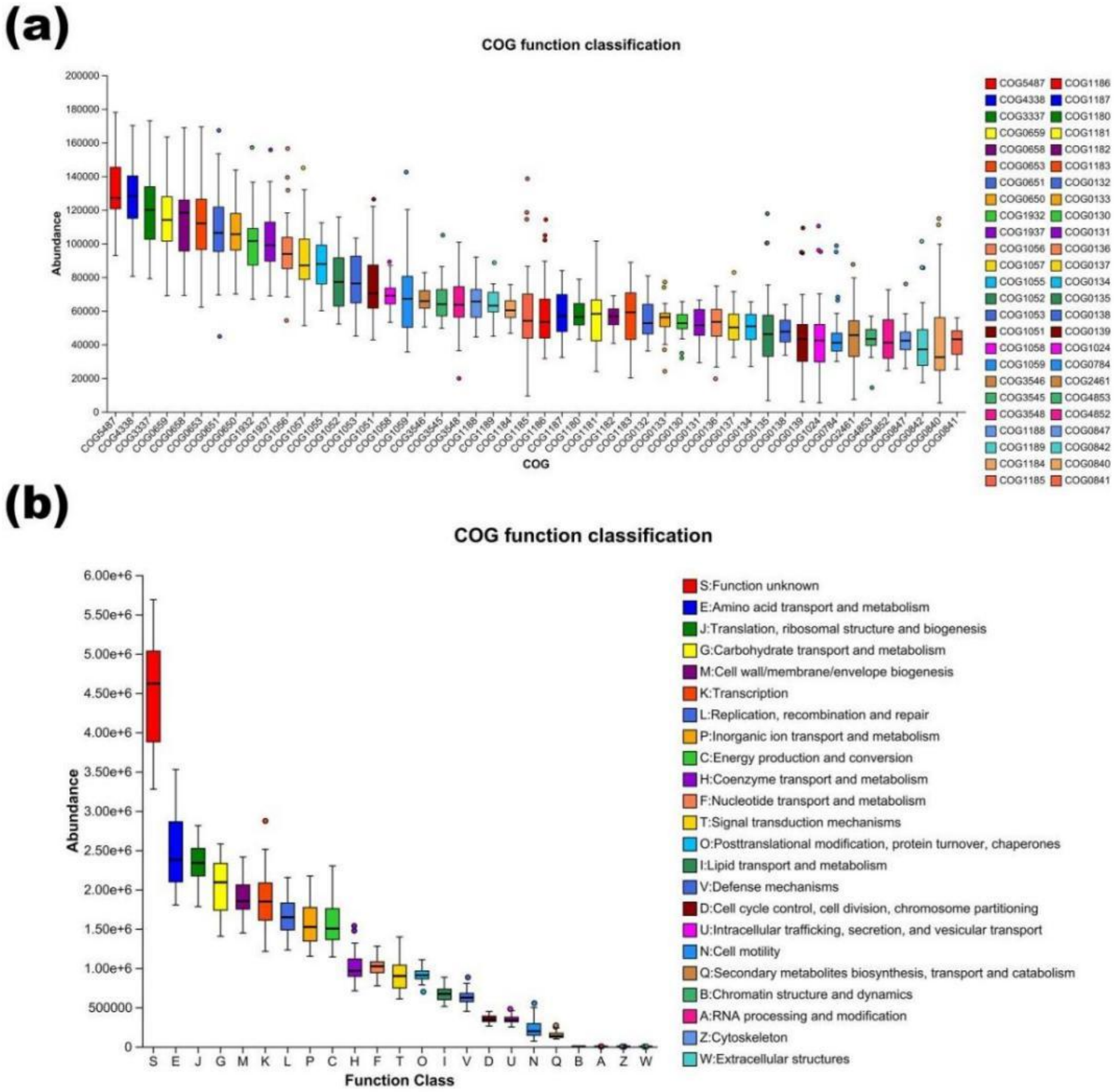

Fig. S46 Clusters of Orthologous Groups (COGs)-based functional profiling of gut microbial communities in in-stent restenosis animal models. **(a)** Abundance distribution of individual COGs across samples at the COG level. **(b)** Abundance distribution of COG functional classes aggregated into major functional categories across samples.

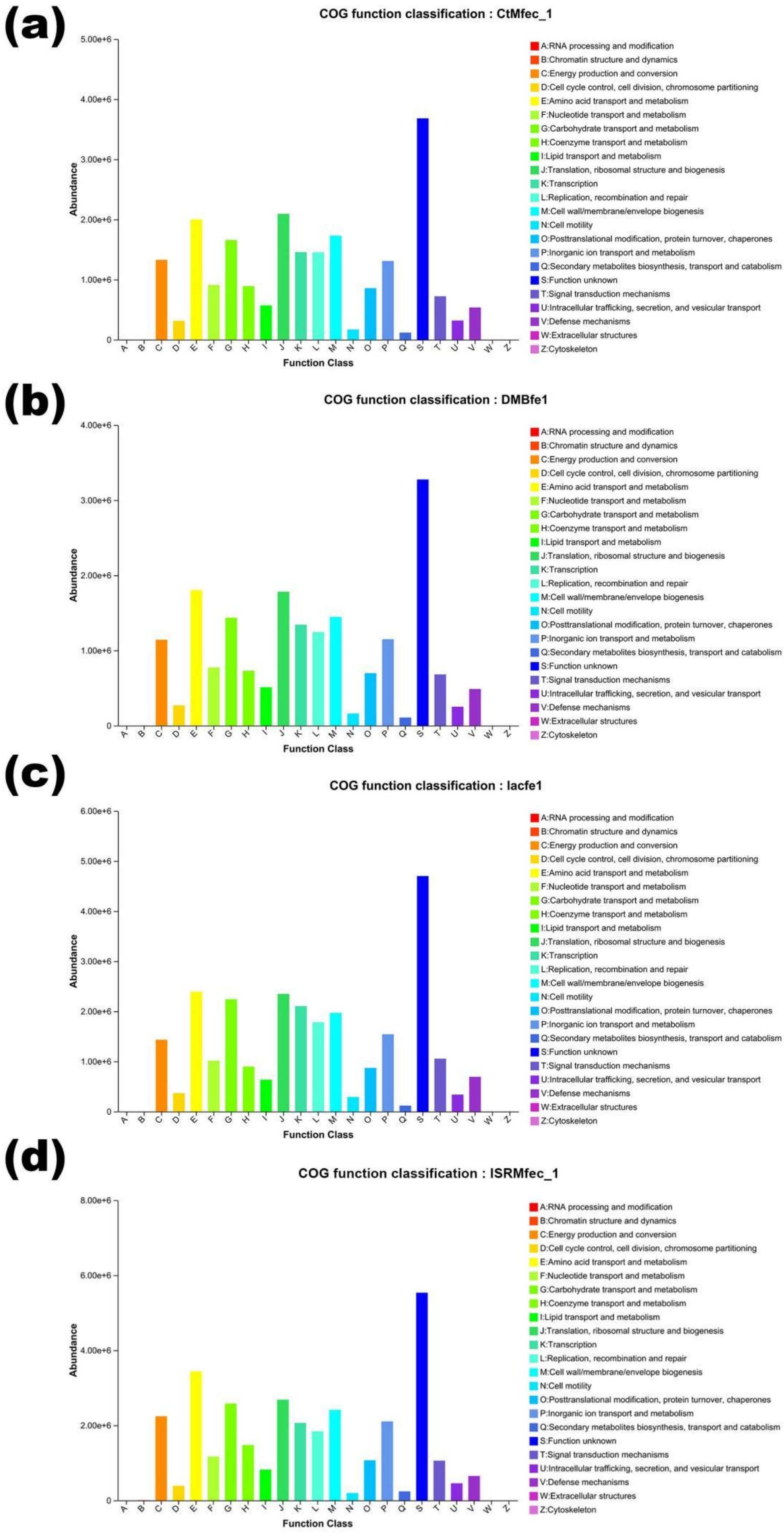

(a)
COG function classification : CtMfec_1
5.00e+6
4.00e+6
3.00e+6
2.00e+6
1.00e+6
0
Abundance
A B C D E F G H I J K L M N O P Q S T U V W Z
Function Class
A:RNA processing and modification
B:Chromatin structure and dynamics
C:Energy production and conversion
D:Cell cycle control, cell division, chromosome partitioning
E:Amino acid transport and metabolism
F:Nucleotide transport and metabolism
G:Carbohydrate transport and metabolism
H:Coenzyme transport and metabolism
I:Lipid transport and metabolism
J:Translation, ribosomal structure and biogenesis
K:Transcription
L:Replication, recombination and repair
M:Cell wall/membrane/envelope biogenesis
N:Cell motility
O:Posttranslational modification, protein turnover, chaperones
P:Inorganic ion transport and metabolism
Q:Secondary metabolites biosynthesis, transport and catabolism
S:Function unknown
T:Signal transduction mechanisms
U:Intracellular trafficking, secretion, and vesicular transport
V:Defense mechanisms
W:Extracellular structures
Z:Cytoskeleton
(b)
COG function classification : DMBfe1
4.00e+6
3.00e+6
2.00e+6
1.00e+6
0
Abundance
A B C D E F G H I J K L M N O P Q S T U V W Z
Function Class
A:RNA processing and modification
B:Chromatin structure and dynamics
C:Energy production and conversion
D:Cell cycle control, cell division, chromosome partitioning
E:Amino acid transport and metabolism
F:Nucleotide transport and metabolism
G:Carbohydrate transport and metabolism
H:Coenzyme transport and metabolism
I:Lipid transport and metabolism
J:Translation, ribosomal structure and biogenesis
K:Transcription
L:Replication, recombination and repair
M:Cell wall/membrane/envelope biogenesis
N:Cell motility
O:Posttranslational modification, protein turnover, chaperones
P:Inorganic ion transport and metabolism
Q:Secondary metabolites biosynthesis, transport and catabolism
S:Function unknown
T:Signal transduction mechanisms
U:Intracellular trafficking, secretion, and vesicular transport
V:Defense mechanisms
W:Extracellular structures
Z:Cytoskeleton
(c)
COG function classification : lacfe1
6.00e+6
5.00e+6
4.00e+6
3.00e+6
2.00e+6
1.00e+6
0
Abundance
A B C D E F G H I J K L M N O P Q S T U V W Z
Function Class
A:RNA processing and modification
B:Chromatin structure and dynamics
C:Energy production and conversion
D:Cell cycle control, cell division, chromosome partitioning
E:Amino acid transport and metabolism
F:Nucleotide transport and metabolism
G:Carbohydrate transport and metabolism
H:Coenzyme transport and metabolism
I:Lipid transport and metabolism
J:Translation, ribosomal structure and biogenesis
K:Transcription
L:Replication, recombination and repair
M:Cell wall/membrane/envelope biogenesis
N:Cell motility
O:Posttranslational modification, protein turnover, chaperones
P:Inorganic ion transport and metabolism
Q:Secondary metabolites biosynthesis, transport and catabolism
S:Function unknown
T:Signal transduction mechanisms
U:Intracellular trafficking, secretion, and vesicular transport
V:Defense mechanisms
W:Extracellular structures
Z:Cytoskeleton
(d)
COG function classification : ISRMfec_1
8.00e+6
6.00e+6
4.00e+6
2.00e+6
0
Abundance
A B C D E F G H I J K L M N O P Q S T U V W Z
Function Class
A:RNA processing and modification
B:Chromatin structure and dynamics
C:Energy production and conversion
D:Cell cycle control, cell division, chromosome partitioning
E:Amino acid transport and metabolism
F:Nucleotide transport and metabolism
G:Carbohydrate transport and metabolism
H:Coenzyme transport and metabolism
I:Lipid transport and metabolism
J:Translation, ribosomal structure and biogenesis
K:Transcription
L:Replication, recombination and repair
M:Cell wall/membrane/envelope biogenesis
N:Cell motility
O:Posttranslational modification, protein turnover, chaperones
P:Inorganic ion transport and metabolism
Q:Secondary metabolites biosynthesis, transport and catabolism
S:Function unknown
T:Signal transduction mechanisms
U:Intracellular trafficking, secretion, and vesicular transport
V:Defense mechanisms
W:Extracellular structures
Z:Cytoskeleton

Fig. S47 Clusters of Orthologous Groups (COGs) functional classification of predicted microbial gene profiles in in-stent restenosis animal models. (a–d) COG functional classification of predicted microbial gene abundance in representative samples from different treatment groups, including CtrlMfec_1, DMBfec_1, LACfec_1, and ASMfec_1.

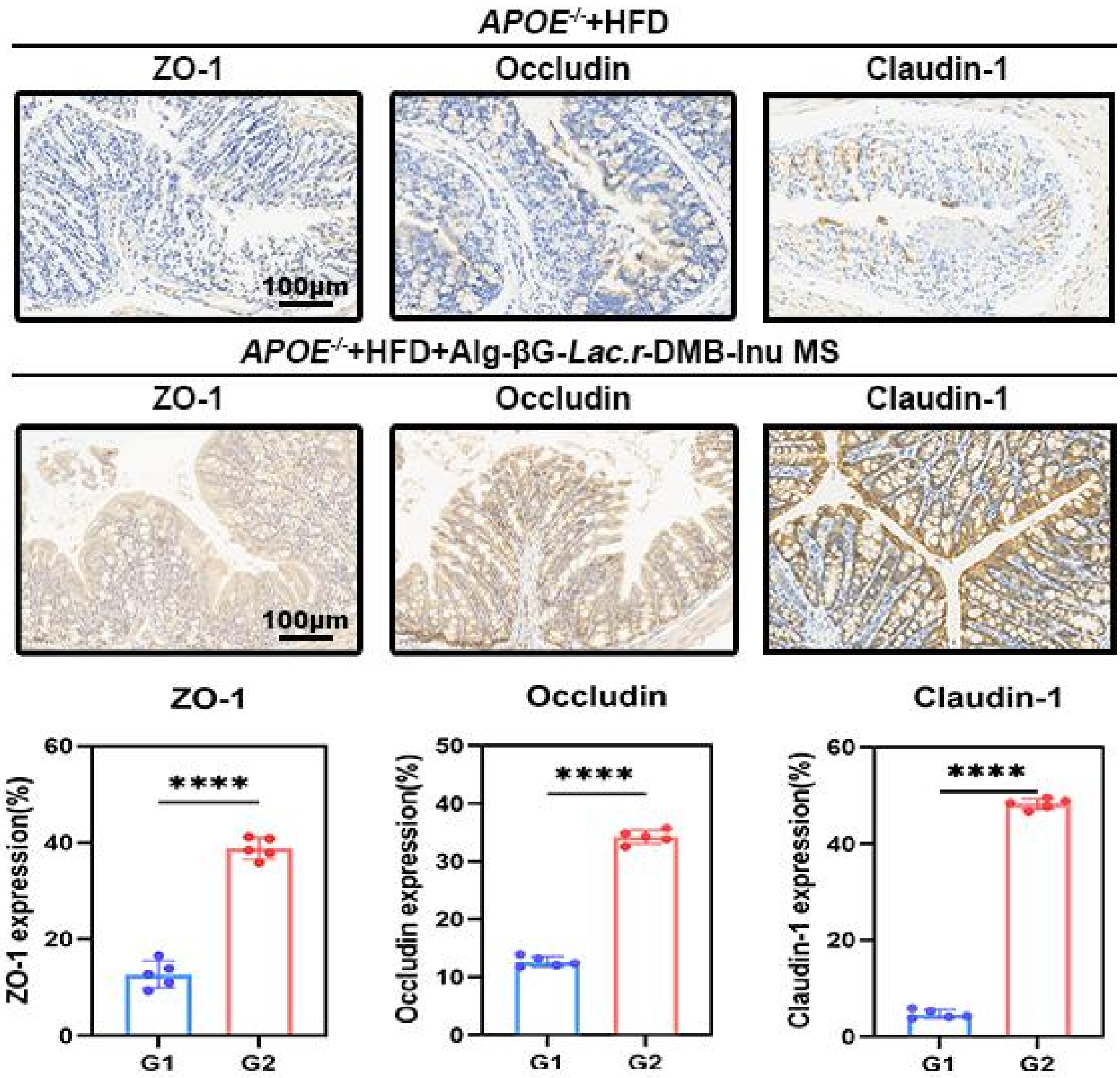

Fig. S48 Immunohistochemical analysis of tight junction proteins in colonic tissues. Representative immunohistochemical staining images of ZO-1, Occludin, and Claudin-1 in colonic sections from *ApoE*$^{-/-}$ mice fed a high-fat diet (*ApoE*$^{-/-}$ + HFD, G1) and those treated with Alg–βG–*Lac.r*–DMB–Inu MS (*ApoE*$^{-/-}$ + HFD + Alg–βG–*Lac.r*–DMB–Inu microspheres, G2). Corresponding quantitative analyses of the positive staining area for each tight junction protein shown below. Analyses were performed to evaluate colonic epithelial barrier-associated protein expression under different treatment conditions.

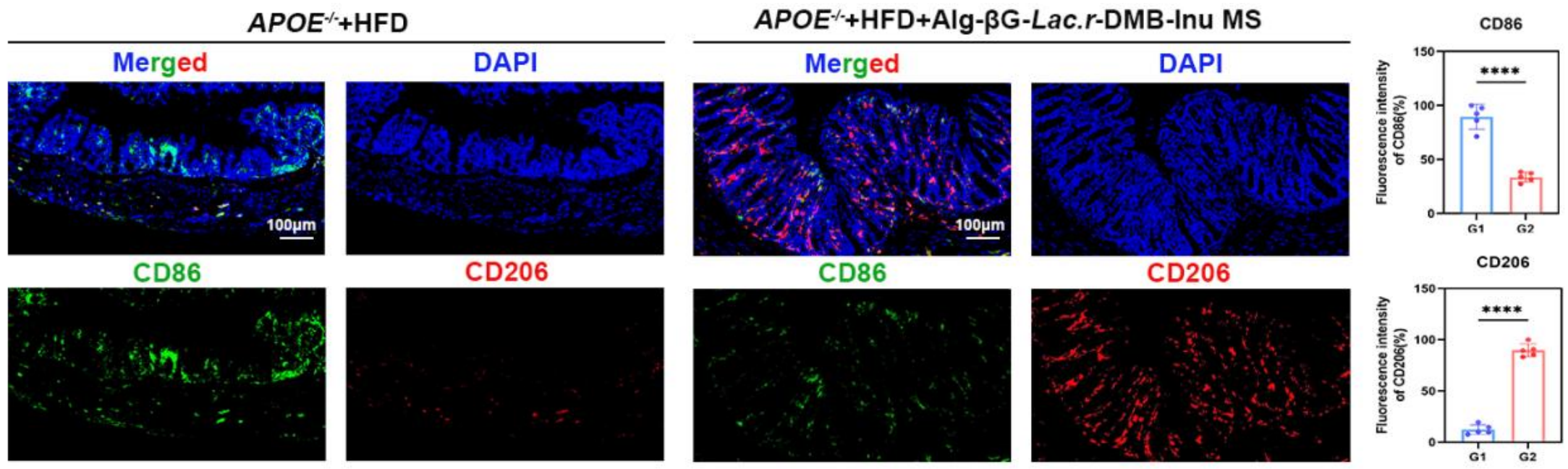

Fig. S49 Dual immunofluorescence analysis of macrophage polarization markers in colonic tissues. Representative dual immunofluorescence images of CD86 and CD206 in colonic sections from *ApoE*$^{-/-}$ mice fed a high-fat diet *ApoE*$^{-/-}$ + HFD, G1) and those treated with Alg–βG–*Lac.r*–DMB–Inu MS (*ApoE*$^{-/-}$ + HFD + Alg–βG–*Lac.r*–DMB–Inu microspheres, G2). Nuclei were counterstained with DAPI. Merged and single-channel images are shown. Quantitative analysis of fluorescence intensity for CD86 and CD206 is presented on the right to assess macrophage phenotype distribution in the colon under different treatment conditions.

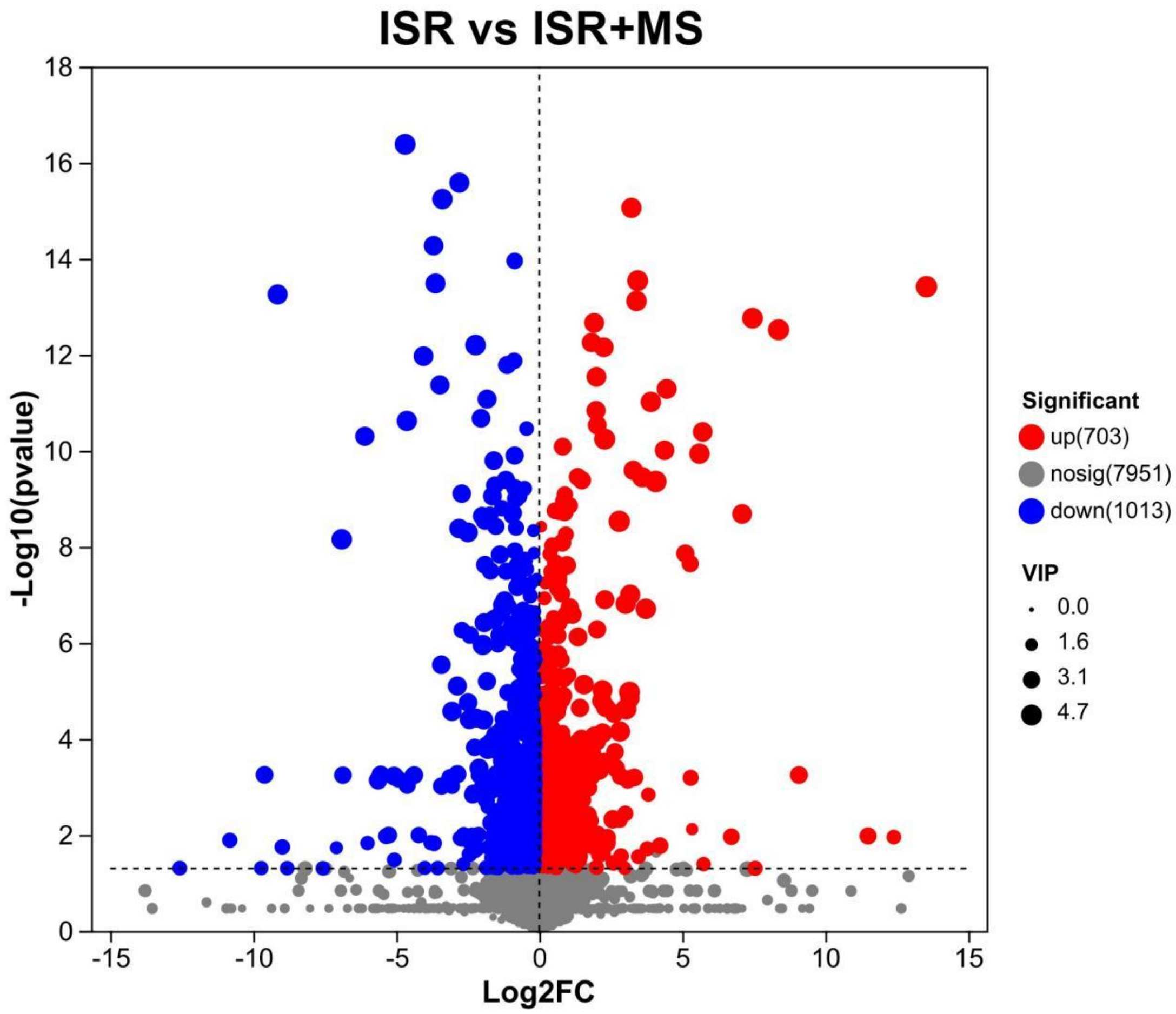

Fig. S50 Differential metabolite analysis between in-stent restenosis (ISR) and ISR + microsphere treatment. Volcano plot showing differentially abundant metabolites between ISR animals and those treated with Alg–βG–*Lac.r*–DMB–Inu microspheres (ISR + MS). The x-axis represents $\log_2$ fold change (ISR + MS vs. ISR); the y-axis represents $-\log_{10}$(p value). Metabolites significantly increased or decreased following microsphere treatment are highlighted in red and blue, respectively; nonsignificant features are in gray. Point size reflects the variable importance in projection score derived from multivariate analysis. Horizontal and vertical dashed lines indicate the thresholds used for statistical significance and fold change filtering.

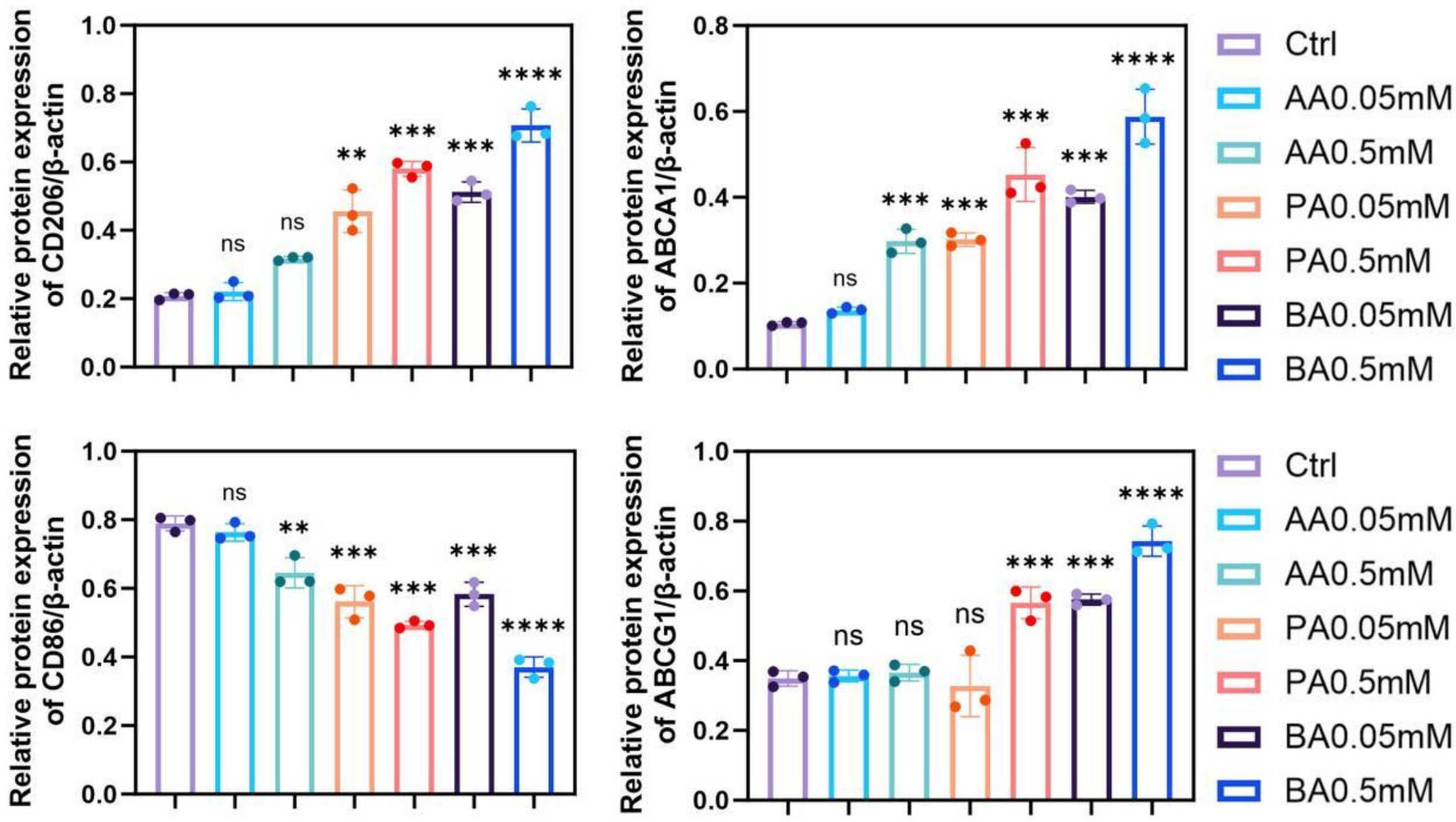

Fig. S51 Quantitative analysis of Western blot results corresponding to Fig. 9(j). Bar graphs showing the densitometric quantification of CD206, CD86, ABCA1, and ABCG1 protein expression in macrophages under the indicated treatment conditions. Protein levels were normalized to β-actin and are presented relative to the control group. Data represent the statistical analysis of the Western blot bands shown in Fig. 9j. Statistical significance was determined by one-way ANOVA with multiple comparison tests: ns, not significant; $^{**}p < 0.01$; $^{***}p < 0.001$; $^{****}p < 0.0001$.

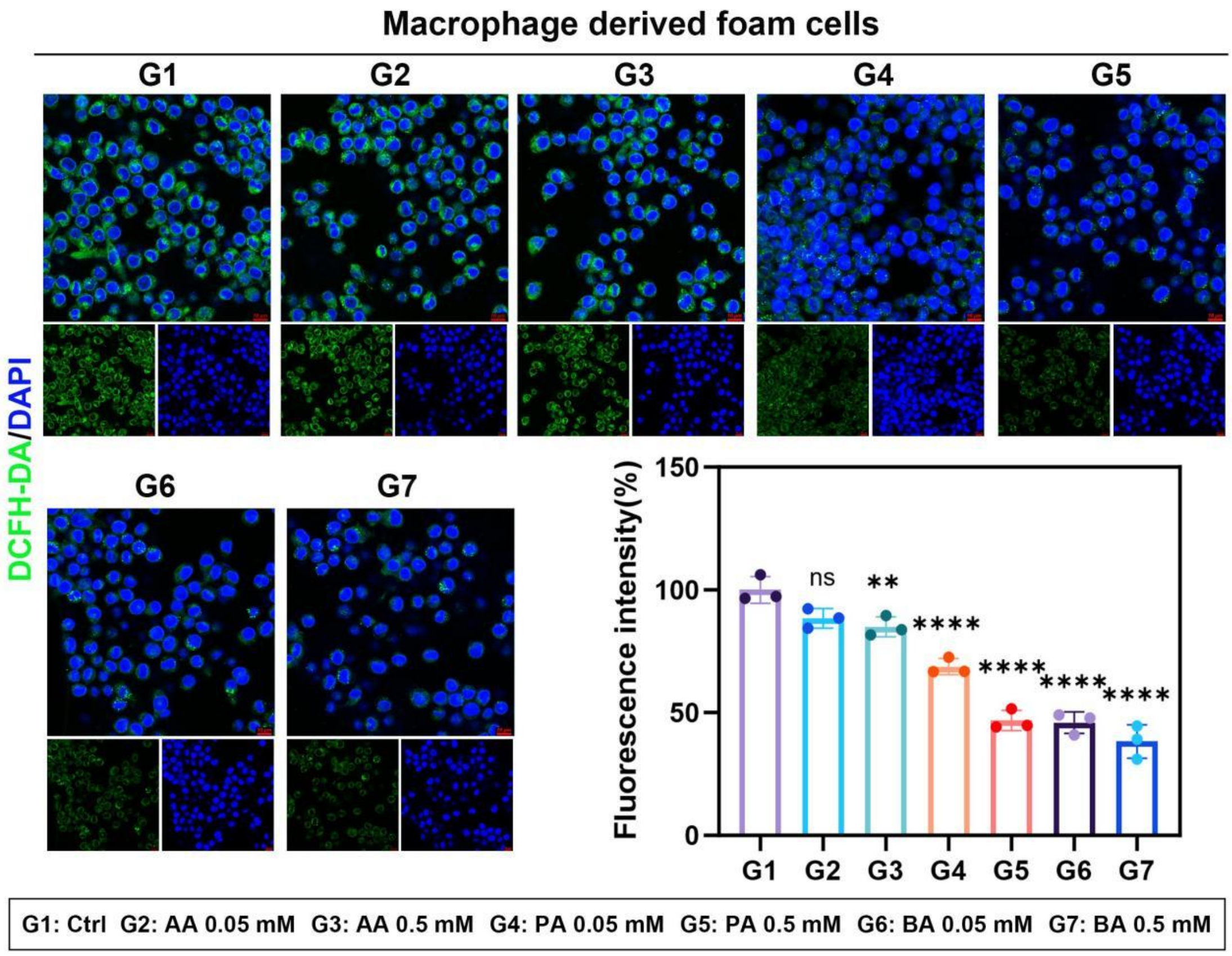

Fig. S52 Effects of representative short-chain fatty acids on intracellular reactive oxygen species (ROS) levels in macrophage-derived foam cells. Representative confocal fluorescence images of macrophage-derived foam cells stained with DCFH-DA (green) to assess intracellular ROS levels, with DAPI (blue) used for nuclear counterstaining. Cells were treated with acetic acid (AA), propionic acid (PA), or butyric acid (BA) at two concentration gradients (0.05 and 0.5 mM), with untreated cells serving as the control (Ctrl). The right panel shows quantitative analysis of DCFH-DA fluorescence intensity, normalized to the Ctrl group. Data are presented as mean ± SEM. Statistical significance was determined by one-way ANOVA with multiple comparison tests: ns, not significant; $^{**}p < 0.01$; $^{****}p < 0.0001$. G1, Ctrl; G2, AA 0.05 mM; G3, AA 0.5 mM; G4, PA 0.05 mM; G5, PA 0.5 mM; G6, BA 0.05 mM; G7, BA 0.5 mM.

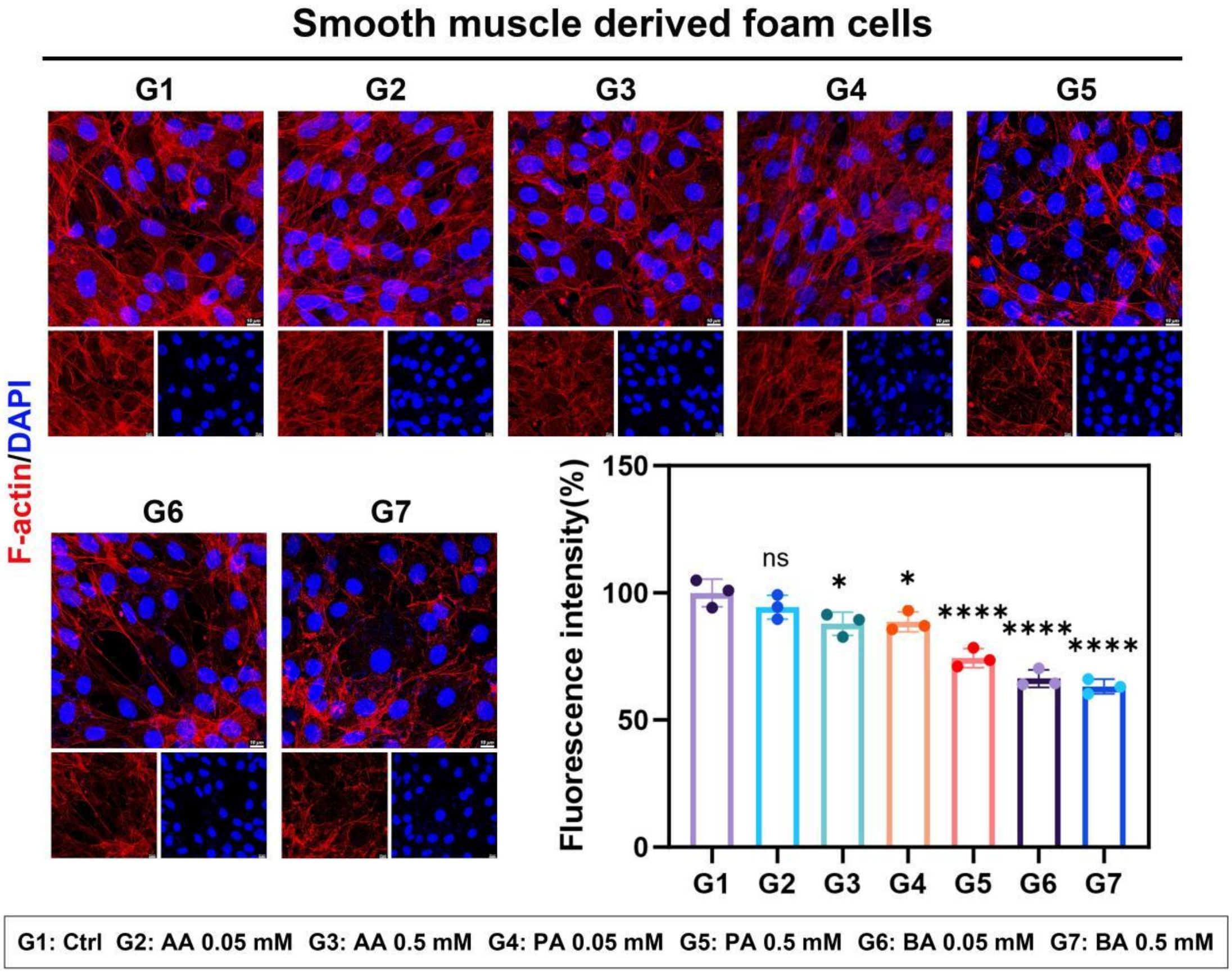

Fig. S53 Effects of short-chain fatty acids on proliferative activity-associated cytoskeletal remodeling in smooth muscle-derived foam cells. Representative confocal images of smooth muscle-derived foam cells stained with F-actin (red) and DAPI (blue). Cells were treated with acetic acid (AA; 0.05 and 0.5 mM), propionic acid (PA; 0.05 and 0.5 mM), or butyric acid (BA; 0.05 and 0.5 mM), with an untreated control (Ctrl). The right panel shows quantification of F-actin fluorescence intensity (normalized to Ctrl) as a phenotypic readout of cytoskeletal remodeling associated with smooth muscle cell proliferative activation. Data are presented as mean ± SEM. Statistical significance was assessed by one-way ANOVA with multiple comparisons: ns, not significant; $*p < 0.05$; $**p < 0.01$; $****p < 0.0001$. G1, Ctrl; G2, AA 0.05 mM; G3, AA 0.5 mM; G4, PA 0.05 mM; G5, PA 0.5 mM; G6, BA 0.05 mM; G7, BA 0.5 mM.

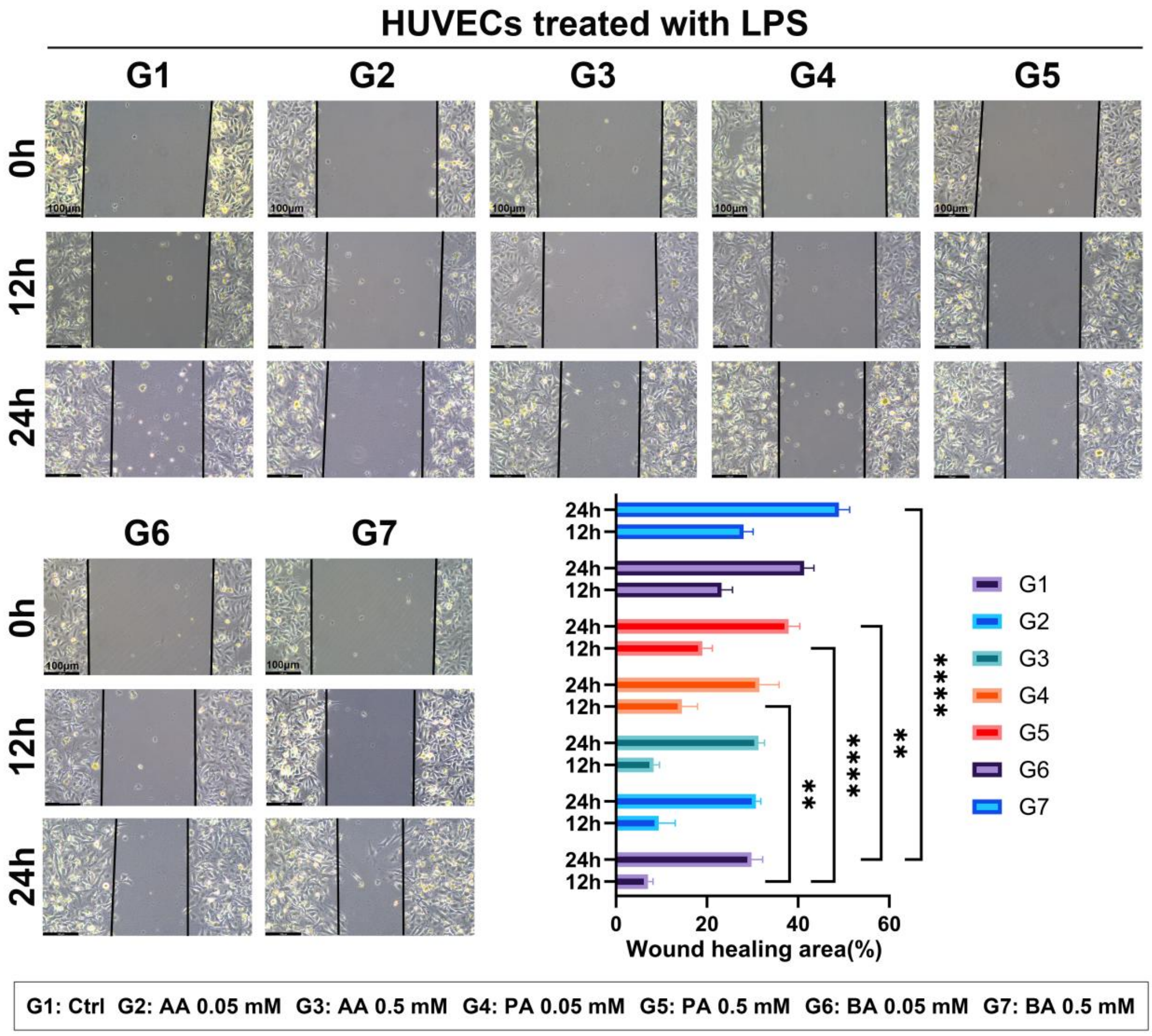

Fig. S54 Effects of representative short-chain fatty acids on endothelial wound healing under inflammatory conditions. Representative phase-contrast images of scratch wound-healing assays performed in lipopolysaccharide (LPS)-challenged human umbilical vein endothelial cells treated with acetic acid (AA), propionic acid (PA), or butyric acid (BA). Images were acquired at 0, 12, and 24 h following scratch generation to assess endothelial migration and monolayer repair dynamics. Quantitative analysis of wound healing area (%) at 12 and 24 h is shown on the right. Cells were treated with AA, PA, or BA at two concentration gradients (0.05 and 0.5 mM), with untreated LPS-stimulated cells as the control (Ctrl). Data are presented as mean ± SEM. Statistical significance was determined by one-way ANOVA with multiple comparison tests. G1, Ctrl (LPS only); G2, AA 0.05 mM; G3, AA 0.5 mM; G4, PA 0.05 mM; G5, PA 0.5 mM; G6, BA 0.05 mM; G7, BA 0.5 mM.

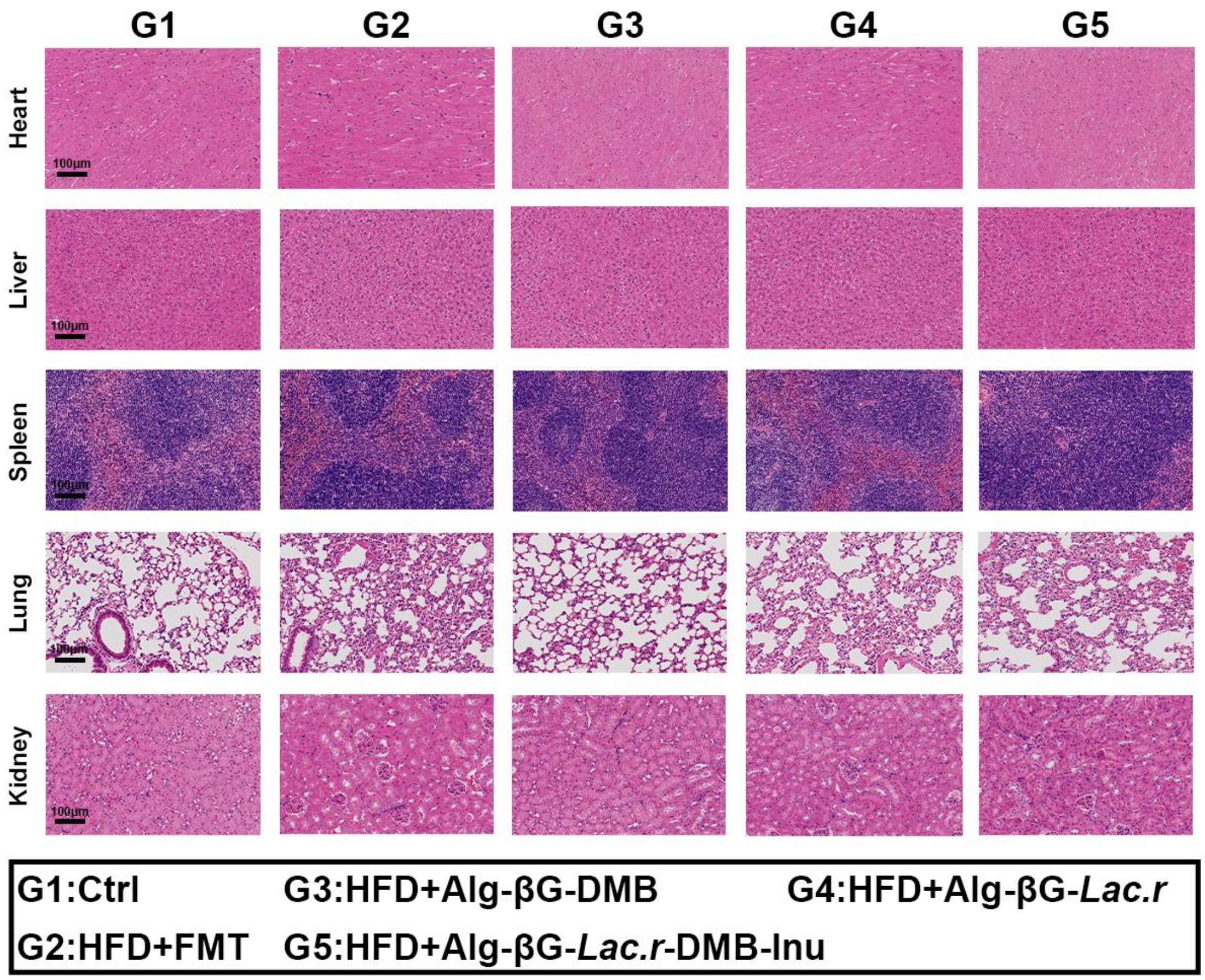

Fig. S55 *In vivo* biosafety evaluation of Alg–βG-based microsphere treatments. Representative hematoxylin and eosin-stained histological sections of major organs (including heart, liver, spleen, lungs, and kidneys) collected from mice in different treatment groups. Tissues were harvested at the experimental endpoint and processed for histopathological examination to assess potential off-target toxicity and organ damage following long-term microsphere administration. Scale bar: 100 μm. G1, Ctrl; G2, HFD + FMT; G3, HFD + Alg–βG–DMB; G4, HFD + Alg–βG–*Lac.r*; G5, HFD + Alg–βG–*Lac.r*–DMB–Inu microspheres.

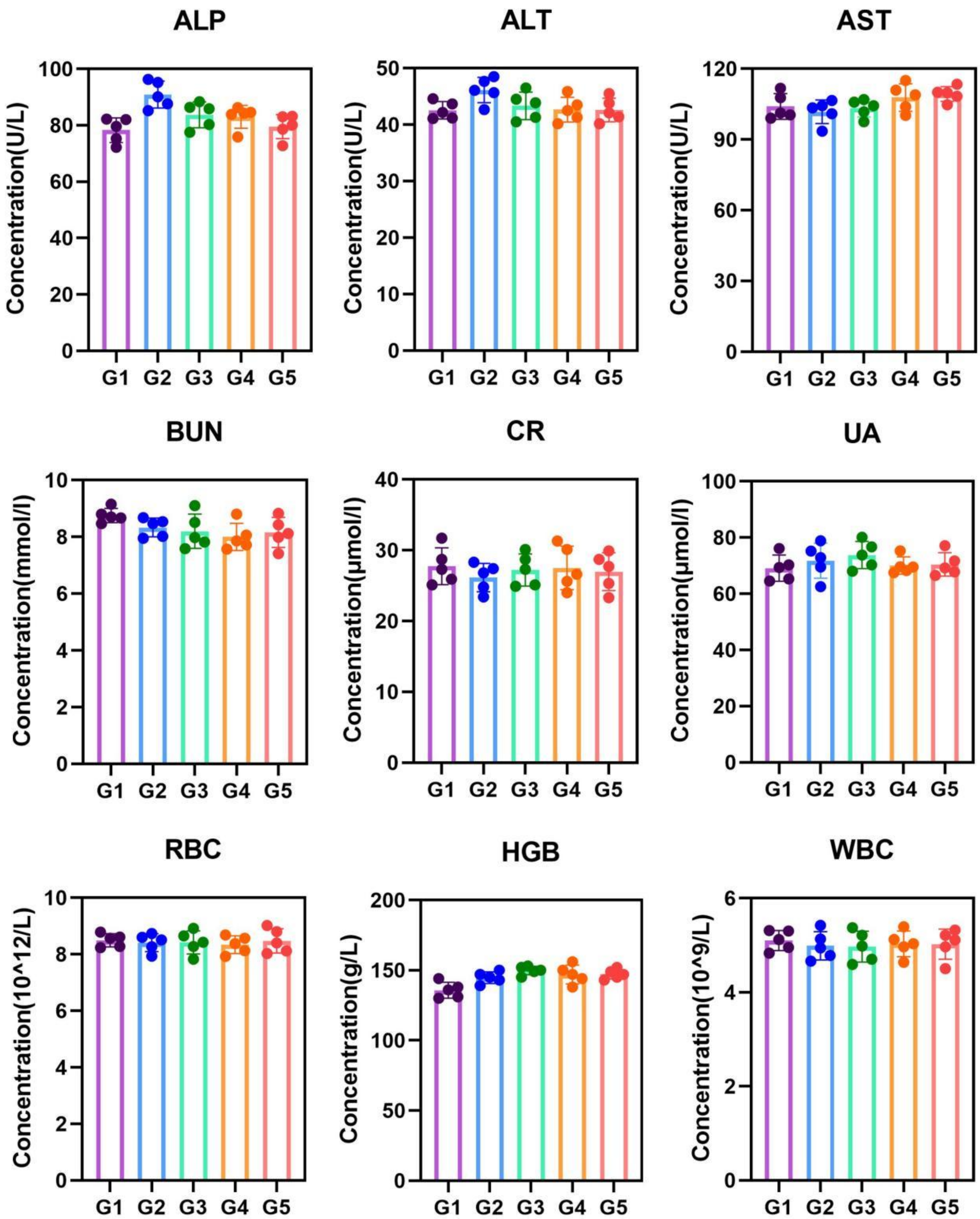

Fig. S56 Hematological and serum biochemical safety evaluation following microsphere treatment. Comprehensive hematological and serum biochemical analyses were performed to evaluate the systemic biosafety of Alg-βG-based

microsphere treatments. Serum liver function indicators (including alkaline phosphatase, alanine aminotransferase, and aspartate aminotransferase) and renal function-related parameters (including blood urea nitrogen, creatinine, and uric acid) were quantified. Routine hematological parameters (including red blood cell count, hemoglobin concentration, and white blood cell count) were measured. Samples were collected at the experimental endpoint from mice in different treatment groups. Data are presented as individual values with mean ± SEM. G1, Ctrl; G2, HFD + FMT; G3, HFD + Alg–βG–DMB; G4, HFD + Alg–βG–*Lac.r*; G5: HFD + Alg–βG–*Lac.r*–DMB–Inu microspheres**.**

Table S1.Kits and biochemical reagents.

Reagent/Kit	Source	Cat No.	Application
MRS Broth	Solarbio, Beijing, China	M8540-250g	Bacterial culture
3,3-Dimethyl-1-butanol (DMB)	Shanghai Yuanye Bio-Technology, Shanghai, China	Y96575-5g	Microsphere fabrication
Inulin	Shanghai Yuanye Bio-Technology, Shanghai, China	S11143-5g	Microsphere fabrication
Calcein-AM/PI Live/Dead Cell Viability Kit	Beyotime Biotechnology, Shanghai, China	C2015S	Cytocompatibili-ty assay
SYTO9/PI Live and Dead Bacteria Stain Kit	Thermo Fisher Scientific, Waltham, MA, USA	L7007	Antibacterial assay
DCFH-DA (ROS probe)	Beyotime Biotechnology, Shanghai, China	S0033S	ROS detection
DAPI	Beyotime Biotechnology, Shanghai, China	C1005	Nuclear counterstaining
IL-1β ELISA Kit	Wuhan EIAab Science, Wuhan, China	MM-0040M1	Cytokine quantification
TNF-α ELISA Kit	Wuhan EIAab Science, Wuhan, China	MM-0132M1	Cytokine quantification

IL-18 ELISA Kit	Wuhan EIAab Science, Wuhan, China	MM-0169M1	Cytokine quantification
TMAO ELISA Kit	Wuhan Jilid Biotechnology, Wuhan, China	J25470	Metabolite quantification
Color PAGE Gel Rapid Preparation Kit	EpiZyme, Shanghai, China	PG112	SDS-PAGE
RevertAid First Strand cDNA Synthesis Kit	Thermo Fisher Scientific, Waltham, MA, USA	K1622	RT-qPCR
DNase I	Thermo Fisher Scientific, Waltham, MA, USA	EN0521	Genomic DNA elimination
RNA-Quick Purification Kit	YEASEN Biotechnology, Shanghai, China	RN001	RNA extraction
SYBR Green Master Mix	Vazyme Biotech, Nanjing, China	Q711-02	RT-qPCR
Oxidized low-density lipoprotein (oxLDL)	YEASEN Biotechnology, Shanghai, China	20605ES05	Foam cell induction
acetic acid (AA) ELISA Kit	Wuhan EIAab Science, Wuhan, China	MM-926697O1	short chain fatty acids quantification
propionic acid (PA)	Wuhan EIAab	MM-926981O	short chain fatty acids

ELISA Kit	Science, Wuhan, China		quantification
butyric acid (BA) ELISA Kit	Wuhan EIAab Science, Wuhan, China	MM-926691O1	short chain fatty acids quantification

Table S2.Antibodies.

Antibody	Source	Catalog No.	RRID (if applicable)	Application
AF488-conjugated anti-mouse F4/80	BioLegend, San Diego, CA, USA	123120	AB_893491	Flow cytometry
PerCP-Cy5.5 anti-mouse CD11b	BioLegend, San Diego, CA, USA	101228	AB_893232	Flow cytometry
PE anti-mouse CD86	BioLegend, San Diego, CA, USA	105008	AB_313151	Flow cytometry / IHC
APC anti-mouse CD206	BioLegend, San Diego, CA, USA	141708	AB_10900231	Flow cytometry / IHC
FITC anti-mouse CD3	BioLegend, San Diego, CA, USA	100305	AB_312671	Flow cytometry
PE anti-mouse CD4	BioLegend, San Diego, CA, USA	100407	AB_312693	Flow cytometry
APC anti-mouse	BioLegend,	100711	AB_312751	Flow cytometry

CD8	San Diego, CA, USA			
FITC anti-mouse Foxp3	BioLegend, San Diego, CA, USA	126406	AB_1089114	Flow cytometry
Anti-ABCA1	Abcam, Cambridge, UK	7360	AB_305880	Western blot / Histology
Anti-ABCG1	Abcam, Cambridge, UK	52617	AB_867471	Western blot / Histology
Anti-CD86	ABclonal, Wuhan, China	A21198	AB_3712536	Western blot / Histology
Anti-CD206	Proteintech, Wuhan, China	18704-1-AP	AB_10597232	Western blot / Histology
Anti-ZO-1	Proteintech, Wuhan, China	21773-1-AP	AB_10733242	Western blot / Histology
Anti-ZO-3	Abcam, Cambridge, UK	191143	N/A	Western blot / Histology
Anti-Occludin	Proteintech, Wuhan, China	27260-1-AP	AB_2880820	Western blot / Histology
Anti-Claudin-1	Proteintech, Wuhan, China	28674-1-AP	AB_2881190	Western blot / Histology
Anti-LPS	Thermo Fisher Scientific, Waltham,	MA5-41631	AB_2899113	Western blot / Histology

	MA, USA			
Anti-β-actin	Santa Cruz Biotechnology, Dallas, TX, USA	SC-8432	AB_626630	Western blot
Anti-CD31	Proteintech, Wuhan, China	28083-1-AP	AB_2881055	Western blot / Histology
Anti-α-SMA	Proteintech, Wuhan, China	14395-1-AP	AB_2223009	Western blot / Histology

Table S3. The primer sequences used for qRT-PCR

Gene	Primer Direction	Sequence (5′ → 3′)
ABCA1	Forward	ACCCACCCTATGAACAACATGA
	Reverse	GAGTCGGGTAACGGAAACAGG
ABCG1	Forward	ATTCAGGGACCTTTCCTATTCGG
	Reverse	CTCACCACTATTGAACTTCCCG
INOS	Forward	CTCGCTGCATCGGCAGGATTC
	Reverse	AGTCATGCTTCCCATCGCTCC
ARG1	Forward	GTGGAAACTTGCATGGACAAC
	Reverse	AATCCTGGCACATCGGGAATC
IL4	Forward	TGACGGCACAGAGCTATTGA
	Reverse	TTTGGCACATCCATCTCCGT
IL10	Forward	GTGGAGCAGGTGAAGAGTGAT
	Reverse	ACACTCCAGGTGCAGAATAGC
IL12β	Forward	TGTGGAATGGCGTCTCTGTC
	Reverse	AGTTCAATGGGCAGGTTCTC
IL1β	Forward	GCCTTGGGCCTCAAAGGAAAGAATC
	Reverse	GGAAGACACAGATTCCATGGTGAAG
TNFα	Forward	ATAGCTCCCAGAAAAGCAAGC

	Reverse	CACCCCGAAGTTCAGTAGACA
GADPH	Forward	TGCACCACCAACTGCTTAG
	Reverse	GATGCAGGGATGATGTTC
